# Development of Anthraquinone Derivatives as Ectonucleoside Triphosphate Diphosphohydrolase (NTPDase) Inhibitors With Selectivity for NTPDase2 and NTPDase3

**DOI:** 10.3389/fphar.2020.01282

**Published:** 2020-08-27

**Authors:** Younis Baqi, Mahmoud Rashed, Laura Schäkel, Enas M. Malik, Julie Pelletier, Jean Sévigny, Amelie Fiene, Christa E. Müller

**Affiliations:** ^1^Department of Chemistry, Faculty of Science, Sultan Qaboos University, Muscat, Oman; ^2^PharmaCenter Bonn, Pharmaceutical Institute, Pharmaceutical & Medicinal Chemistry, University of Bonn, Bonn, Germany; ^3^Centre de Recherche du CHU de Québec–Université Laval, Québec, QC, Canada; ^4^Département de Microbiologie-Infectiologie et d’Immunologie, Faculté de Médecine, Université Laval, Québec, QC, Canada

**Keywords:** anthraquinone, CD39, inhibitor, metalloenzymes, neuroinflammation, NTPDase2, NTPDase3, synthesis

## Abstract

Ectonucleoside triphosphate diphosphohydrolases (NTPDases) catalyze the hydrolysis of nucleoside tri- and di-phosphates to mono-phosphates. The products are subsequently hydrolyzed by ecto-5′-nucleotidase (ecto-5′-NT) to nucleosides. NTPDase inhibitors have potential as novel drugs, e.g., for the treatment of inflammation, neurodegenerative diseases, and cancer. In this context, a series of anthraquinone derivatives structurally related to the anthraquinone dye reactive blue-2 (RB-2) was synthesized and evaluated as inhibitors of human NTPDases utilizing a malachite green assay. We identified several potent and selective inhibitors of human NTPDase2 and -3. Among the most potent NTPDase2 inhibitors were 1-amino-4-(9-phenanthrylamino)-9,10-dioxo-9,10-dihydroanthracene-2-sulfonate (20, PSB-16131, IC_50_ of 539 nM) and 1-amino-4-(3-chloro-4-phenylsulfanyl)phenylamino-9,10-dioxo-9,10-dihydroanthracene-2-sulfonate (48, PSB-2020, IC_50_ of 551 nM). The most potent NTPDase3 inhibitors were 1-amino-4-[3-(4,6-dichlorotriazin-2-ylamino)-4-sulfophenylamino]-9,10-dioxo-9,10-dihydroanthracene-2-sulfonate (42, PSB-1011, IC_50_ of 390 nM) and 1-amino-4-(3-carboxy-4-hydroxyphenylamino)-9,10-dioxo-9,10-dihydroanthracene-2-sulfonate (33, PSB-2046, IC_50_ of 723 nM). The best NTPDase2 inhibitor 20 showed a non-competitive inhibition type, while the NTPDase3 inhibitor 42 behaved as a mixed-type inhibitor. These potent compounds were found to be selective vs. other NTPDases. They will be useful tools for studying the roles of NTPDase2 and -3 in physiology and under pathological conditions.

## Introduction

Ectonucleotidases are membrane-bound metalloenzymes that affect extracellular nucleotide and nucleoside levels by catalyzing the hydrolysis of nucleotides to the corresponding nucleosides releasing inorganic phosphate or diphosphate (Dou et al., 2018; Le et al., 2019; Vuerich et al., 2019). There are four major subfamilies of ectonucleotidases: the ecto-nucleoside triphosphate diphosphohydrolases (NTPDases), the ecto-nucleotide pyrophosphatases/phosphodiesterases (NPPs), the alkaline phosphatases (APs), and the ecto-5′-nucleotidase (ecto-5′-NT, CD73) (Bonan, 2012; Al-Rashida and Iqbal, 2015; Baqi, 2015; Fiene et al., 2016; Le et al., 2019). In inflammatory processes, there may be a massive increase in extracellular ATP concentrations causing proinflammatory immune responses *via* P2X and P2Y receptors. ATP can be hydrolyzed by NTPDases, or at very high concentrations also by APs, *via* ADP to AMP. Alternatively, ATP can be cleaved directly to AMP and diphosphate (pyrophosphate) by NPPs (Lee and Müller, 2017). The resulting AMP can eventually be hydrolyzed by ecto-5’-NT yielding adenosine, which induces antiinflammatory effects *via* activation of P1 (adenosine) receptors (King et al., 2006; Burnstock, 2018; Antonioli et al., 2019; Müller et al., 2020).

Several studies reported that NTPDase2 is localized in specialized astrocytes in rodent brain, such as laminar astrocytes associated with fiber tracts in the brain stem and cerebrum (Braun et al., 2003; Braun et al., 2004), tanycytes, non-stellate astrocytes in the gray matter of discrete regions, like habenula (Gampe et al., 2012), satellite astrocytes in the dorsal root ganglion (Braun et al., 2003), and astrocyte-like progenitor cells of the subventricular zone (SVZ) of the lateral ventricle (Shukla et al., 2005; Mishra et al., 2006; Gampe et al., 2015). NTPDase3 is localized in the midline regions: in the thalamus, hypothalamus, and the medulla oblongata (Belcher et al., 2006; Grković et al., 2016). Both enzymes, NTPDase2, and to a lesser extent also NTPDase3, preferentially catalyze the dephosphorylation of ATP to ADP, generating the physiological ligand for P2Y_1_, P2Y_12_, and P2Y_13_ receptors (Kukulski et al., 2005; Zimmermann et al., 2012; Burnstock, 2020; Müller et al., 2020). Therefore, NTPDase2 and -3 may modulate inflammatory reactions within the CNS and could represent useful therapeutic targets in neuroinflammatory and neurodegenerative diseases.

**Figure 1:**
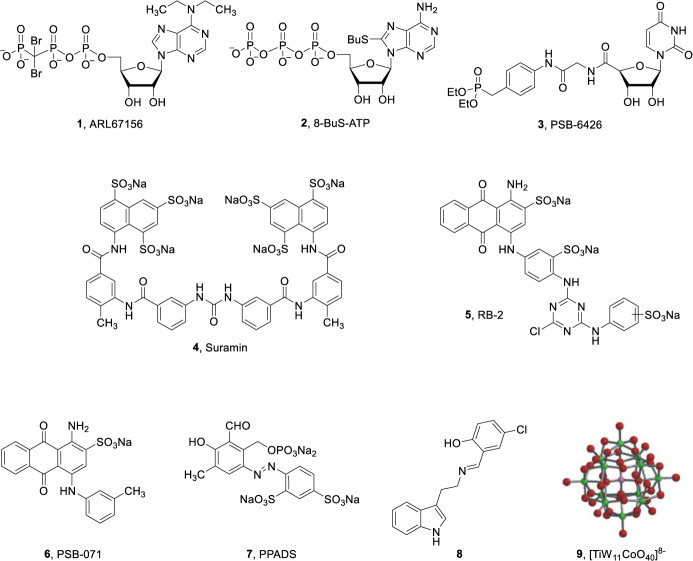
Structures of selected NTPDase inhibitors (Iqbal et al., 2005; Müller et al., 2006; Brunschweiger et al., 2008; Baqi et al., 2009b; Zebisch et al., 2014; Kanwal et al., 2019).

So far only few, moderately potent, NTPDase inhibitors have been described (**Figure 1**), which can be divided into nucleotide derivatives and non-nucleotides. ARL67156 (1, **Figure 1**) is a weak, competitive inhibitor of human NTPDase1 (*K*_i_ = 11 μM) and -3 (*K*_i_ = 18 μM) but does not inhibit human NTPDase2 and -8 (Lévesque et al., 2007). 8-BuS-ATP (2, **Figure 1**) was shown to inhibit NTPDase1 (*K*_i_ = 0.8 μM) but being a substrate of NTPDase2, -3, and -8 it is of limited use (Lecka et al., 2013). The corresponding 8-BuS-ADP and especially 8-BuS-AMP also inhibited NTPDase1 but appeared to be more stable towards hydrolysis (Lévesque et al., 2007). PSB-6426 (3, **Figure 1**) is a metabolically stable, uncharged compound derived from uridine-5′-carboxylate. It was identified as a moderately potent, selective competitive inhibitor of human NTPDase2 (*K*_i_ = 8.2 μM) (Brunschweiger et al., 2008). Non-nucleotide–derived compounds have also been developed as NTPDase inhibitors (compounds 4–7, **Figure 1**). These include suramin (4), reactive blue-2 (RB-2, 5), and its derivative PSB-071 (6), PPADS (**7**), and tryptamine-derived imine 8 (Iqbal et al., 2005; Baqi et al., 2009b; Zebisch et al., 2014; Kanwal et al., 2019). However, these compounds are non-selective and showed only moderate inhibitory activity in the low micromolar range and/or limited stability (Iqbal et al., 2005; Baqi et al., 2009b; Zebisch et al., 2014; Kanwal et al., 2019). Another class of NTPDase inhibitors are the polyoxometalates (POMs) such as [TiW_11_CoO_40_]^8-^ (9), which are inorganic, negatively charged metal complexes. POM derivative 9 inhibited rat NTPDase1, -2, and -3 in the submicromolar concentration range, but this highly negatively charged compound displays limited stability (Müller et al., 2006). Moreover, specific antibodies have been reported that inhibit NTPDase2 and -3 activities; however, the inhibition is not complete (Munkonda et al., 2009; Pelletier et al., 2017). Previously, we evaluated anthraquinone derivatives at rat NTPDase1, -2, and -3, and one of the most potent but non-selective compounds was PSB-071 (6) (Baqi et al., 2009b). In the present study, we investigated the structure-activity relationships (SARs) of this class of NTPDase inhibitors with the goal to improve their inhibitory potency and subtype-selectivity, in particular with the aim to obtain potent NTPDase2- (and NTPDase3-) selective inhibitors. Such compounds are required for biological studies since they are expected to lead to an accumulation of ADP thereby acting as indirect P2Y_1_, P2Y_12_ and P2Y_13_ receptor agonists.

## Experimental Section

### Chemistry

#### Material and Methods

All materials were used as purchased (Acros, Alfa Aesar, Merck, or Sigma-Aldrich, Germany). Thin-layer chromatography was performed using TLC aluminum sheets silica gel 60 F_254_ or TLC aluminum sheets reversed phase (RP) silica gel 18 F_254_ (Merck, Darmstadt, Germany). Colored compounds were visible at daylight; other compounds were visualized under UV light (254 nm). Flash chromatography was performed on a Büchi system using silica gel RP-18 (Merck, Darmstadt, Germany). ^1^H and ^13^C NMR data were collected on either a Bruker Avance 500 MHz NMR spectrometer at 500 MHz (^1^H) or 126 MHz (^13^C), respectively or a 600 MHz NMR spectrometer at 600 MHz (^1^H) or 151 MHz (^13^C), respectively. Deuterated dimethyl sulfoxide (DMSO-*d*_6_) or chloroform-*d* (CDCl_3_) were used as a solvent. Chemical shifts are reported in parts per million (ppm) relative to the deuterated solvent, i.e., DMSO, *δ* ^1^H 2.49 ppm; ^13^C 39.7 ppm, coupling constants *J* are given in Hertz, and spin multiplicities are given as s (singlet), d (doublet), t (triplet), q (quartet), sext (sextet), m (multiplet), and br (broad).

The purities of isolated products were determined by high performance liquid chromatography (HPLC) coupled with electrospray ionization mass spectrometry (ESI-MS) and ultraviolet (UV) detector using the following procedure: the compounds were dissolved at a concentration of 0.5 mg/mL in H_2_O/MeOH = 1:1, containing 2 mM NH_4_CH_3_COO. Then, 10 μL of the sample was injected into an HPLC column (Phenomenex Luna 3 μ C18, 50 mm × 2.00 mm). Elution was performed with a gradient of water:methanol (containing 2 mM NH_4_CH_3_COO) from 90:10 to 0:100 starting the gradient immediately at a flow rate of 250 μL/min for 15 min, followed by washing with 100% methanol for another 15 min. The purity of the compounds proved to be ≥95%. For microwave reactions, a CEM Focused Microwave Synthesis Type Discover apparatus was employed. A freeze-dryer (CHRIST ALPHA 1-4 LSC) was used for lyophilization.

The synthesis and analysis of compounds 11−22, 24−26, 31−33, 36, 38−40, 42−44, 46, 49−52, 54−56, and 58 was previously described (Baqi and Müller, 2007; Weyler et al., 2008; Baqi et al., 2009b; Baqi et al., 2010; Baqi and Müller, 2010; Baqi et al., 2011; Baqi and Müller, 2012; Fiene et al., 2016; Malik et al., 2016). All other compounds (23, 27−30, 34, 35, 37, 41, 45, 47, 48, 53, and 57) were newly prepared in analogy to described methods (Baqi and Müller, 2010; Baqi and Müller, 2012; Malik et al., 2016; Pelletier et al., 2017) with modifications as described below.

##### General Procedure A: Preparation of 4-Substituted 1-Aminoanthraquinone-2-sulfonate Derivatives (11-51)

**Scheme 1:**
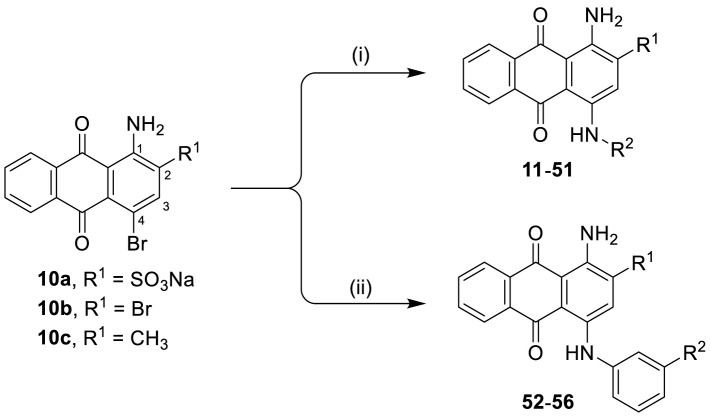
General synthesis of 4-substituted anthraquinone derivatives 11−56^a^. ^a^Reagents and conditions: (i) R^2^-NH_2_, phosphate buffer (pH 6−7), Cu^0^, microwave, 80−120°C, 5−24 min; (ii) *m*-substituted aniline, CuOAc, KOAc, 110°C, argon, 2–15 h; for R^1^ and R^2^, see **Table 1**.

To a 5 mL microwave reaction vial, equipped with a magnetic stirring bar, were added 1-amino-4-bromo substituted anthraquinone compounds [bromaminic acid sodium salt (10a) or 1-amino-2,4-dibromoanthraquinone (10b)] (0.1−0.3 mmol) and the appropriate aniline or amine derivative (1.5−9.0 equiv), followed by a buffer solution of Na_2_HPO_4_ (pH 9.6) (5.0 mL) and NaH_2_PO_4_ (pH 4.2) (1.0 mL) and a finely powdered elemental copper (0.002−0.003 g, 5−10 mol%). The mixture was capped and irradiated in the microwave oven (80−100 W) for 5−24 min at 100−120°C. The reaction mixture was cooled down to room temperature (rt), and the product was purified using the following procedure. The contents of the vial were filtered to remove the elemental copper. Then, ca. 200 mL of water was added to the filtrate, and the aqueous solution was extracted with dichloromethane (200 mL). The extraction procedure was repeated until the dichloromethane layer became colorless (two to three times). The aqueous layer was reduced by rotary evaporation to a volume of 10−20 mL, which was subsequently submitted to flash column chromatography using RP-18 silica gel and water as an eluent. The polarity of the eluent was then gradually decreased by the addition of acetone in the following steps: 5, 10, 20, 40, and 60%. Fractions containing blue product were collected. For some compounds the last step of purification (RP-18 flash chromatography) had to be repeated two to three times to obtain pure product (≥95% purity as determined by HPLC-UV-MS). The pooled product-containing fractions were evaporated under vacuum to remove the acetone and reduce the water volume. The remaining water was subsequently removed by lyophilization to yield up to 80% of the product as blue powder (**Scheme 1** and **Table 1**).

**Table 1:** Inhibitory activity of anthraquinone derivatives at human ecto-NTPDases.

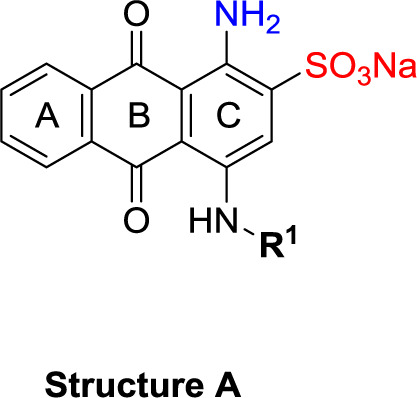		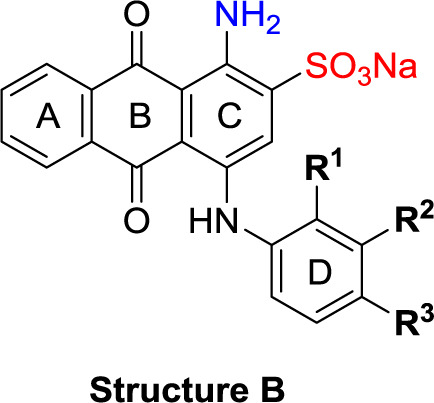		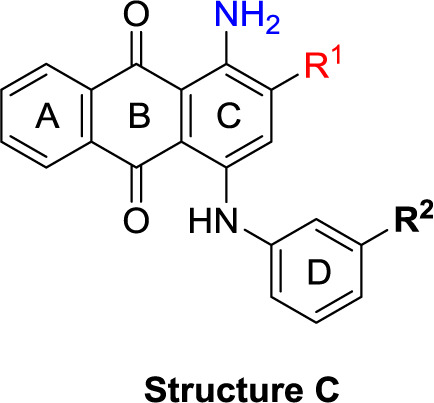		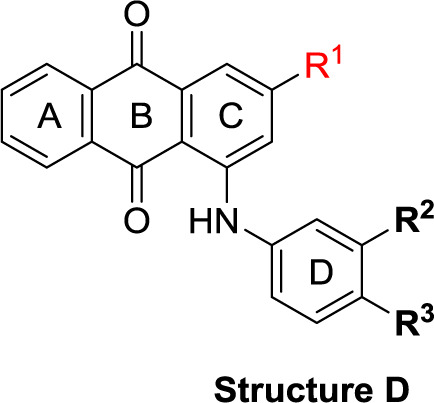	
				IC_50_ ± SEM (μM)^a^ (or % inhibition at 2 μM concentration)			
				NTPDase1	NTPDase2	NTPDase3	NTPDase8
Compd.	R^1^	R^2^	R^3^				
**5** RB-2		For structure see **Figure 1**		(17)	(42)	**0.942** ± 0.024	(-8)
**6** PSB-071		For structure see **Figure 1**		(-4) 51.5 (rat)^b^	(22) 12.8 (rat)^b^	(1) 19.1 (rat)^b^	(-21)
**Structure A**							
**11**	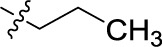	–	–	(-3)	(0)	(6)	(-4)
**12**	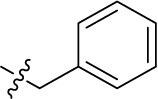	–	–	(-10)	(11)	(6)	(3)
**13**	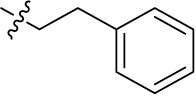	–	–	(-12)	(12)	(-3)	(-3)
**14**	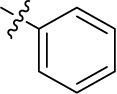	–	–	(-13)	(15)	(-10)	(-15)
**15**	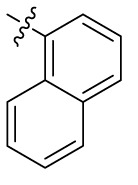	–	–	(-19) >100 (rat)^b^	(31) >100 (rat)^b^	(-4) 1.5 (rat)_b_	(9)
**16**	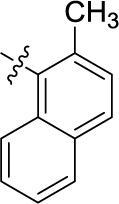	–	–	(3)	**5.62** ± 0.72	(-21)	(3)
**17**	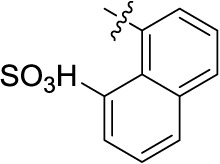	–	–	(-1)	(15)	**1.64** ± 0.26	(-11)
**18**	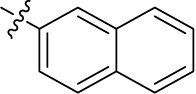	–	–	(-8) 0.328 (rat)^b^	(34) 19.1 (rat)^b^	(-9) 2.22 (rat)^b^	(3)
**19**	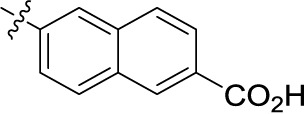	–	–	(0)	(13)	**4.72** ± 0.40	(-35)
**20**PSB-16131	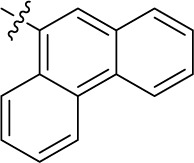	–	–	(-7)	**0.539** ± 0.290	(-5)	(6)
**Structure B**							
**21**	H	F	H	(-8)	(15)	(0)	(-1)
**22**	H	Br	H	(-4)	(12)	**8.96** ± 1.08	(-26)
**23**	H	I	H	(0)	(21)	(2)	(4)
**24**	H	NO_2_	H	(-32)	(19)	**15.3** ± 2.5	(3)
**25**	H	H	NH_2_	(-19)	(17)	(-25)	(-12)
**26**	H	CO_2_H	H	(-17)	(2)	**3.10** ± 0.45	(-17)
**27**	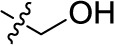	H	H	(-1)	(12)	(11)	(4)
**28**	H	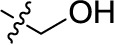	H	(-2)	(7)	(-9)	(1)
**29**	H	H	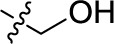	(-4)	(5)	(-3)	(-4)
**30**	H	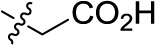	H	(-6)	(7)	**1.52** ± 0.28	(-11)
**31**	H	NH_2_	SO_3_H	(-15)	(1)	**4.26** ± 0.54	(-19)
**32**	H	SO_3_H	NH_2_	(10)	(5)	**1.73** ± 0.55	(-20)
**33**PSB-2046	H	CO_2_H	OH	(-12)	(12)	**0.723** ± 0.032	(-9)
**34**	Cl	Cl	H	(1)	(16)	(-2)	(7)
**35**	CO_2_H	F	H	(-21)	(-3)	(32)	(-10)
**36**	CO_2_H	H	Cl	(-20)	(-2)	**4.04** ± 0.47	(-17)
**37**	F	H	OH	(-8)	(5)	(-12)	(-6)
**38**	CH_3_	H	Cl	(2)	**5.45** ± 0.70	(9)	(-1)
**39**	H	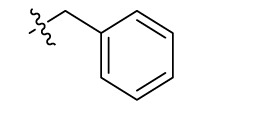	H	(36)	**3.59** ± 0.85	**13.1** ± 1.65	(28)
**40**	H	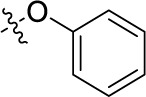	H	(12)	**1.11** ± 0.06	(18)	(9)
**41**	H	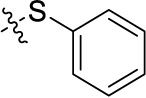	H	(1)	**0.984** ± 0.327	(10)	(6)
**42**PSB-1011	H	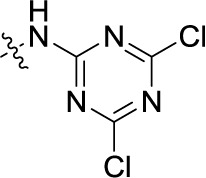	SO_3_H	(-2)	(10)	**0.390** ± 0.041	(-7)
**43**	H	H	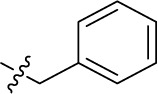	(7)	**1.32** ± 0.05	(3)	(3)
**44**	H	H	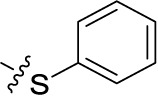	(15)	**0.934** ± 0.136	(7)	(6)
**45**	H	H	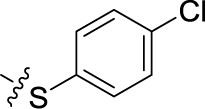	(52)	**1.08** ± 0.08	(42)	(21)
**46**	H	H	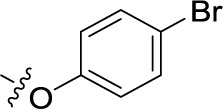	(33)	**1.73** ± 0.29	(21)	(10)
**47**	H	H	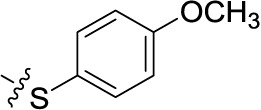	(34)	**0.951** ± 0.225	(16)	(4)
**48**PSB-2020	H	Cl	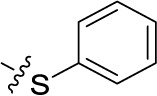	(29)	**0.551** ± 0.195	(20)	(14)
**49**	H	H	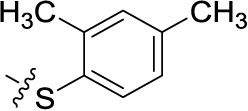	(46)	**1.28** ± 0.34	(36)	(27)
**50**	H	H	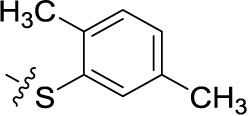	(38)	**1.13** ± 0.27	(34)	(17)
**51**	H	H	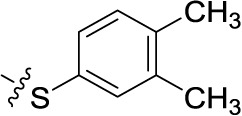	(51)	**0.832** ± 0.053	(44)	(27)
**Structure C**							
**52**	Br	CO_2_H	–	(-2)	(-5)	(1)	(3)
**53**	Br	C_2_H_5_	–	(-4)	(5)	(-2)	(-4)
**54**	CH_3_	F	–	(-4)	(7)	(-2)	(-2)
**55**	CH_3_	OCH_3_	–	(-6)	(3)	(0)	(-2)
**56**	CH_3_	C_2_H_5_	–	(-3)	(6)	(-1)	(-3)
**Structure D**							
**57**	SO_3_H	F	H	(14)	(-6)	(9)	(-1)
**58**	SO_3_H	CO_2_H	OH	(1)	(5)	(32)	(5)

^a^IC_50_ values for potent inhibitors are highlighted in bold. ^b^Baqi et al., 2009b. Data are from at least three separate experiments.

##### General Procedure B: Preparation of 2-Substituted 1-Amino-4-anilinoanthraquinone Derivatives (52-56)

A round bottom flask (25 mL) equipped with a magnetic stirring bar was charged with one equivalent of starting material (10b or 1-amino-4-bromo-2-methylanthraquinone (10c)), an excess of appropriate aniline derivative (15 equiv.) and copper(I) acetate (10 mol%) in the presence of 2.25 equiv. of potassium acetate (**Scheme 1**). The resulting mixture was heated at 110°C under an argon atmosphere for 2−15 h, and the progress of the reaction was monitored by TLC using 10% dichloromethane/cyclohexane as eluent. The reaction mixture was then let to cool down to room temperature, followed by the addition of ethanol (5 mL), and the blue-colored precipitate was filtered off and washed successively with ethanol, 0.1 M HCl, and water (ca. 15 mL each), and then the solid material was dried at 70°C in the oven for 16 h. The product was then purified by silica gel column chromatography using dichloromethane/cyclohexane (9:1) as eluent. The desired products (52−56) were obtained in high yields (**Scheme 1** and **Table 1**).

##### General Procedure C: Preparation of 4-Substituted Anthraquinone-2-sulfonate Derivatives (57 and 58)

**Scheme 2:**
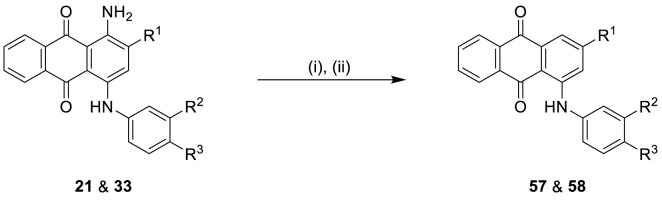
Synthesis of deaminated anilinoanthraquinone derivatives 57 and 58^a^. ^a^Reagents and conditions: (i) NaNO_2_, HCl (1 M), 0−5°C, 5 min; (ii) Zn (10 equiv.), ethanol, rt, 30 s; for R^1^, R^2^, and R^3^, see **Table 1**.

To a 50 mL round bottom flask equipped with a magnetic stirring bar, 0.1 mmol of 1-aminoanthraquinone derivative (21 or 33) was added, followed by 5 mL of 1 M hydrochloric acid. The solution was cooled to 0−5°C in an ice bath, and a previously cooled solution of NaNO_2_ (13.8 mg, 0.2 mmol, 2 equiv) in 0.5 mL of distilled water was added dropwise. After 5 min, the mixture was allowed to warm up to rt, followed by addition of 30 mg of zinc powder (1.0 mmol, 10 equiv) and 5 mL of ethanol. The resulting mixture was then allowed to stir at rt for ca. 30 s. The mixture was filtered off, and the purple-colored filtrate was then purified by flash column chromatography on a reversed phase silica gel (RP-18) using a gradient of acetone in water (5 and 20%) as the eluent. Fractions containing the purple product were collected and evaporated in vacuum to remove acetone and decrease the volume of water to ca. 10-20 mL. Complete drying was achieved with a freeze-dryer, affording purple-colored products in excellent yields (**Scheme 2** and **Table 1**).

##### Sodium 1-amino-4-(3-iodophenylamino)-9,10-dioxo-9,10-dihydroanthracene-2-sulfonate (23)

Reaction conditions according to general procedure A: Compound 10a (0.1213 g, 0.3 mmol), 3-iodoaniline (0.1314 g, 0.6 mmol), a buffer solution of Na_2_HPO_4_ (pH 9.6) (5.0 mL) and NaH_2_PO_4_ (pH 4.2) (1.0 mL), and copper metal (0.003–0.005 g, 0.05–0.08 mmol). MW conditions: 100 W, 120°C, 10 min. Analytical data: blue powder (34% yield), mp >300°C. ^1^H NMR (500 MHz, DMSO-*d*_6_): δ 7.20 (t, *J* = 7.9 Hz, 1H, 2′-H), 7.29 (m, 1H, 5′-H or 6′-H), 7.51 (m, 1H, 5′-H or 6′-H), 7.65 (t, *J* = 1.9 Hz, 1H, 4′-H), 7.85 (m, 2H, 6-H, 7-H), 7.98 (s, 1H, 3-H), 8.25 (m, 2H, 5-H, 8-H), 11.79 (s, 1H, 4-NH). ^13^C NMR (126 MHz, DMSO-*d*_6_): δ 95.55, 109.51, 112.66, 121.91, 122.99, 126.12, 126.20, 130.91, 131.53, 132.70, 133.00, 133.51, 133.58, 134.26, 139.56, 141.38, 142.66, 144.66, 182.12, 183.07. LC-MS (*m/z*): 519.2 [M – Na]^–^, 521.4 [M – Na]^+^. Purity by HPLC-UV (254 nm)-ESI-MS: 100%.

##### Sodium 1-amino-4-(2-(hydroxymethyl)phenylamino)-9,10-dioxo-9,10-dihydroanthracene-2-sulfonate (27)

Reaction conditions according to general procedure A: Compound 10a (0.1213 g, 0.3 mmol), 2-aminobenzyl alcohol (0.0738 g, 0.6 mmol), a buffer solution of Na_2_HPO_4_ (pH 9.6) (5.0 mL) and NaH_2_PO_4_ (pH 4.2) (1.0 mL), and copper metal (0.003–0.005 g, 0.05–0.08 mmol). MW conditions: 100 W, 120°C, 10 min. Analytical data: blue powder (44.3% yield), mp >300°C. ^1^H NMR (500 MHz, DMSO-*d*_6_): δ 4.52 (d, *J* = 4.1 Hz, 2H, -C*H_2_*OH), 5.30 (t, *J* = 5.0 Hz, 1H, -CH_2_O*H*), 7.23 (m, 3H, 3′-H, 5′-H, 6′-H), 7.34 (td, *J* = 7.7, 1.6 Hz, 1H, 4′-H), 7.50 (dd, *J* = 7.6, 1.5 Hz, 1H, 3-H), 7.84 (m, 2H, 6-H, 7-H), 8.26 (m, 2H, 5-H, 8-H), 11.94 (s, 1H, 4-NH). ^13^C NMR (126 MHz, DMSO-*d*_6_): δ 60.59, 109.31, 111.72, 123.37, 123.96, 124.69, 126.07, 126.15, 128.10, 129.15, 132.85, 133.19, 133.80, 134.31, 135.79, 137.78, 141.26, 142.65, 144.41, 181.95, 182.34. LCMS (*m*/*z*): 423.1 [M – Na]^–^. Purity by HPLC-UV(220–700 nm)-ESI-MS 100%.

##### Sodium 1-amino-4-(3-(hydroxymethyl)phenylamino)-9,10-dioxo-9,10-dihydroanthracene-2-sulfonate (28)

Reaction conditions according to general procedure A: Compound 10a (0.1213 g, 0.3 mmol), 3-aminobenzyl alcohol (0.0738 g, 0.6 mmol), a buffer solution of Na_2_HPO_4_ (pH 9.6) (5.0 mL) and NaH_2_PO_4_ (pH 4.2) (1.0 mL), and copper metal (0.003–0.005 g, 0.05–0.08 mmol). MW conditions: 100 W, 120°C, 10 min. Analytical data: blue powder (67.7% yield), mp >300°C. ^1^H NMR (500 MHz, DMSO-*d*_6_): δ 4.52 (d, *J* = 3.7 Hz, 2H, –C*H_2_*OH), 5.24 (t, *J* = 5.7 Hz, 1H, –CH_2_O*H*), 7.15 (m, 2H, 5′-H, 6′-H), 7.21 (s, 1H, 2′-H), 7.39 (t, *J* = 7.7 Hz, 1H, 3′-H), 7.85 (m, 2H, 6-H, 7-H), 7.99 (s, 1H, 3-H), 8.27 (m, 2H, 5-H, 8-H), 12.06 (s, 1H, 4-NH). ^13^C NMR (126 MHz, DMSO-*d*_6_): δ 62.73, 109.23, 111.39, 121.37, 121.55, 122.74, 122.92, 126.08, 126.16, 129.46, 132.88, 133.27, 133.73, 134.29, 139.19, 141.21, 142.97, 144.46, 144.71, 181.90, 182.53. LCMS (*m*/*z*): 423.1 [M – Na]^–^. Purity by HPLC-UV(220–700 nm)-ESI-MS 100%.

##### Sodium 1-amino-4-(4-(hydroxymethyl)phenylamino)-9,10-dioxo-9,10-dihydroanthracene-2-sulfonate (29)

Reaction conditions according to general procedure A: Compound 10a (0.1213 g, 0.3 mmol), 4-aminobenzyl alcohol (0.0738 g, 0.6 mmol), a buffer solution of Na_2_HPO_4_ (pH 9.6) (5.0 mL) and NaH_2_PO_4_ (pH 4.2) (1.0 mL), and copper metal (0.003–0.005 g, 0.05–0.08 mmol). MW conditions: 100 W, 120°C, 10 min. Analytical data: blue powder (50.9% yield), mp >300°C. ^1^H NMR (500 MHz, DMSO-*d*_6_): δ 4.51 (d, *J* = 5.2 Hz, 2H, –C*H_2_*OH), 5.19 (t, *J* = 5.8 Hz, 1H, –CH_2_O*H*), 7.24 (m, 2H, 3′-H, 5′-H), 7.39 (m, 2H, 2′-H, 6′-H), 7.84 (m, 2H, 6-H, 7-H), 7.98 (s, 1H, 3-H), 8.27 (m, 2H, 5-H, 8-H), 12.06 (s, 1H, 4-NH). ^13^C NMR (126 MHz, DMSO-*d*_6_): δ 62.73, 109.21, 111.20, 122.78, 123.34, 126.06, 126.16, 128.03, 132.87, 133.23, 133.75, 134.29, 137.77, 139.24, 141.43, 143.00, 144.43, 181.87, 182.40. LCMS (*m*/*z*): 423.1 [M – Na]^–^. Purity by HPLC-UV(220–900 nm)-ESI-MS 98%.

##### Sodium 1-amino-4-(3-(carboxymethyl)phenylamino)-9,10-dioxo-9,10-dihydroanthracene-2-sulfonate (30)

Reaction conditions according to general procedure A: Compound 10a (0.1213 g, 0.3 mmol), 3-aminophenylacetic acid (0.0906 g, 0.6 mmol), a buffer solution of Na_2_HPO_4_ (pH 9.6) (5.0 mL) and NaH_2_PO_4_ (pH 4.2) (1.0 mL), copper metal (0.003–0.005 g, and 0.05–0.08 mmol). MW conditions: 100 W, 120°C, 10 min. Analytical data: blue powder (73% yield), mp >300°C. ^1^H NMR (500 MHz, DMSO-*d*_6_): δ 3.40 (s, 2H, *-CH_2_*CO_2_H), 7.07 (m, 2H, 4′-H, 6′-H), 7.18 (s, 1H, 2′-H), 7.31 (t, *J* = 7.8 Hz, 1H, 5′-H), 7.83 (m, 2H, 6-H, 7-H), 8.02 (s, 1H, 3-H), 8.27 (m, 2H, 5-H, 8-H), 10.11 (s, 1H, -CH_2_*CO_2_H*), 12.08 (s, 1H, 4-NH). ^13^C NMR (126 MHz, DMSO-*d*_6_): δ 43.74, 109.23, 111.29, 120.49, 122.96, 124.25, 125.76, 126.09, 126.16, 129.11, 132.87, 133.24, 133.76, 134.28, 138.84, 141.24, 142.93, 144.44, 150.43, 173.43, 181.89, 182.46. LCMS (*m*/*z*): 451.4 [M – Na]^–^, 453.3 [M – Na]^+^. Purity by HPLC-UV(220–700 nm)-ESI-MS 100%.

##### Sodium 1-amino-4-(2,3-dichlorophenylamino)-9,10-dioxo-9,10-dihydroanthracene-2-sulfonate (34)

Reaction conditions according to general procedure A: Compound 10a (0.3639 g, 0.9 mmol), 2,3-dichloroaniline (0.4374 g, 2.7 mmol), a buffer solution of Na_2_HPO_4_ (pH 9.6) (5.0 mL) and NaH_2_PO_4_ (pH 4.2) (1.0 mL), and copper metal (0.003–0.005 g, 0.05–0.08 mmol). MW conditions: 100 W, 120°C, 10 min. Analytical data: blue powder (20% yield), mp >300°C. ^1^H NMR (500 MHz, DMSO-*d*_6_): δ 7.40 (m, 3H, 4′-H, 5′-H, 6′-H), 7.86 (m, 2H, 6-H, 7-H), 7.93 (s, 1H, 3-H), 8.27 (m, 2H, 5-H, 8-H), 11.90 (s, 1H, 4-NH). ^13^C NMR (126 MHz, DMSO-*d*_6_): δ 109.75, 113.86, 121.13, 123.01, 124.14, 125.11, 126.22, 126.25, 128.64, 132.84, 133.10, 133.40, 133.79, 134.26, 138.08, 139.08, 142.39, 144.85, 182.30, 183.86. LCMS (*m*/*z*): 461.1 [M – Na]^–^, 463.1 [M – Na]^+^. Purity by HPLC-UV(220–700 nm)-ESI-MS 100%.

##### Sodium 1-amino-4-(2-carboxy-3-fluorophenylamino)-9,10-dioxo-9,10-dihydroanthracene-2-sulfonate (35)

Reaction conditions according to general procedure A: Compound 10a (0.1213 g, 0.3 mmol), 2-amino-6-fluorobenzoic acid (0.0930 g, 0.6 mmol), a buffer solution of Na_2_HPO_4_ (pH 9.6) (5.0 mL) and NaH_2_PO_4_ (pH 4.2) (1.0 mL), and copper metal (0.003–0.005 g, 0.05–0.08 mmol). MW conditions: 100 W, 120°C, 10 min. Analytical data: blue powder (40.1% yield), mp >300°C. ^1^H NMR (500 MHz, DMSO-*d*_6_): δ 6.78 (t, *J* = 8.6 Hz, 1H, 6′-H), 6.94 (d, *J* = 8.0 Hz, 1H, 4′-H), 7.15 (m, 1H, 5′-H), 7.81 (m, 2H, 6-H, 7-H), 8.10 (s, 1H, 3-H), 8.26 (m, 2H, 5-H, 8-H), 12.07 (s, 1H, 4-NH). ^13^C NMR (126 MHz, DMSO-*d*_6_): δ 109.66, 109.89, 110.08, 113.17, 116.81, 124.44, 126.03, 126.25, 127.21, 132.80, 133.11, 133.84, 134.20, 138.60, 139.02, 141.95, 144.57, 158.56, 160.49, 166.00, 181.84, 182.19. LCMS (*m*/*z*): 455.2 [M – Na]^–^, 457.3 [M – Na]^+^. Purity by HPLC-UV(220–700 nm)-ESI-MS 98%.

##### Sodium 1-amino-4-(2-fluoro-4-hydroxyphenylamino)-9,10-dioxo-9,10-dihydroanthracene-2-sulfonate (37)

Reaction conditions according to general procedure A: Compound 10a (0.1213 g, 0.3 mmol), 4-amino-3-fluorophenol (0.0762 g, 0.6 mmol), a buffer solution of Na_2_HPO_4_ (pH 9.6) (5.0 mL) and NaH_2_PO_4_ (pH 4.2) (1.0 mL), and copper metal (0.003–0.005 g, 0.05–0.08 mmol). MW conditions: 100 W, 120°C, 10 min. Analytical data: blue powder (38.4% yield), mp >300°C. ^1^H NMR (500 MHz, DMSO-*d*_6_): δ 6.72 (m, 2H, 4′-H, 5′-H), 7.23 (s, 1H, 3′-H), 7.58 (d, *J* = 1.7 Hz, 1H, 3-H), 7.84 (m, 2H, 6-H, 7-H), 8.27 (m, 2H, 5-H, 8-H), 11.68 (s, 1H, 4-NH). ^13^C NMR (126 MHz, DMSO-*d*_6_): δ 103.65, 103.82, 110.51, 112.18, 117.38, 122.18, 126.06, 126.16, 128.64, 132.85, 133.21, 133.71, 134.30, 142.94, 143.25, 144.12, 157.03, 158.34, 181.86, 182.50. LCMS (*m*/*z*): 427.3 [M – Na]^–^, 429.2 [M – Na]^+^. Purity by HPLC-UV(220–900 nm)-ESI-MS 97%.

##### Sodium 1-amino-4-(3-(phenylsulfanyl)phenylamino)-9,10-dioxo-9,10-dihydroanthracene-2-sulfonate (41)

Reaction conditions according to general procedure A: Compound 10a (0.1213 g, 0.3 mmol), 3-(phenylsulfanyl)aniline (0.0664 g, 0.33 mmol), a buffer solution of Na_2_HPO_4_ (pH 9.6) (5.0 mL) and NaH_2_PO_4_ (pH 4.2) (1.0 mL), and copper metal (0.003–0.005 g, 0.05–0.08 mmol). MW conditions: 100 W, 120°C, 7 min. Analytical data: blue powder (14.7% yield), mp >300°C. ^1^H NMR (500 MHz, DMSO-*d*_6_): δ 7.07 (m, 1H, 6′-H), 7.12 (t, *J* = 1.9 Hz, 1H, 2′-H), 7.18 (dd, *J* = 7.8, 1.9 Hz, 1H, 4′-H), 7.34 (m, 1H, 5′-H), 7.45 (m, 5H, 2″-H, 3″-H, 4″-H, 5″-H, 6″-H), 7.85 (m, 2H, 6-H, 7- H), 8.01 (s, 1H, 3-H), 8.25 (m, 2H, 5-H, 8-H), 11.84 (s, 1H, 4-NH). ^13^C NMR (126 MHz, DMSO-*d*_6_): δ 109.24, 112.04, 121.11, 122.70, 123.10, 125.00, 125.92, 126.00, 127.94, 129.79, 130.59, 131.82, 132.78, 133.13, 133.25, 133.43, 134.08, 137.12, 139.86, 140.44, 142.58, 144.41, 181.88, 182.72. LCMS (*m*/*z*): 501.0 [M – Na]^–^, 503.2 [M – Na]^+^. Purity by HPLC-UV(220–700 nm)-ESI-MS 99.4%.

##### Sodium 1-amino-4-[4-(4-chlorophenylthio)phenylamino]-9,10-dioxo-9,10-dihydroanthracene-2-sulfonate (45)

Reaction conditions according to general procedure A: Compound 10a (0.1213 g, 0.3 mmol), 4-(4-chlorophenylsulfanyl)aniline (0.0778 g, 0.33 mmol), a buffer solution of Na_2_HPO_4_ (pH 9.6) (5.0 mL) and NaH_2_PO_4_ (pH 4.2) (1.0 mL), and copper metal (0.003–0.005 g, 0.05–0.08 mmol). MW conditions: 100 W, 120°C, 8 min. Analytical data: blue powder (6% yield), mp >300°C. ^1^H NMR (500 MHz, DMSO-*d*_6_): δ 7.30 (m, 2H, 2′-H, 6′-H), 7.32 (m, 2H, 3″-H, 5″-H), 7.42 (m, 2H, 3′-H, 5′-H), 7.46 (m, 2H, 2″-H, 6″-H), 7.86 (m, 2H, 6-H, 7-H), 8.07 (s, 1H, 3-H), 8.27 (m, 2H, 5-H, 8-H), 11.91 (s, 1H, 4-NH). ^13^C NMR (126 MHz, DMSO-*d*_6_): δ 109.40, 112.62, 123.00, 125.96, 126.03, 127.22, 129.36, 130.82, 131.44, 132.84, 133.34, 133.42, 133.83, 134.08, 135.46, 139.19, 139.95, 142.45, 144.55, 181.96, 182.92. LCMS (*m*/*z*): 535.0 [M – Na]^–^, 536.1 [M – Na]^+^. Purity by HPLC-UV(220–400 nm)-ESI-MS 99%.

##### Sodium 1-amino-4-[4-(4-methoxyphenylthio)phenylamino]-9,10-dioxo-9,10-dihydroanthracene-2-sulfonate (47)

Reaction conditions according to general procedure A: Compound 10a (0.1213 g, 0.3 mmol), 4-(4-methoxyphenylsulfanyl)aniline (0.0763 g, 0.33 mmol), a buffer solution of Na_2_HPO_4_ (pH 9.6) (5.0 mL) and NaH_2_PO_4_ (pH 4.2) (1.0 mL), and copper metal (0.003–0.005 g, 0.05–0.08 mmol). MW conditions: 100 W, 120°C, 10 min. Analytical data: blue powder (14.2% yield), mp >300°C. ^1^H NMR (500 MHz, DMSO-*d*_6_): δ 3.79 (s, 3H, –OCH_3_), 7.01 (m, 2H, 2′-H, 6′-H), 7.23 (s, 4H, 2″-H, 3″-H, 5″-H, 6″-H), 7.44 (m, 2H, 3′-H, 5′-H), 7.85 (m, 2H, 6-H, 7-H), 7.99 (s, 1H, 3-H), 8.26 (m, 2H, 5-H, 8-H), 11.95 (s, 1H, 4-NH). ^13^C NMR (126 MHz, DMSO-*d*_6_): δ 55.26, 109.21, 111.78, 115.36, 122.71, 123.54, 123.77, 125.92, 126.01, 129.91, 132.43, 132.77, 133.20, 133.48, 134.08, 134.70, 137.83, 140.16, 142.62, 144.39, 159.55, 181.82, 182.55. LCMS (*m*/*z*): 531.1 [M – Na]^–^, 532.2 [M – Na]^+^. Purity by HPLC-UV(220–400 nm)-ESI-MS 98.9%.

##### Sodium 1-amino-4-(3-chloro-4-phenylsulfanyl)-phenylamino-9,10-dioxo-9,10-dihydroanthracene-2-sulfonate (48)

Reaction conditions according to general procedure A: Compound 10a (0.1213 g, 0.3 mmol), 3-chloro-4-(phenylsulfanyl)aniline (0.0778 g, 0.33 mmol), a buffer solution of Na_2_HPO_4_ (pH 9.6) (5.0 mL) and NaH_2_PO_4_ (pH 4.2) (1.0 mL), and copper metal (0.003–0.005 g, 0.05–0.08 mmol). MW conditions: 100 W, 120°C, 8 min. Analytical data: blue powder (6.5% yield), mp >300°C. ^1^H NMR (500 MHz, DMSO-*d*_6_): δ 7.21 (dd, *J* = 8.5, 2.3 Hz, 1H, 6′-H), 7.25 (d, *J* = 8.5 Hz, 1H, 5′-H), 7.34 (m, 3H, 3″-H, 4″-H, 5″-H), 7.42 (m, 2H, 2″-H, 6″-H), 7.51 (d, *J* = 2.2 Hz, 1H, 2′-H), 7.86 (m, 2H, 6-H, 7-H), 8.03 (s, 1H, 3-H), 8.25 (m, 2H, 5-H, 8-H), 11.68 (s, 1H, 4-NH). ^13^C NMR (126 MHz, DMSO-*d*_6_): δ 109.60, 113.55, 121.02, 122.52, 123.29, 125.99, 126.05, 126.99, 127.60, 129.71, 130.51, 132.92, 133.33, 133.49, 133.64, 133.69, 134.06, 135.28, 138.17, 140.95, 142.21, 144.71, 182.12, 183.21. LCMS (*m*/*z*): 535.0 [M – Na]^–^, 536.1 [M – Na]^+^. Purity by HPLC-UV(220–700 nm)-ESI-MS 97.7%.

##### 1-Amino-2-bromo-4-(3-ethylphenylamino)anthracene-9,10-dione (53)

Reaction conditions according to general procedure B: Compound 10b (0.1143 mg, 0.3 mmol, 1 equiv.), 3-ethylaniline (0.5453 mg, 4.5 mmol, 15 equiv.), copper(I) acetate (0.0037 mg, 10 mol%) and potassium acetate (0.066 mg, 0.68 mmol) at 110°C for 2 h. Analytical data: dark blue powder (57.6% yield), mp = 229–230°C. ^1^H NMR (500 MHz, Chloroform-*d*): δ 1.29 (t, *J* = 7.6 Hz, 3H, –CH_2_-C*H_3_*), 2.68 (q, *J* = 7.6 Hz, 2H, –C*H_2_*-CH_3_), 7.05 (*d*, 1H, 4′-H), 7.10 (m, 2H, 2′-H, 6′-H), 7.33 (m, 1H, 5′-H), 7.76 (m, 2H, 6-H 7-H), 7.88 (s, 1H, 3-H), 8.35 (m, 2H, 5-H, 8-H), 11.90 (s, 1H, 4-NH). ^13^C NMR (126 MHz, Chloroform-*d*): δ 15.65, 28.94, 111.50, 111.73, 121.23, 122.93, 123.61, 124.79, 126.51, 126.76, 127.74, 129.68, 133.09, 133.15, 133.90, 134.45, 139.47, 142.81, 143.07, 146.32, 183.77, 183.88. LC-MS (*m/z*): 419.2 [M – H]^–^, 421.2 [M + H]^+^. Purity by HPLC-UV(254 nm)-ESI-MS 95%.

##### 4-(3-Fluorophenylamino)-9,10-dioxo-9,10-dihydroanthracene-2-sulfonic acid (57)

Reaction conditions according to general procedure C: Compound 21 (0.0434, 0.1 mmol) was dissolved in 5 mL of 1 M HCl then cooled down to 0–5°C in an ice bath. Subsequently NaNO_2_ (14 mg, 0.2 mmol) dissolved in water (0.5 mL) was added portion-wise, and the mixture was stirred for 5 min. It was then warmed up to rt followed by the addition of ethanol (5 mL) and zinc (65 mg, 1 mmol, 10 equiv.) and left stirring at rt for 30 s. Analytical data: dark violet powder (75% yield), mp >300°C. ^1^H NMR (600 MHz, DMSO-*d*_6_): δ 7.06 (td, *J* = 8.6, 2.5 Hz, 1H, 5′-H), 7.22 (m, 2H, 4′-H, 6′-H), 7.50 (m, 1H, 2′-H), 7.79, 7.85 (2 d, *J* = 1.5 Hz, each 1H, 1-H, 3-H), 7.89, 7.93 (2 td, *J* = 7.5, 1.5 Hz, each 1H, 6-H, 7-H), 8.19, 8.24 (2 dd, *J* = 7.7, 1.3 Hz, 1H, each 1H, 5-H, 8-H), 11.20 (s, 1H, 4-NH). ^13^C NMR (151 MHz, DMSO-*d*_6_): δ 110.34, 110.50, 111.64, 111.78, 114.12, 115.71, 116.23, 119.36, 119.38, 126.69, 126.84, 131.44, 131.51, 132.67, 134.16, 134.35, 134.47, 134.86, 141.12, 141.19, 147.90, 154.52, 162.16, 163.78, 182.48, 184.68. LCMS (*m*/*z*): 396.0 [M – H]^–^, 398.1 [M + H]^+^. Purity by HPLC-UV(220–800 nm)-ESI-MS 99%.

### Malachite Green Assay to Investigate NTPDase Inhibitors

Membrane preparations expressing human NTPDase1, -2, -3, or -8, respectively, were obtained as previously described (Sévigny et al., 1997; Cogan et al., 1999; Kukulski et al., 2005; Lecka et al., 2013; Lee et al., 2018). Enzyme inhibition assays were performed using the malachite green assay in analogy to published procedures with some modifications (Dou et al., 2018). The reaction buffer contained 10 mM HEPES, 2 mM CaCl_2_, and 1 mM MgCl_2_ (pH 7.4) in a final volume of 50 μL in transparent 96-well half-area plates. The compounds were initially tested at a final concentration of 2 µM using a COS-7-cell membrane preparation expressing the appropriate NTPDase isoenzyme (protein amount: 143 ng for NTPDase1, 175 ng for NTPDase2, 152 ng for NTPDase3, and 175 ng for NTPDase8). Preincubated of the enzyme preparations was perfomred at 37°C in the presence or absence of test compounds with gentle shaking (Eppendorf Thermomixer comfort at 500 rpm) for 5 min. The reaction was initiated by the addition of 50 µM ATP [*K_m_* (CD39) = 17 µM] for NTPDase1 or 100 µM ATP for NTPDase2, -3, and -8 [*K_m_* (NTPDase2) = 70 µM; *K_m_* (NTPDase3) = 75 µM; *K_m_* (NTPDase8) = 46 µM] (Kukulski et al., 2005). After 15 min of incubation at 37°C with gentle shaking, the reaction was stopped by the addition of the detection reagents (20 µL malachite green solution, 0.6 mM, and 30 µL of ammonium molybdate solution, 20 mM, in 1.5 M sulfuric acid). The released inorganic phosphate was quantified after 20 min of gentle shaking at 25°C by measuring the absorption of the malachite green-phosphomolybdate complex at 600 nm using a BMG PheraStar FS plate reader (BMG Labtech GmbH, Ortenberg, Germany). The corrected absorption was calculated by subtracting the absorption of the negative control samples, which were incubated with previously denatured enzyme (90°C, 15 min). Full concentration-inhibition curves were determined with inhibitor concentrations ranging from 0.03 to 30 µM in the presence of 2% DMSO. Inhibition-type experiments were performed with 25, 50, 100, 150 and 200 µM ATP as substrate for NTPDase2 in the presence of inhibitor 20 (0, 0.25, 0.5, and 1 µM) and 25, 50, 100 and 150 µM ATP substrate for NTPDase3 and compound 42 (0.25, 0.5, and 1 µM). For all of the presented data, at least three independent experiments were performed, and IC_50_ values were calculated by GraphPad Prism 8 software.

## Results and Discussion

A library of 48 anthraquinone derivatives was synthesized and tested at human NTPDase1, -2, -3, and -8, which are ecto-enzymes hydrolyzing extracellular nucleotides, using the malachite green assay. Subsequently, inhibition curves for compounds showing above 50% inhibition at 2 µM test concentration were determined.

### Chemistry

The target compounds (11–58) were synthesized as depicted in **Schemes 1** and **2**. The syntheses of compounds 11–22, 24–26, 31–33, 36, 38–40, 42–44, 46, 49–52, 54–56, and 58 had been previously described (Baqi and Müller, 2007; Weyler et al., 2008; Baqi et al., 2009b; Baqi et al., 2010; Baqi and Müller, 2010; Baqi et al., 2011; Baqi and Müller, 2012; Fiene et al., 2016; Malik et al., 2016). In addition to previously reported AQ derivatives, a series of 14 new compounds (23, 27–30, 34, 35, 37, 41, 45, 47, 48, 53, and 57) was prepared. Condensation of sodium 1-amino-4-bromo-9,10-dioxo-9,10-dihydroanthracene-2-sulfonate (R^1^ = SO_3_Na, 10a, **Scheme 1**), 1-amino-2,4-dibromo-9,10-dioxo-9,10-dihydroanthracene (R^1^ = Br, 10b, **Scheme 1**), or 1-amino-4-bromo-2-methyl-9,10-dioxo-9,10-dihydroanthracene (R^1^ = CH_3_, 10c, **Scheme 1**) with the appropriate (ar)alkylamine or aniline derivatives yielded the target compounds in satisfactory to excellent isolated yields. Anthraquinones 11–51 bearing a sulfonate substitution at the 2-position were synthesized starting from compound 10a in sodium phosphate buffer (pH 6−7) in the presence of a catalytic amount of elemental copper (Cu^0^) under microwave reaction conditions at 80−120°C for 5–24 min (Baqi and Müller, 2007; Baqi and Müller, 2010).

Compounds 52–56, bearing a bromo or methyl residue at the 2-position, were synthesized starting from 10b or 10c, respectively, with excess of the appropriate aniline derivatives (15 eq.) under argon in the presence of potassium acetate and copper(I) acetate (CuOAc) as a catalyst, upon heating at 110°C for 2–15 h (**Scheme 1**).

In order to investigate the role of the amino group at the 1-position of the anthraquinone moiety, two anilinoanthraquinone derivatives (21 and 33) were treated with sodium nitrite in hydrochloric acid solution (1 M) at 0–5°C for 5 min, then allowed to warm up to room temperature, followed by the addition of ethanol and an excess of zinc powder (10 equiv.) to achieve deamination within 30 seconds (Baqi and Müller, 2012), affording the desired products 57 and 58 in excellent yields (**Scheme 2**).

### Biological Studies

Inhibition of human NTPDases was performed using the malachite green assay, which was established on a robotic system (*Z*’ factors > 0.70) (Baykov et al., 1988; Fiene et al., 2015). The malachite green assay enables the detection of the phosphate produced by the enzymatic hydrolysis of nucleotides. A fixed substrate concentration of 50 µM ATP for NTPDase1 and 100 µM for NTPDase2, -3, and -8 was employed. Test compounds were initially screened at a concentration of 2 μM. For compounds that showed about 50% inhibition or more, concentration-dependent inhibition curves were determined, and IC_50_ values were calculated. A total of 48 synthesized anthraquinone derivatives including 14 new compounds not previously described in the literature were evaluated for their inhibitory activity at human NTPDase1, -2, -3, and -8 (for results see **Table 1**).

### Structure-Activity Relationships (SARs)

The anthraquinone derivative reactive blue-2 (RB-2 (**5**), **Figure 1** and **Table 1**) showed the highest potency at NTPDase3 (IC_50_ of 0.942 µM) followed by NTPDase2 and was inactive at NTPDase8 (**Table 1**). RB-2 is a relatively large molecule (molecular weight of >800 g/mol) with high polarity bearing three negatively charged sulfonate (SO_3_Na) groups. Therefore, smaller and less polar anthraquinone derivatives were designed, synthesized, and evaluated as NTPDases inhibitors (see **Table S1** in **Supplementary Materials** for clogD values of all anthraquinone derivatives discussed in the present study).

In our previous study, we had investigated a smaller series of anthraquinone derivatives at ecto-NTPDases of rat, which had led to the identification of PSB-071 (6) bearing a *m*-methyl substituent on the 4-anilino group. This inhibitor was slightly selective for rat NTPDase2 (12.8 μM) (Baqi et al., 2009b; Zebisch et al., 2014) vs. rat NTPDase1 and -3, while in the present study, it showed no significant inhibitory activity on all tested human NTPDases (compound 6, **Table 1**), except for NTPDase2, at which it displayed very moderate potency.

Introducing of an (ar)alkyl group, such as propyl (11), benzyl (12) and phenethyl (13) at the 4-amino group of 1-amino-2-sulfoanthraquinone abolished the inhibitory activity on all tested NTPDases (entry 3–5, **Table 1**). Moreover, unsubstituted aromatic rings, such as phenyl, 1-naphthyl and 2-naphthyl, 14, 15, and 18 (**Table 1**), all showed no inhibitory activity as well. The naphthylamino-substituted anthraquinone derivatives 15 and 18 had shown good potency in our previous study at rat NTPDase3 (both) and at rat NTPDase1 (compound 18) indicating considerable species differences between rat and human NTPDases.

Interestingly, a combination between structures of 1-naphthyl and 2-naphthyl resulting in phenanthryl derivative 20, yielded a potent inhibitor of NTPDase2 which displayed no activity vs. NTPDase1, -3, and -8 at the tested concentration. This is probably due to the presence of a large lipophilic pocket present in human NTPDase2. This presence of a lipophilic pocket in NTPDase2 was confirmed with compound 16 (IC_50_ of 5.62 µM, **Table 1**), which is bearing an extra lipophilic methyl group in the 2-position of the 1-naphthyl moiety; again, this compound was found to be selective vs. the other investigated human NTPDases (-1, -3, and -8). Introduction of polar and negatively charged groups, SO_3_H (17) or CO_2_H (19) on the naphthyl moiety shifted the inhibitory activity towards human NTPDase3.

In the next step, we introduced different substituents on phenyl ring D (compounds 21–38, **Table 1**). Mono-substitution of the aromatic ring D with Br (22), NO_2_ (24), CO_2_H (26), or CH_2_CO_2_H (30) in the *meta*-position led to selective inhibition of NTPDase3, while other mono-substitutions including *m*-F, *p*-NH_2_, *o*-CH_2_OH, *m*-CH_2_OH, and *p*-CH_2_OH resulted in no inhibition at all tested NTPDases. On the other hand, di-substitution with polar functions, e.g., NH_2_, SO_3_H, and OH, on the *meta*- and *para*-position of the phenyl ring restored the inhibitory potency towards NTPDase2, especially compound 33 showing inhibitory potency at submicromolar concentration. Any polar substituent in the *ortho*-position and in combination with a substituent in the *meta*- or *para*-position led to inactive derivatives. The introduction of lipophilic substituents in the *ortho*- and *para*-position shifted the inhibitory potency towards NTPDase2, see compound 38 (**Table 1**).

Next, we introduced an additional aromatic residue, ring E. Lipophilic substitution in the *meta*- and *para*-position resulted in moderate to good potency at NTPDase2 (39–41 and 43–51, **Table 1**), with potencies reaching the submicromolar range (IC_50_ of 0.551 μM, 48), while a *m*-dichlorotriazinyl moiety in combination with a *p*-SO_3_H group furnished the most potent compound of the present anthraquinone series at NTPDase3 (42, IC_50_ of 0.390 μM, **Table 1**).

Any modification on the anthraquinone moiety, such as removal of the amino group in position 1 or replacement of the sulfonate function in position 2 of the anthraquinone core by bromo or methyl abolished the inhibitory activity (see compounds 52–58, **Table 1**).

**Figure 2:**
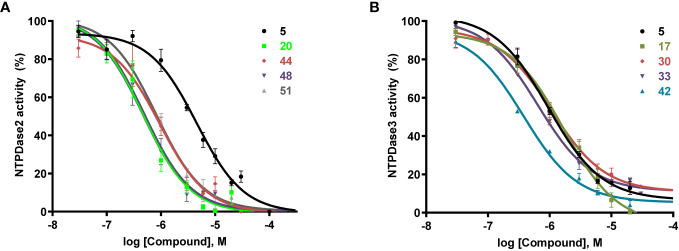
Concentration-inhibition curves of selected anthraquinone derivatives **(A)** determined using the malachite green assay on recombinant human NTPDase2 expressed in COS7 cell membrane preparations. ATP at a concentration of 100 µM (*K_m_* = 70 µM) was used as substrate and **(B)** determined using the malachite green assay on recombinant human NTPDase3 expressed in COS7 cell membrane preparations. ATP at a concentration of 100 µM (*K_m_* = 75 µM) was used as substrate. Data points shown are mean values of at least three independent experiments. IC_50_ values are collected in **Table 1**.

Concentration−response curves for selected potent anthraquinone derivatives 20, 44, 48, and 51 on NTPDase2 and for 17, 30, 33, and 42 on NTPDase3 are depicted in **Figure 2**.

**Figure 3:**
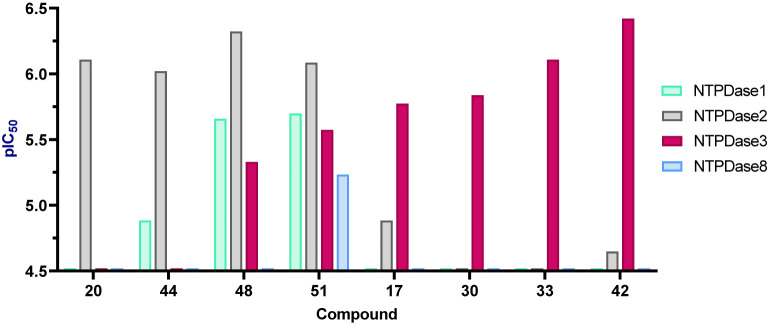
Selectivity of selected anthraquinone derivatives on different human NTPDase ecto-enzymes determined using the malachite green assay. Shown are the pIC_50_ values of compounds 20, 44, 48 and 51, active on NTPDase 2 and compounds 17, 30, 33 and 42 active on NTPDase3.

The most potent inhibitors were found to be selective vs. other tested human NTPDases. For examples, the NTPDase2 inhibitors 20 and 44 were found to be selective vs. NTPDase1, -3, and -8, while the other two most potent NTPDase2 inhibitors 48 and 51 showed lower selectivity (**Figure 3**). The identified NTPDase3 inhibitors 17, 30, 33 and 42 were selective vs. NTPDase1, -2, and -8 (**Figure 3**).

**Figure 4:**
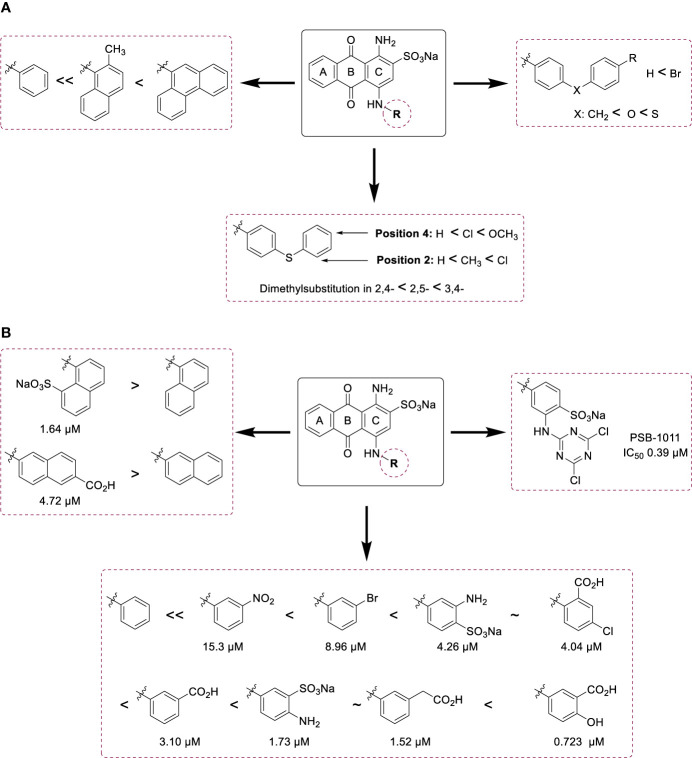
Summary of SARs of anthraquinone derivatives **(A)** at human NTPDase2 and **(B)** at human NTPDase3.

The SARs for human NTPDase2 and -3 are summarized in **Figure 4**. Large and lipophilic substituents have led to selectivity for NTPDase2 (**Figure 4A**), while smaller and polar substituent have provided selectivity for NTPDase3 (**Figure 4B**).

We previously published articles highlighting the fact that the anthraquinone scaffold represents a privileged scaffold in medicinal chemistry targeting different nucleotide-binding proteins including ecto-5’-nucleotidase, P2Y_12_ and P2X2 receptors (Baqi, 2016; Malik and Müller, 2016). However, this does not mean that potent compounds are non-selective. In fact, selectivity for specific targets has been achievable (Baqi et al., 2009a; Baqi, 2016; Malik and Müller, 2016; Rafehi et al., 2017a; Rafehi et al., 2017b). Compounds that are highly potent at a specific target typically also have shown selectivity. In a study published in 2010, we reported the first SARs of anthraquinone derivatives as inhibitors of rat ecto-5’-nucleotidase (CD73) (Baqi et al., 2010). The observed SARs were clearly different from the SARs of anthraquinone derivatives as NTPDase inhibitors. For example, compound 15 displayed an IC_50_ of 0.53 µM at rat CD73 but was virtually inactive at NTPDases, while compound 20, found to be a potent inhibitor of human NTPDase2 in the present study, was shown to be only weakly active against CD73 (58% inhibition at 1 mM concentration) (Baqi et al., 2010). The compounds have not yet been tested at alkaline phosphatase, but this enzyme has a very high *K_m_* value for adenine nucleotides, and its significance in the context of extracellular nucleotide metabolism and signaling in inflammation is therefore questionable. Nevertheless, ancillary activities of NTPDase inhibitors as blockers of CD73 or alkaline phosphatase would not be detrimental, but might even enhance their over-all effects leading to an accumulation of immunostimulatory, pro-inflammatory nucleotides while inhibiting the final production of immunosuppressive adenosine. Future studies might therefore be directed at multi-target drugs inhibiting more than one single ectonucleotidase.

### Mechanism of Enzyme Inhibition

**Figure 5:**
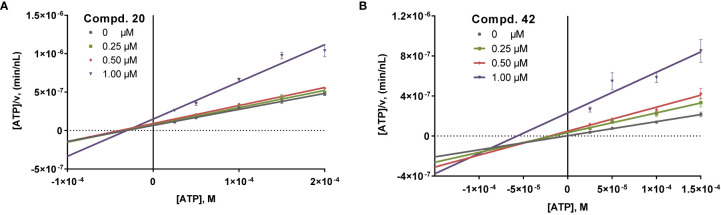
**(A)** Hanes-Woolf plot for NTPDase2 inhibition by 20, determined using the malachite green assay and recombinant human NTPDase2 expressed in COS7 cell membranes. ATP at concentrations of 25, 50, 100, 150 and 200 µM (*K_m_* = 70 µM) was used as a substrate. Data points shown are the mean values ± SEM of at least three independent experiments, each performed in triplicates (n = 3). **(B)** Hanes-Woolf plot for NTPDase3 inhibition by 42, determined using the malachite green assay and recombinant human NTPDase3 expressed in COS7 cell membranes. ATP at concentrations of 25, 50, 100, and 150 µM (*K_m_* = 75 µM) was used as a substrate. Data points shown are the mean values ± SEM of at least three independent experiments, each performed in triplicates (n = 3).

In previous studies at rat NTPDase2 and -3, selected small 1-amino-4-anilino-2-sulfoanthraquinone derivatives were found to display a competitive inhibition mechanism (Baqi et al., 2009b; Zebisch et al., 2014). In the present study at human NTPDases, the most potent inhibitors at NTPDase2, compound 20, and at NTPDase3, compound 42, were investigated with regard to their inhibition mechanism (see **Figure 5**).

NTPDase2 inhibitor 20 displayed non-competitive inhibition, while the larger NTPDase3 inhibitor 42 showed a mixed inhibition type. Together with previous results (Baqi et al., 2009b; Zebisch et al., 2014), these data show that anthraquinone derivatives may inhibit NTPDase isoenzymes with different inhibition mechanisms depending on the compound’s substitution pattern and perhaps also the NTPDase subtype and the species.

## Conclusions

Ectonucleoside triphosphate diphosphohydrolase (E-NTPDase) plays a major role in controlling extracellular nucleotide levels. NTPDase inhibitors have potential as novel drugs, for example, for the treatment of inflammation, neurodegenerative diseases and cancer. In the present study, we synthesized and investigated a series of 48 anthraquinone derivatives as potential inhibitors of NTPDases, 14 of which are novel compounds. The synthesized compounds showed no inhibitory activity on NTPDase1 (CD39) or NTPDase8, while potent inhibitors for NTPDase2 or -3 were identified. The most potent inhibitors exhibited selectivity for either NTPDase2 or -3. It was noticed that human NTPDase2 features a lipophilic pocket that accommodates polynuclear-aromatic rings such as phenanthryl or naphthyl bearing lipophilic substituents such as chloro or methyl. In contrast, NTPDase3 was found to accommodate smaller hydrophilic functions such as hydroxyl, carboxyl or sulfonate. These NTPDase3-inhibitors were selective (>10-fold) vs. other NTPDases. Although inhibitors bearing polar sulfonate functions cannot be expected to be brain-penetrant, they will be useful tools for studying peripheral effects, or maybe even used to study central effects after direct application to the brain.

## Data Availability Statement

The original contributions presented in the study are included in the article/**Supplementary Material**. Further inquiries can be directed to the corresponding authors.

## Author Contributions

CM and YB designed the study. YB, MR, and EM synthesized the compounds. LS and AF performed the biological assays. JS and JP expressed the NTPDases and produced the enzyme-containing membrane preparations. YB, CM, and MR wrote the manuscript with contributions from all coauthors. All authors contributed to the article and approved the submitted version.

## Funding

This study was supported by the DFG (SFB 1328, ID 335447717), the BMBF (BIGS DrugS), and the Arab-German Young Academy of Sciences and Humanities (AGYA) (01DL16002 & 01DL20003). EM is grateful to the Deutscher Akademischer Austauchdienst (DAAD) for a PhD scholarship. JS received support from the Natural Sciences and Engineering Research Council of Canada (NSERC; RGPIN-2016-05867) and was the recipient of a “Chercheur National” Scholarship from the Fonds de Recherche du Québec–Santé (FRQS).

## Conflict of Interest

The authors declare that the research was conducted in the absence of any commercial or financial relationships that could be construed as a potential conflict of interest.
