# Supplementary material for: Development of Anthraquinone Derivatives as Ectonucleoside Triphosphate Diphosphohydrolase (NTPDase) Inhibitors With Selectivity for NTPDase2 and NTPDase3
Source: Front Pharmacol. 2020 Aug 27;11:1282. doi: 10.3389/fphar.2020.01282 (PMC7481482; doi:10.3389/fphar.2020.01282)
Supplement: Supplementary file 1 [file DataSheet_1.pdf]

Supporting Information

Development of anthraquinone derivatives with selectivity for ectonucleoside triphosphate diphosphohydrolases (NTPDases) 2 and 3

Younis Baqi[1], Mahmoud Rashed[2,#], Laura Schäkel[2], Enas M. Malik[2], Julie Pelletier[3], Jean Sévigny[3,4] Amelie Fiene[2], and Christa E. Müller[2]**

[1]Department of Chemistry, Faculty of Science, Sultan Qaboos University, PO Box 36, Postal Code 123, Muscat, Oman, [2]PharmaCenter Bonn, Pharmaceutical Institute, Pharmaceutical & Medicinal Chemistry, University of Bonn, An der Immenburg 4, D-53121 Bonn, Germany, [3]Département de Microbiologie-Infectiologie et d'Immunologie, Faculté de Médecine, Université Laval, Québec, QC, Canada, [4]Centre de Recherche du CHU de Québec, Québec, QC, Canada

Keywords: Anthraquinone; CD39; inhibitor; metalloenzymes; neuroinflammation; NTPDase2; NTPDase3; synthesis

* Corresponding Authors: baqi@squ.edu.om; christa.mueller@uni-bonn.de

# On leave from the Department of Pharmaceutical and Medicinal Chemistry, Faculty of Pharmacy, Al-Azhar University, Cairo 11884, Egypt.

Table of Contents

Contents	Pages
**Table S1.** Calculated LogD of all anthraquinone derivatives (**5**, **6**& **11**–**58**) using Instant JChem version 5.3.4	**S2–S9**
**Figure S1–S14.** ^{1}H and ^{13}C-NMR spectra of compounds **23**, **27**–**30**, **34**, **35**, **37**, **41**, **45**, **47**, **48**, **53**, and **57**	**S10–S23**
**Figure S15–S28.** LC-MS spectrum of compounds **23**, **27**–**30**, **34**, **35**, **37**, **41**, **45**, **47**, **48**, **53**, and **57**	**S24–S37**

Table S1. Calculated LogD of all anthraquinone derivatives (**5, 6**& **11**–**58**) using Instant JChem version 5.3.4

Compound	Structure	ClogD at pH 7.4
**5**	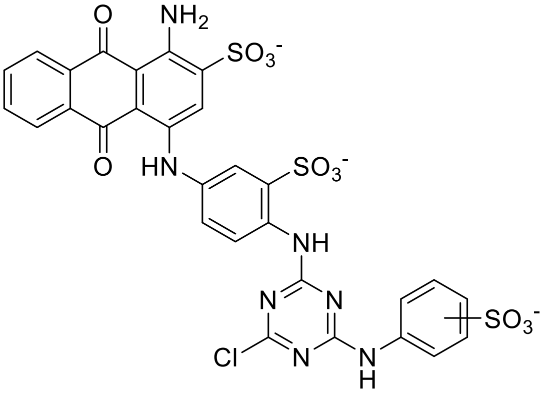	**-1.12**
**6**	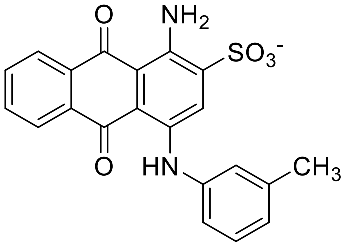	**2,80**
**11**	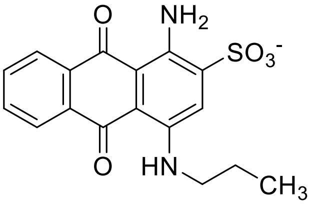	**0.55**
**12**	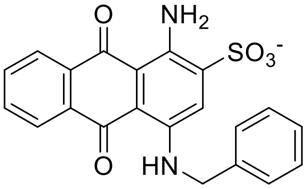	**1.39**
**13**	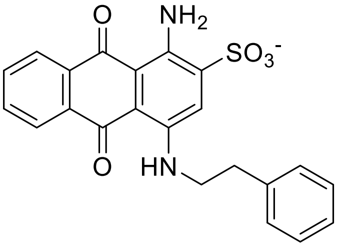	**1.68**
**14**	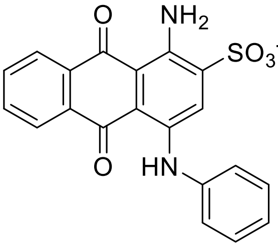	**2.28**

**15**	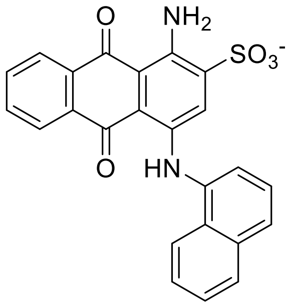	**3.27**
**16**	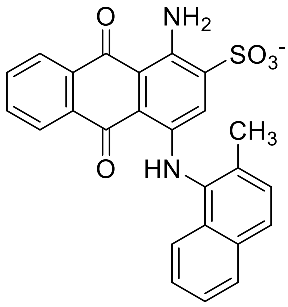	**3.79**
**17**	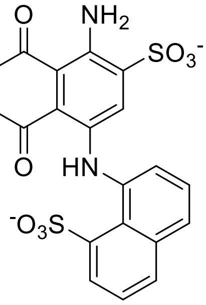	**0.08**
**18**	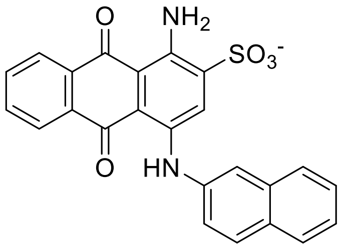	**3.27**
**19**	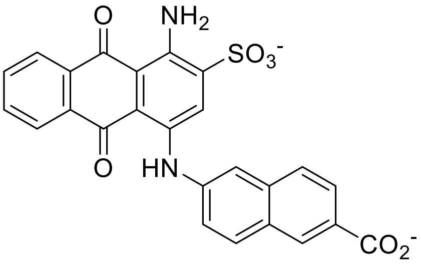	**-0,23**
**20**	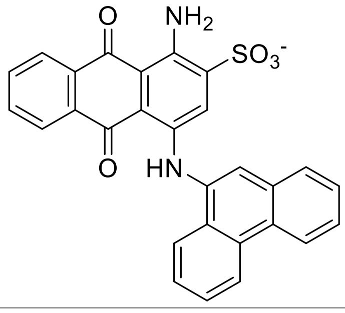	**4.26**

**21**	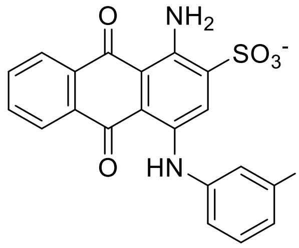	**2.43**
**22**	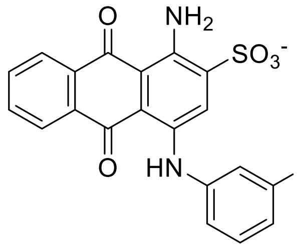	**3.05**
**23**	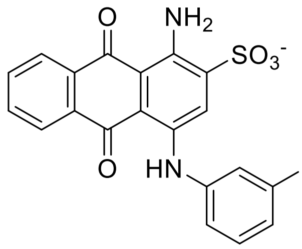	**3.21**
**24**	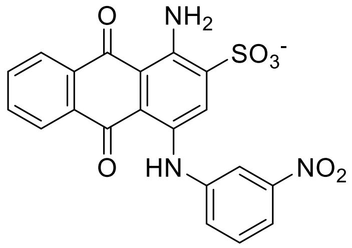	**2.22**
**25**	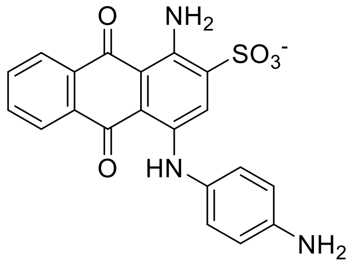	**1.45**
**26**	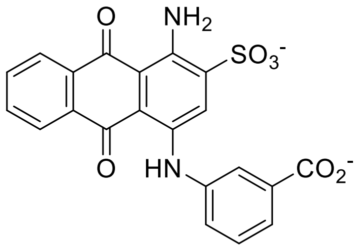	**-0.66**
**27**	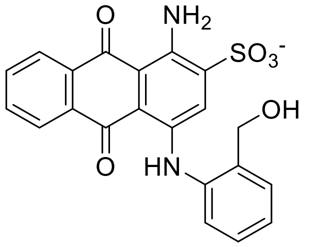	**1.52**

**28**	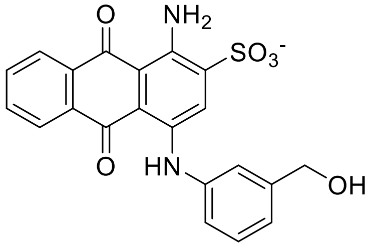	**1.52**
**29**	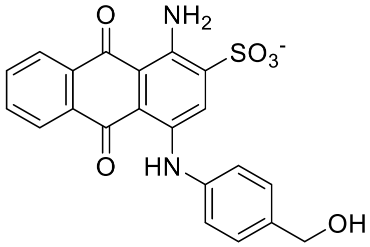	**1.52**
**30**	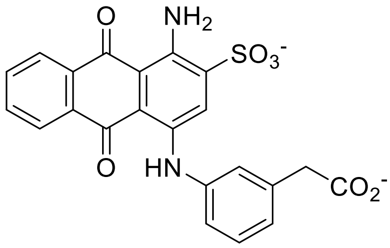	**-1.61**
**31**	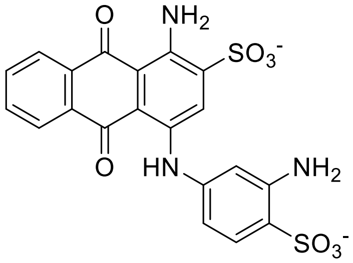	**-1.74**
**32**	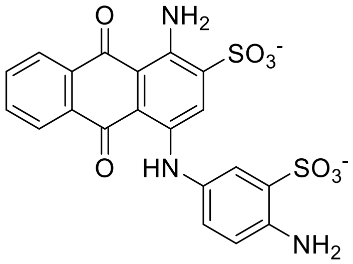	**-1.74**
**33**	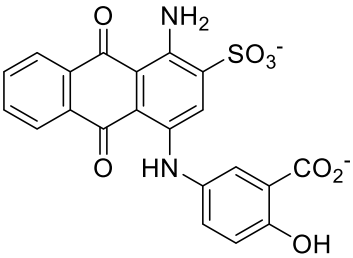	**-1.24**
**34**	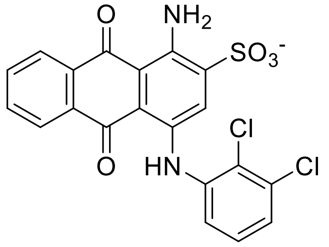	**3.49**

**35**	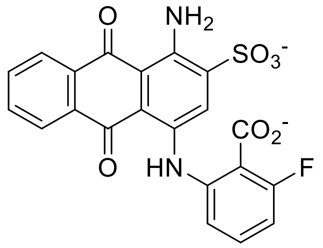	**-1.44**
**36**	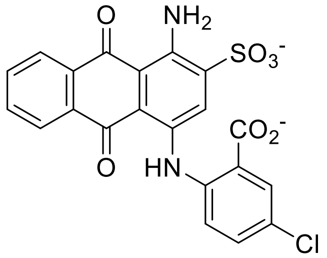	**-0.98**
**37**	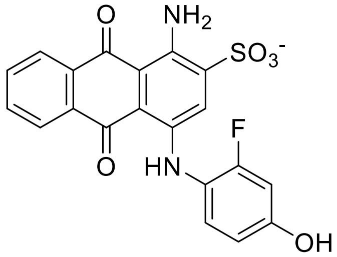	**2.10**
**38**	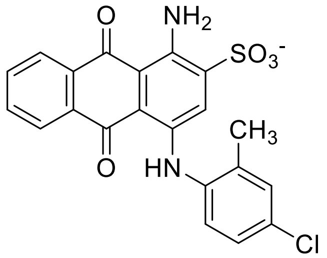	**3.40**
**39**	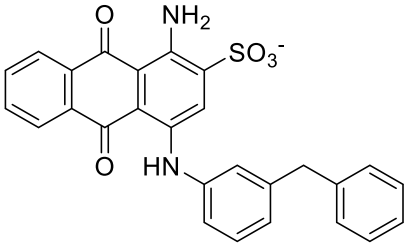	**4.38**
**40**	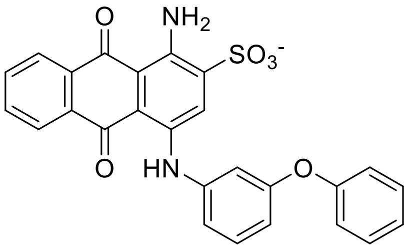	**3.78**
**41**	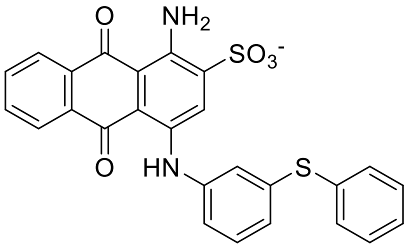	**4.47**

**42**	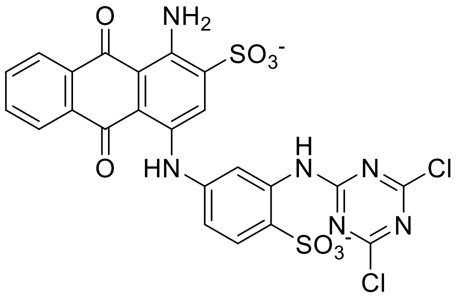	**0.77**
**43**	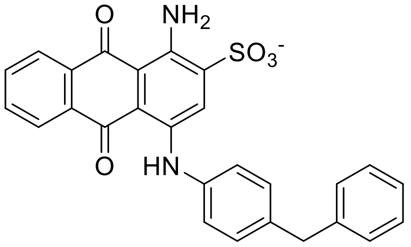	**4.38**
**44**	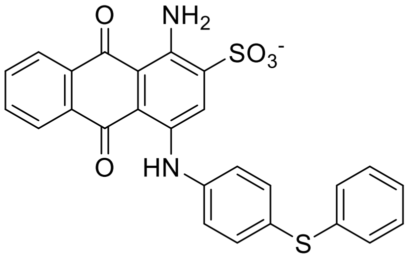	**4.47**
**45**	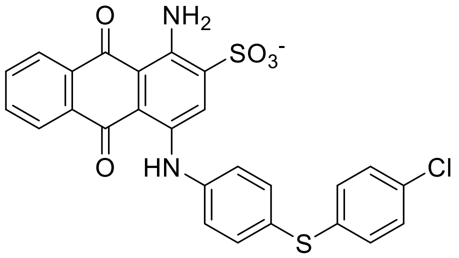	**5.08**
**46**	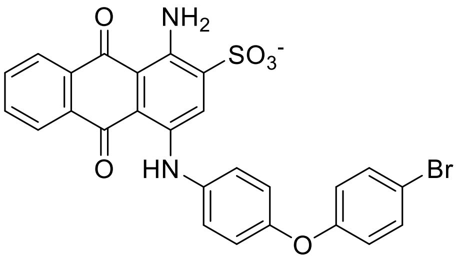	**4.55**
**47**	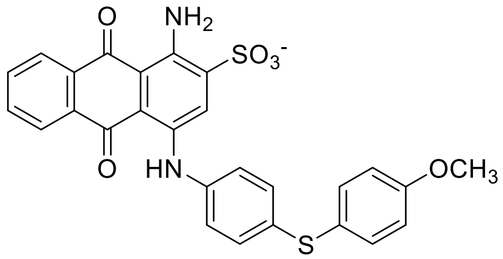	**4.32**

**48**	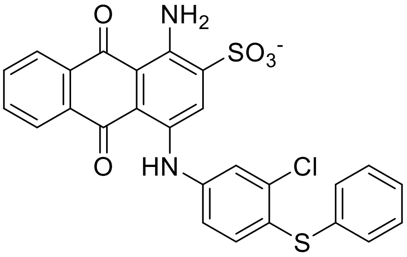	**5.08**
**49**	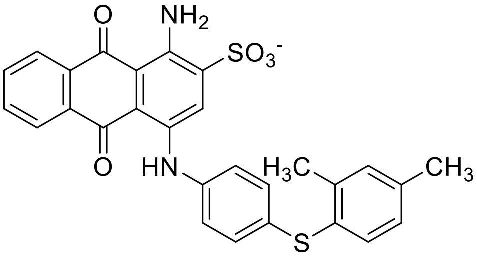	**5.50**
**50**	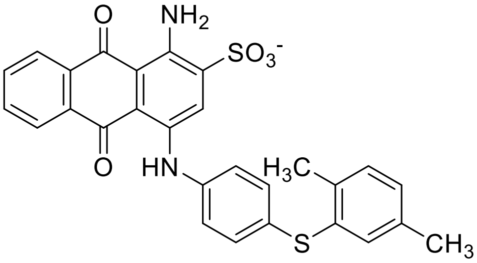	**5.50**
**51**	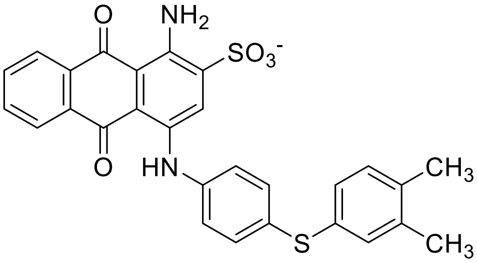	**5.50**
**52**	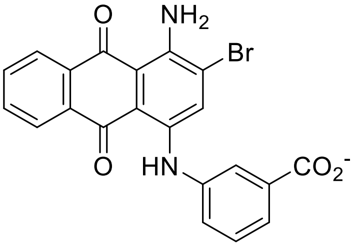	**3.31**
**53**	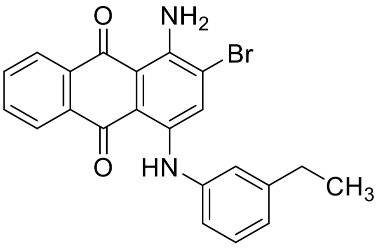	**7.21**
**54**	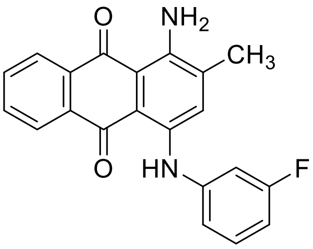	**6.14**

**55**	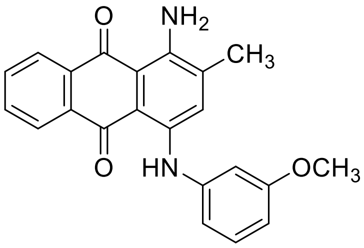	**5.84**
**56**	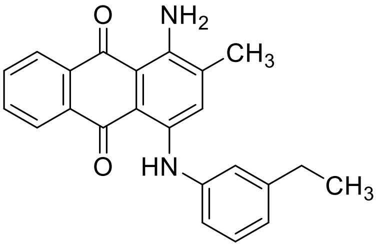	**6.95**
**57**	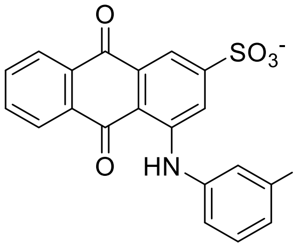	**2.61**
**58**	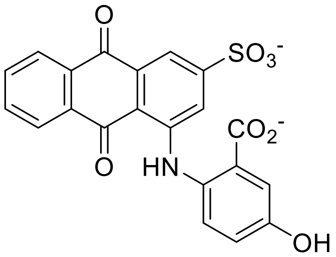	**-1.72**

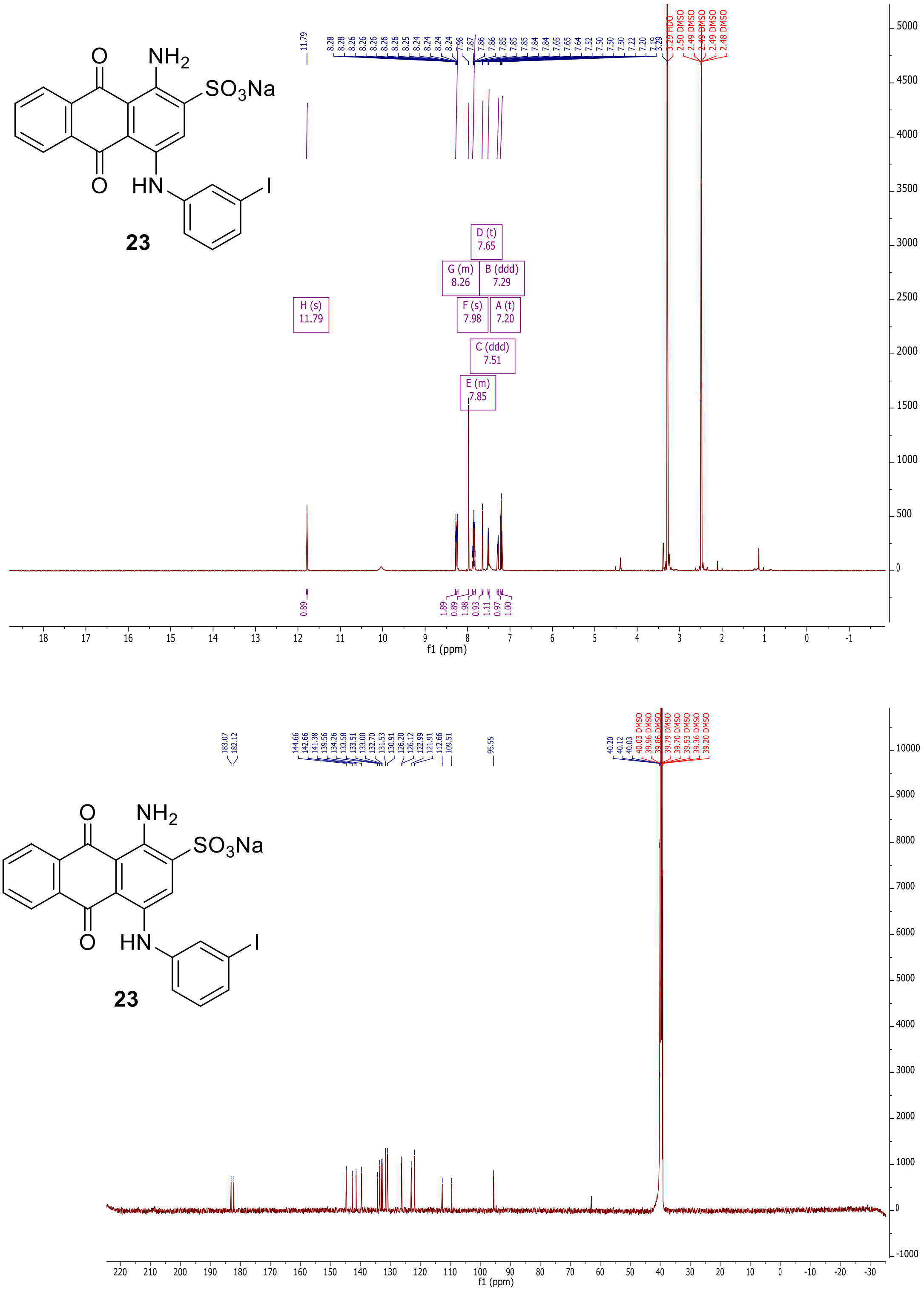

Figure S1. ^{1}H (500 MHz) and ^{13}C (126 MHz) spectra **sodium 1-amino-4-(3-iodophenylamino)-9,10-dioxo-9,10-dihydroanthracene-2-sulfonate (23)** in DMSO-d_6

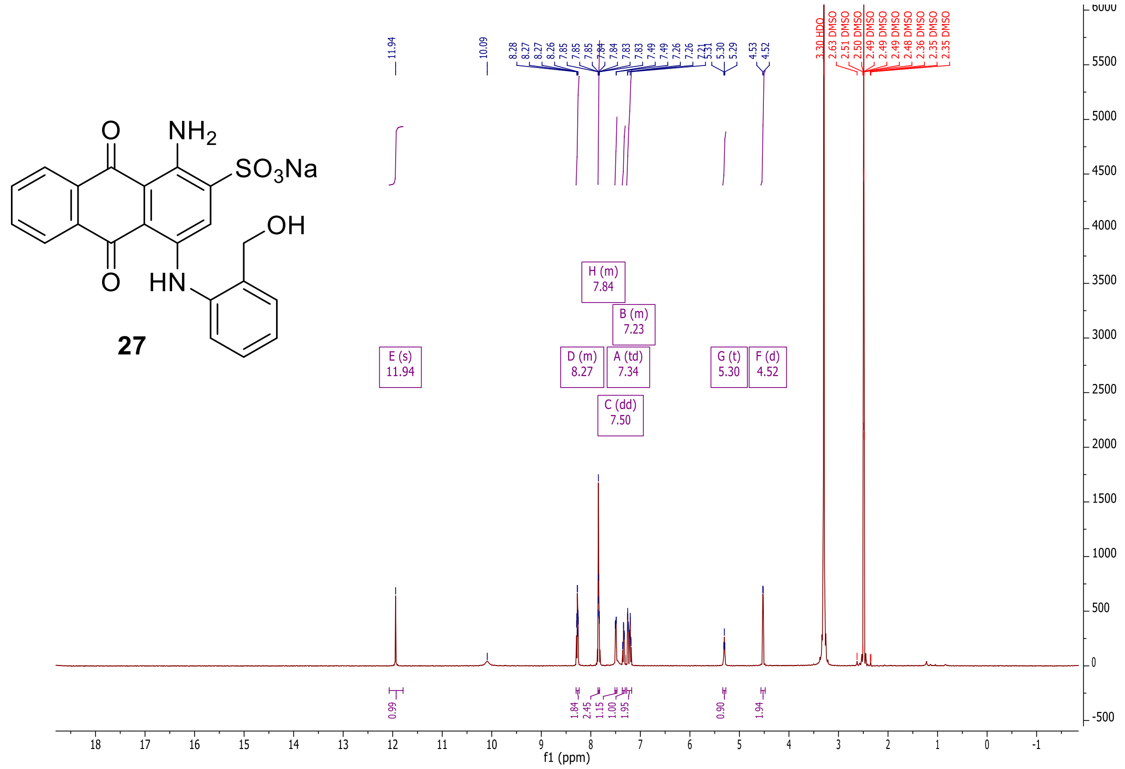

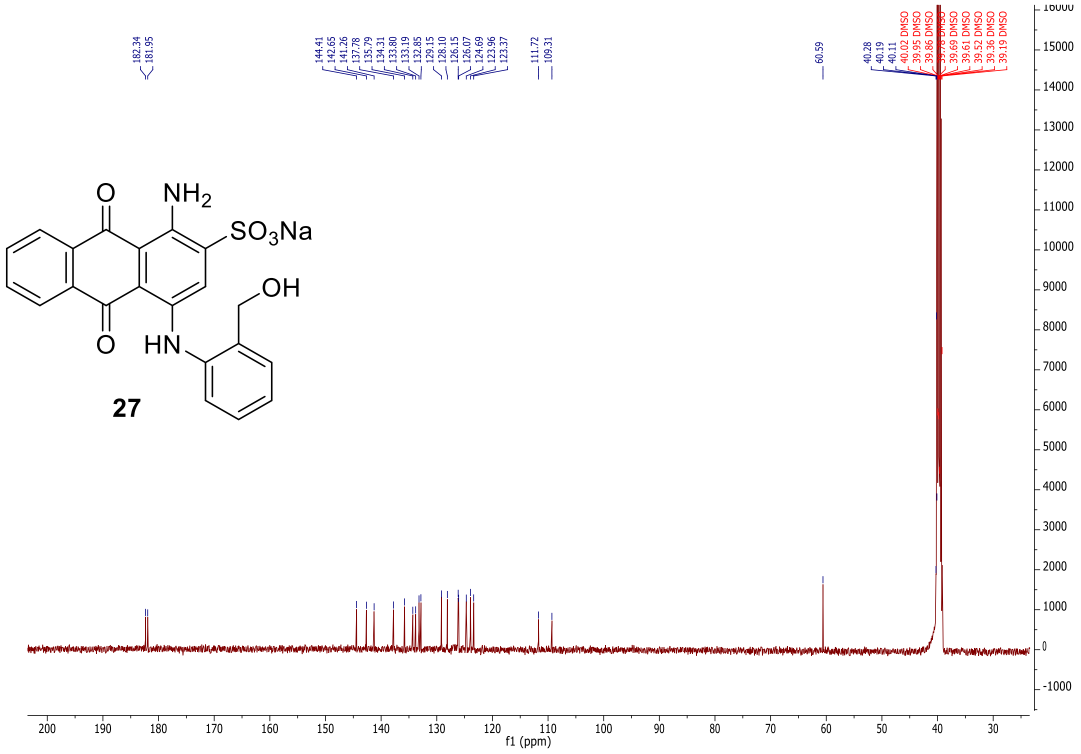

Figure S2. ^{1}H (500 MHz) and ^{13}C (126 MHz) spectra **sodium 1-amino-4-(2-(hydroxymethyl)phenylamino)-9,10-dioxo-9,10-dihydroanthracene-2-sulfonate (27)** in DMSO-d_6

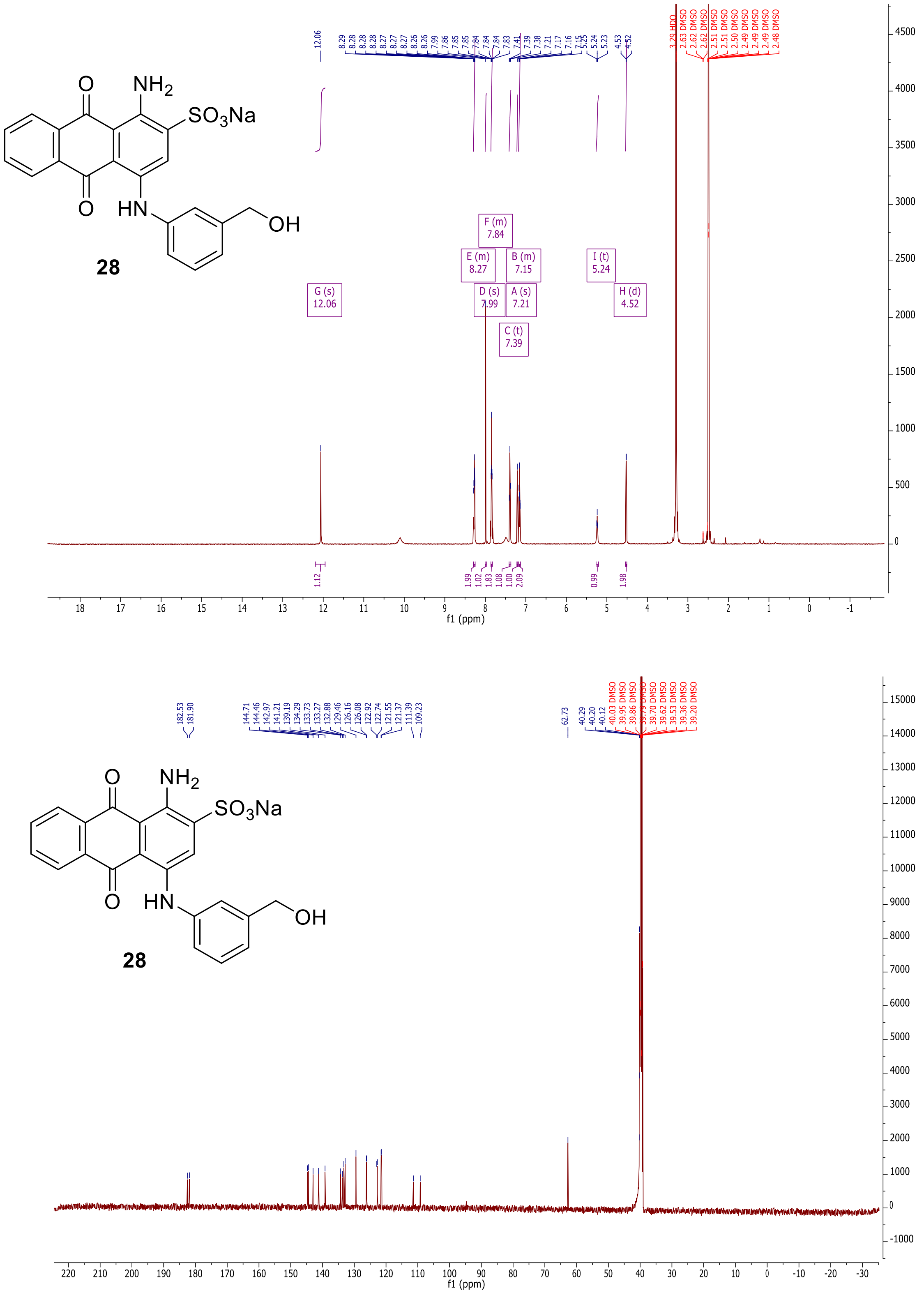

Figure S3. ^{1}H (500 MHz) and ^{13}C (126 MHz) spectra **sodium 1-amino-4-(3-(hydroxymethyl)phenylamino)-9,10-dioxo-9,10-dihydroanthracene-2-sulfonate (28)** in DMSO-d_6

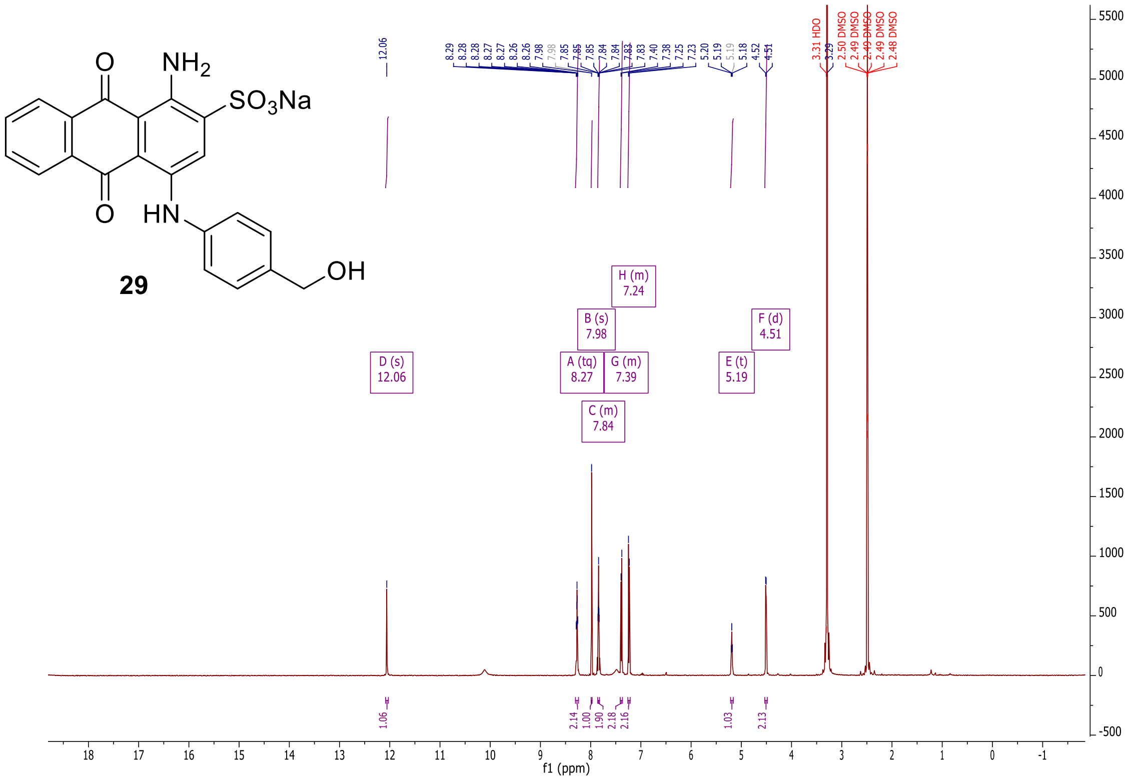

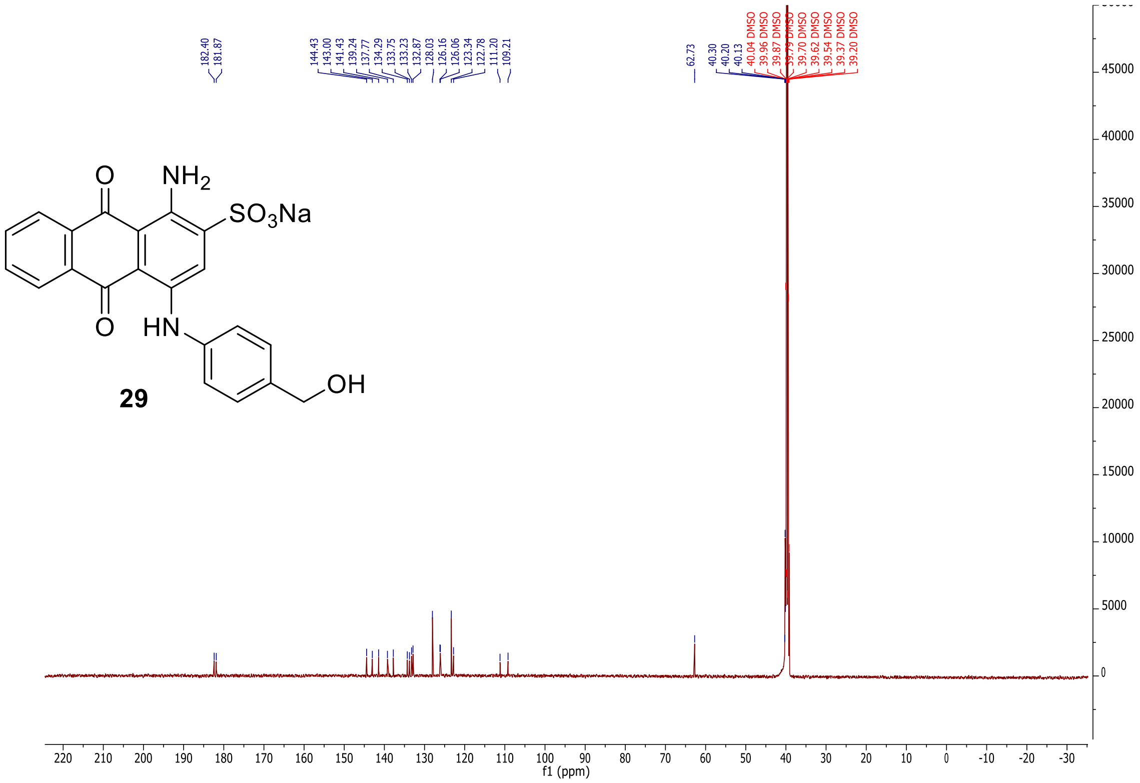

Figure S4. ^{1}H (500 MHz) and ^{13}C (126 MHz) spectra **sodium 1-amino-4-(4-(hydroxymethylphenylamino)-9,10-dioxo-9,10-dihydroanthracene-2-sulfonate** (**29**) in DMSO-d_6

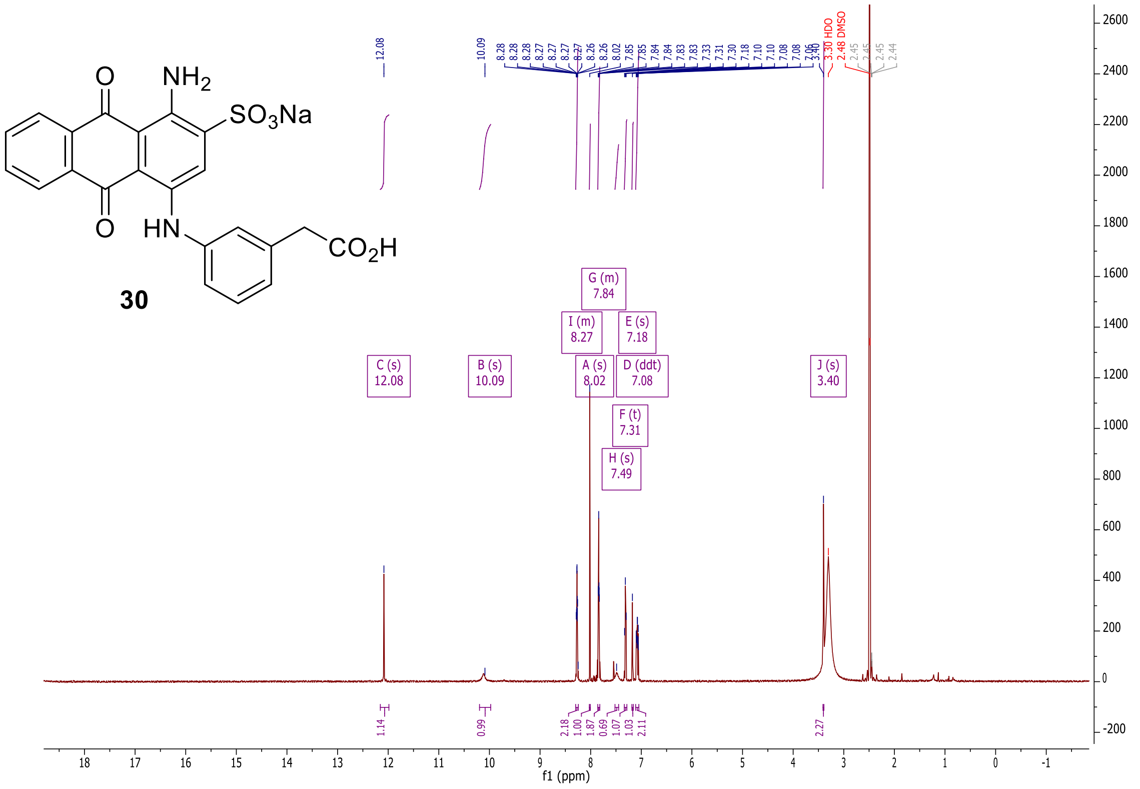

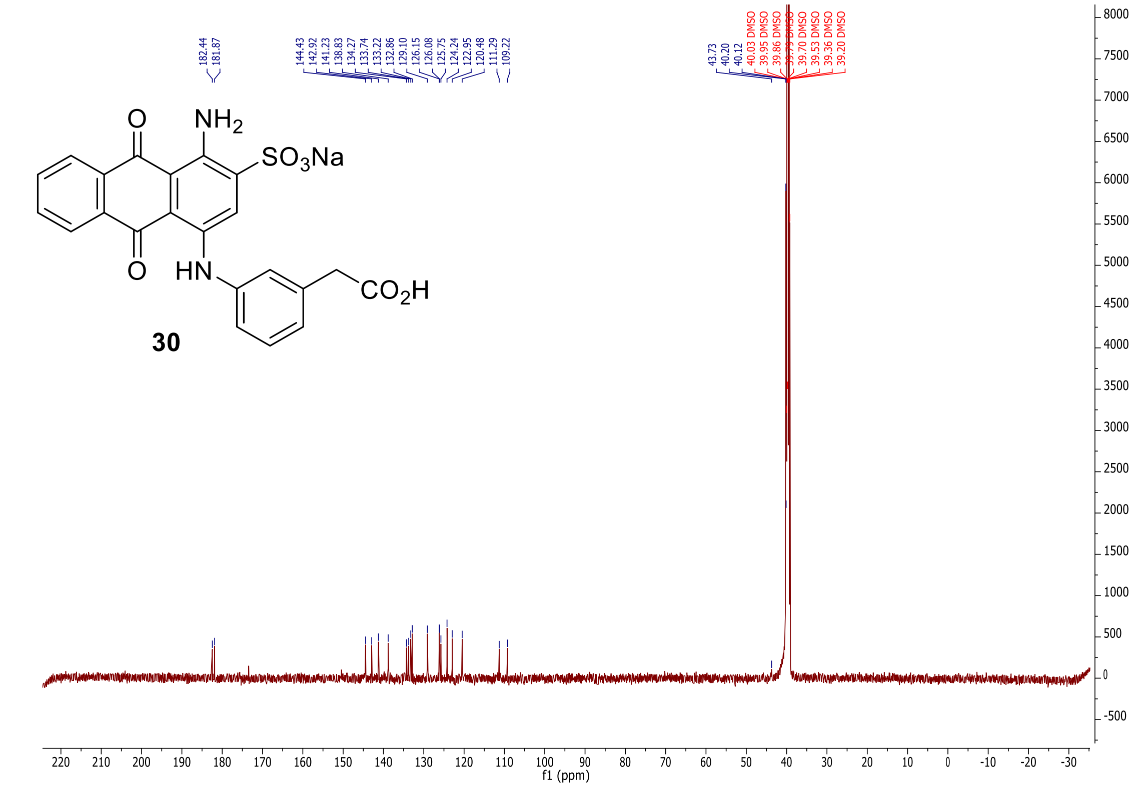

Figure S5. ^{1}H (500 MHz) and ^{13}C (126 MHz) spectra **sodium 1-amino-4-(3-(carboxymethyl)phenylamino)-9,10-dioxo-9,10-dihydroanthracene-2-sulfonate** (**30**) in DMSO-d_6

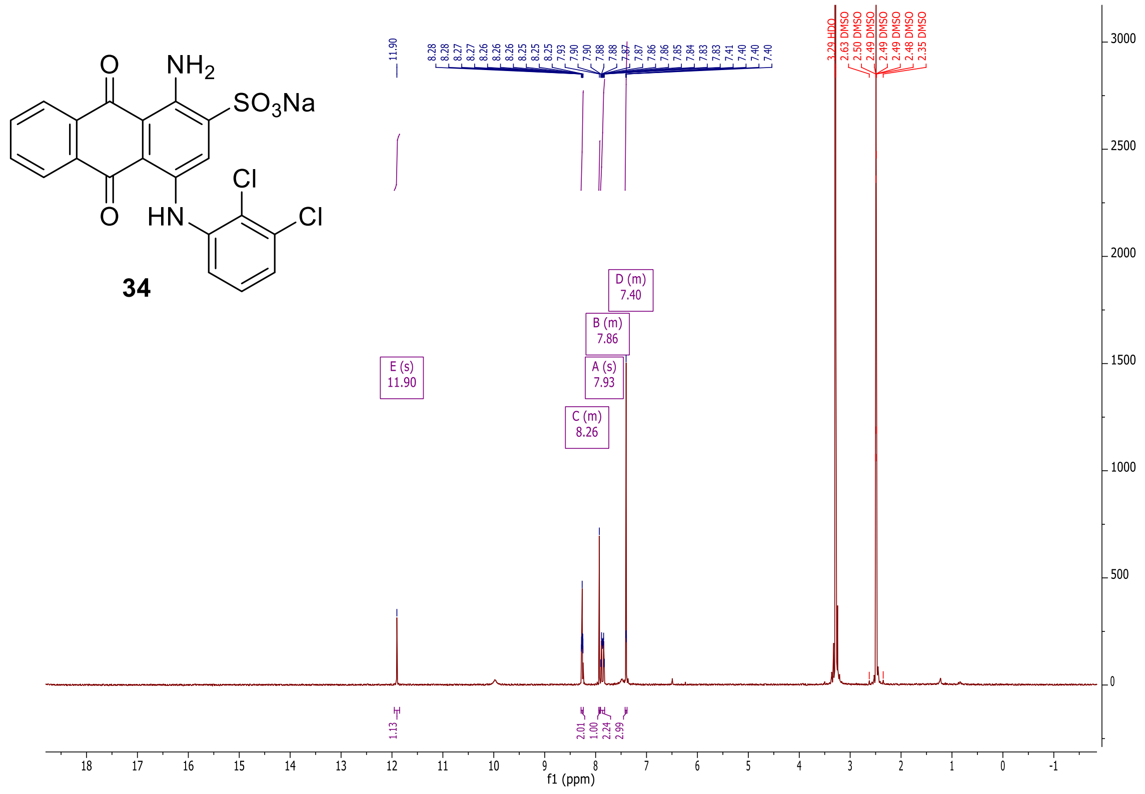

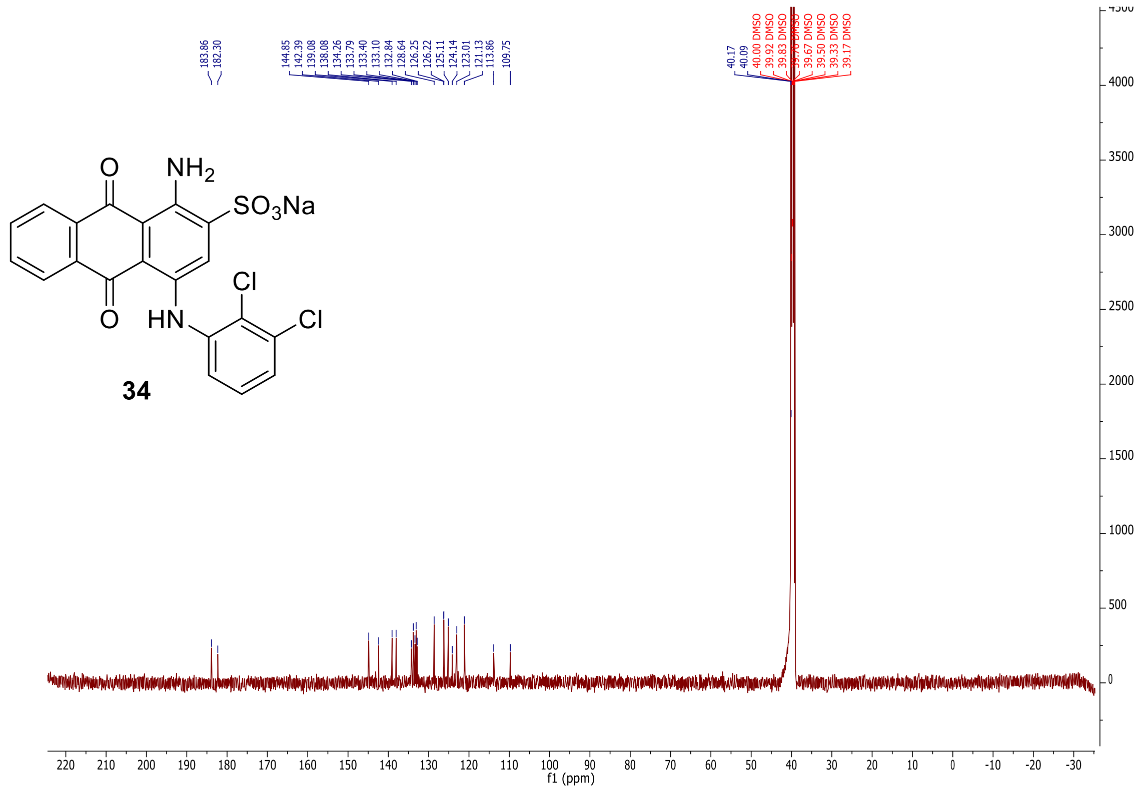

Figure S6. ^{1}H (500 MHz) and ^{13}C (126 MHz) spectra **sodium 1-amino-4-(2,3-dichlorophenylamino)-9,10-dioxo-9,10-dihydroanthracene-2-sulfonate (34)** in DMSO-d_6

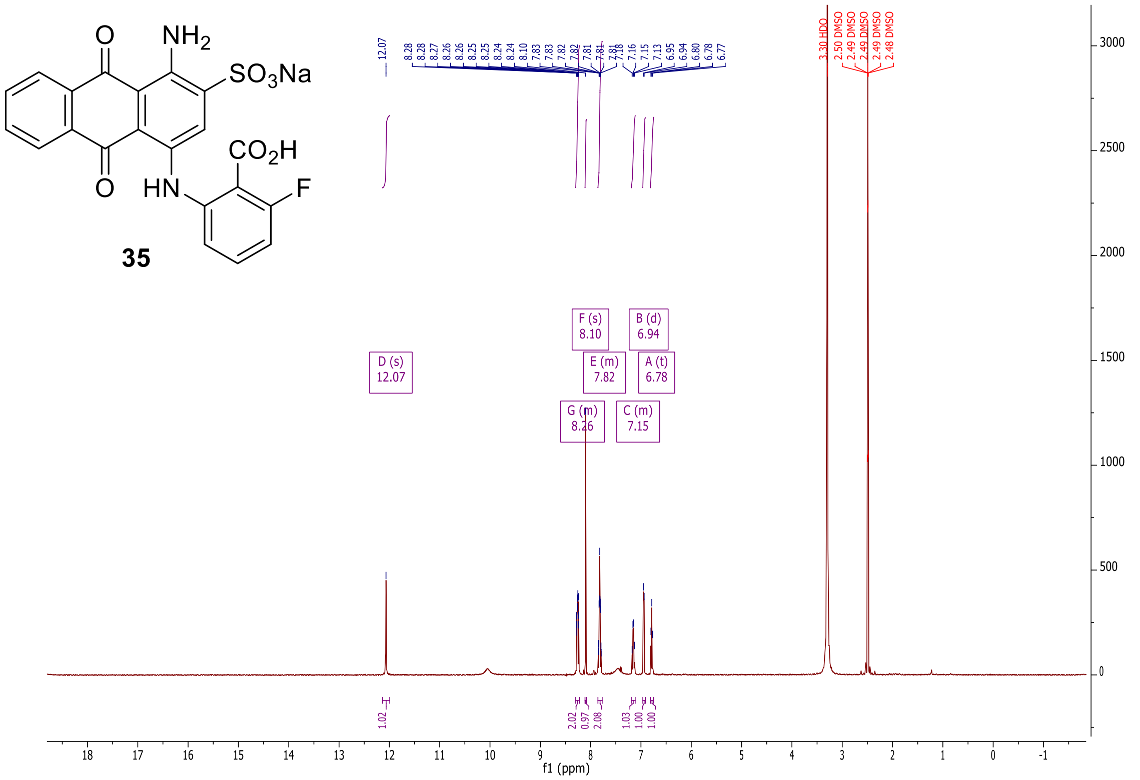

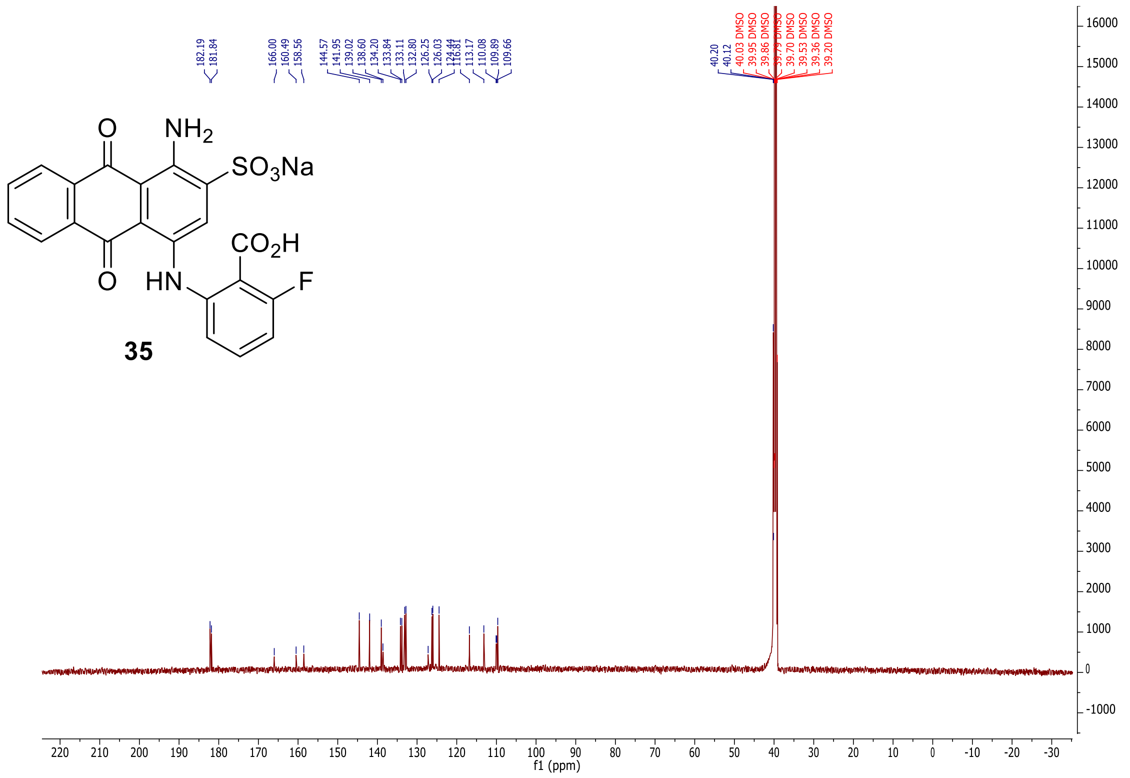

Figure S7. ^{1}H (500 MHz) and ^{13}C (126 MHz) spectra **sodium 1-amino-4-(2-carboxy-3-fluorophenylamino)-9,10-dioxo-9,10-dihydroanthracene-2-sulfonate (35)** in DMSO-d_6

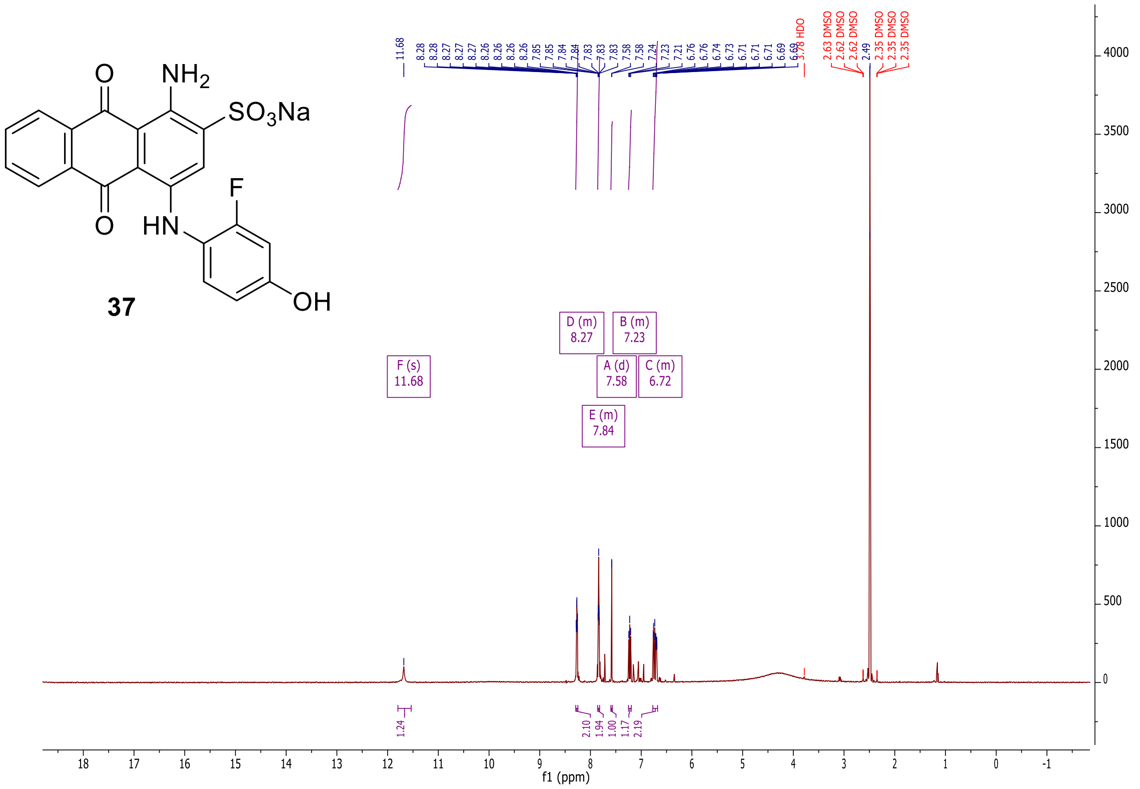

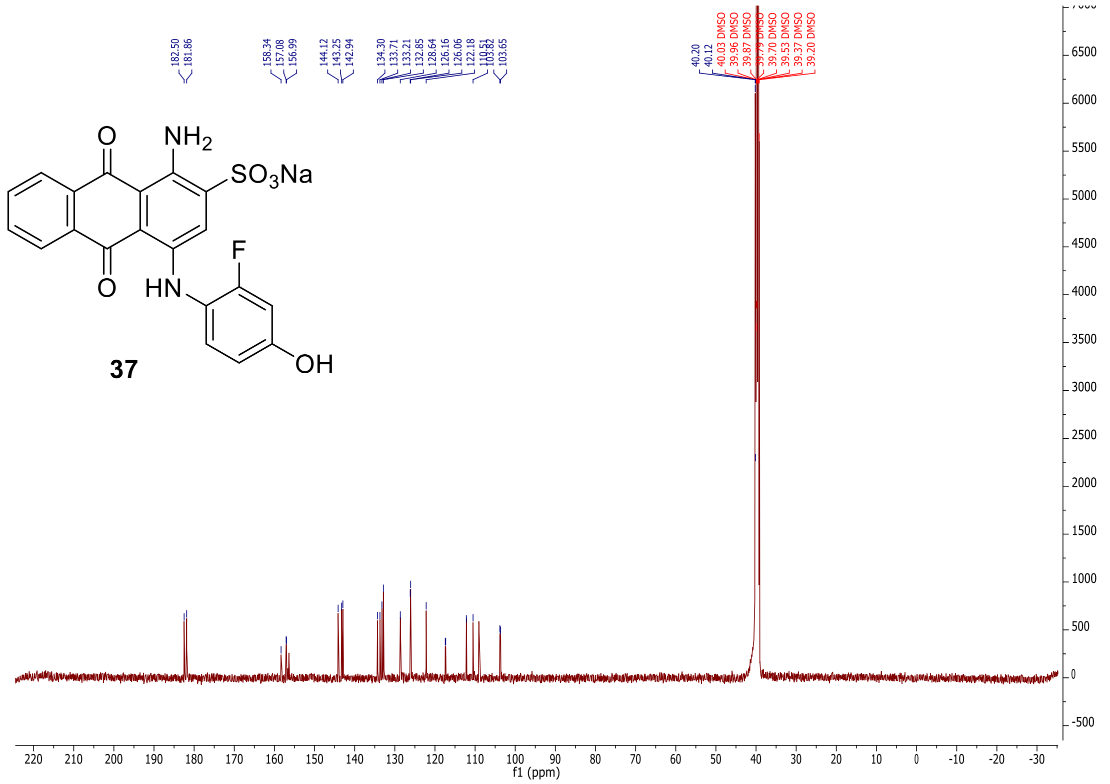

Figure S8. ^{1}H (500 MHz) and ^{13}C (126 MHz) spectra **sodium 1-amino-4-(2-fluoro-4-hydroxyphenylamino)-9,10-dioxo-9,10-dihydroanthracene-2-sulfonate (37)** in DMSO-d_6

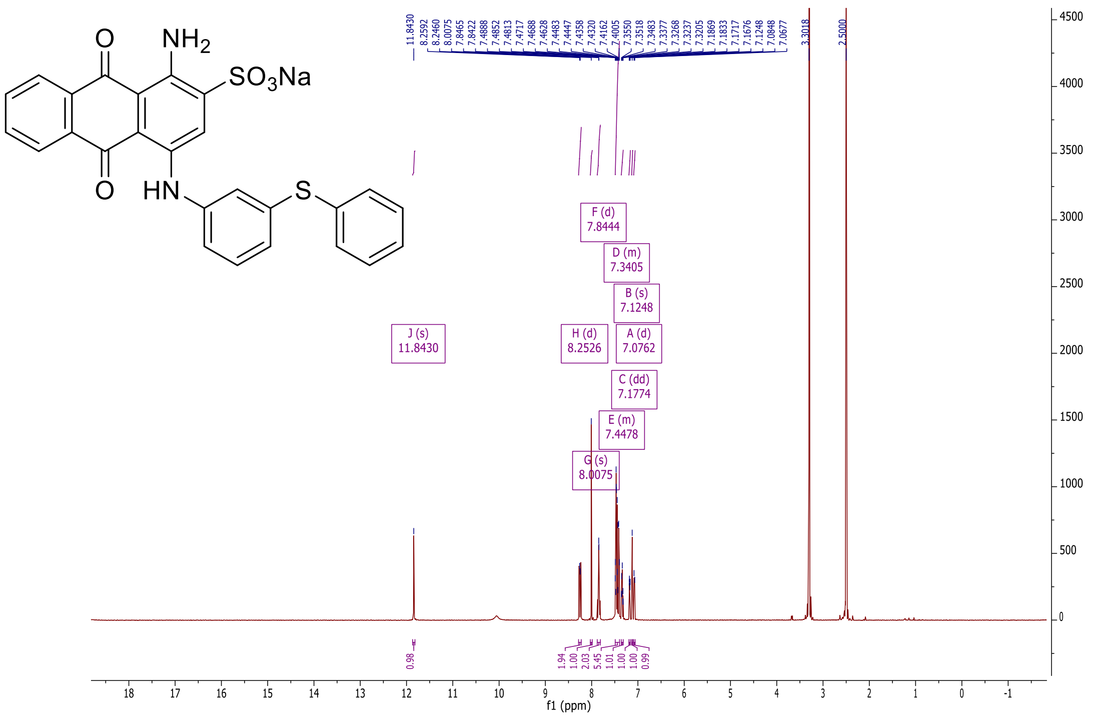

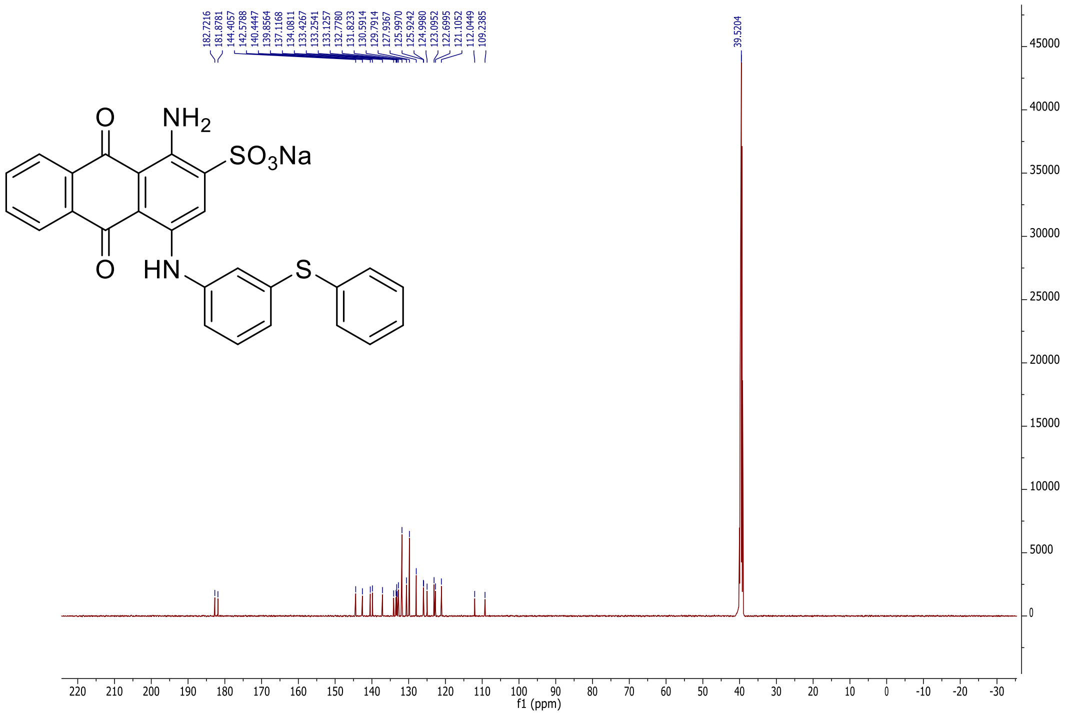

Figure S9. ^{1}H (500 MHz) and ^{13}C (126 MHz) spectra **sodium 1-amino-4-(3-phenylsulfanylphenylamino)-9,10-dioxo-9,10-dihydro-anthracene-2-sulfonate (41)** in DMSO-d_6

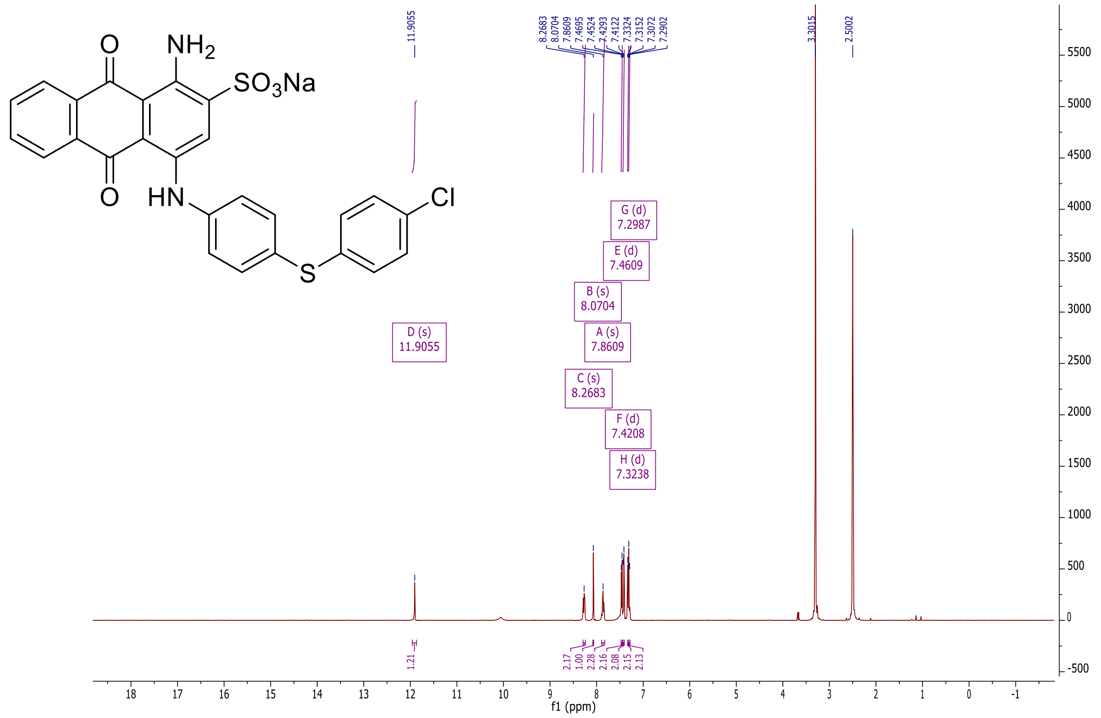

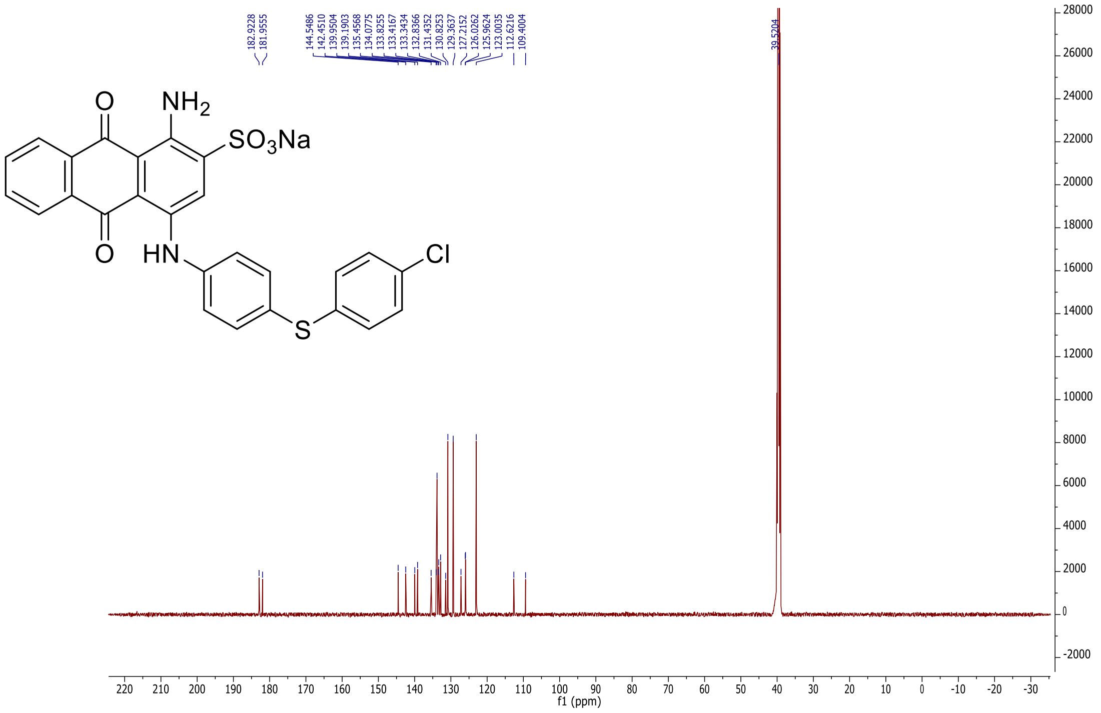

Figure S10. ^{1}H (500 MHz) and ^{13}C (126 MHz) spectra **sodium 1-Amino-4-[4-(4-chlorophenylthio)phenylamino]-9,10-dioxo-9,10-dihydroanthracene-2- sulfonate (45)** in DMSO-d_6

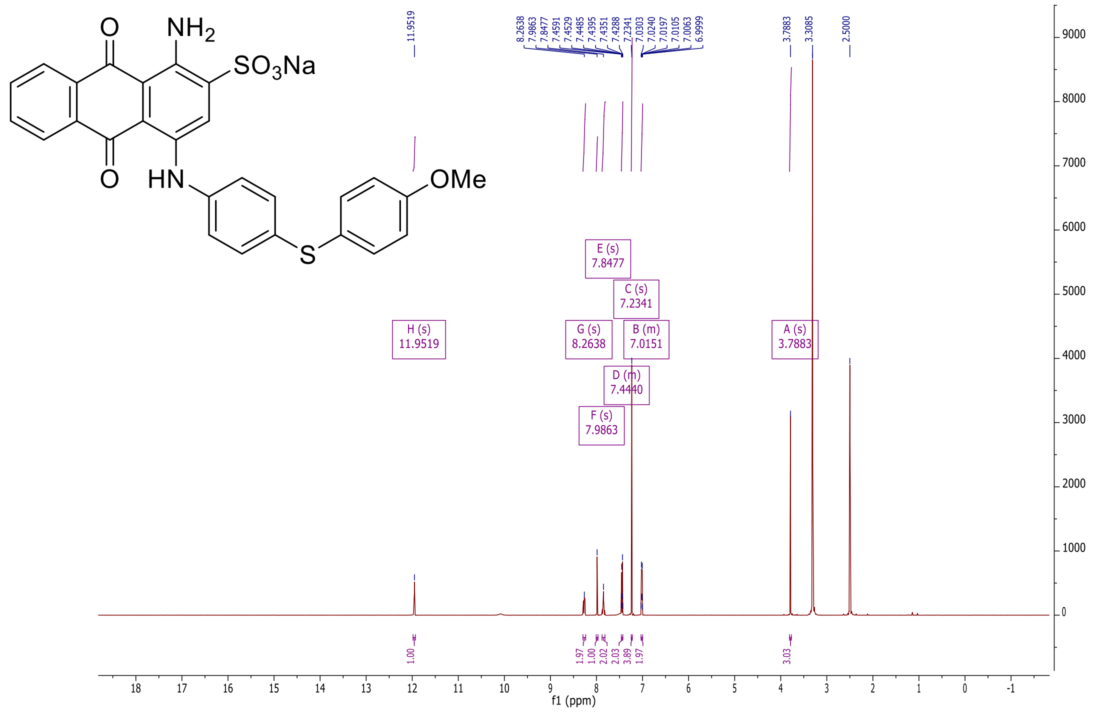

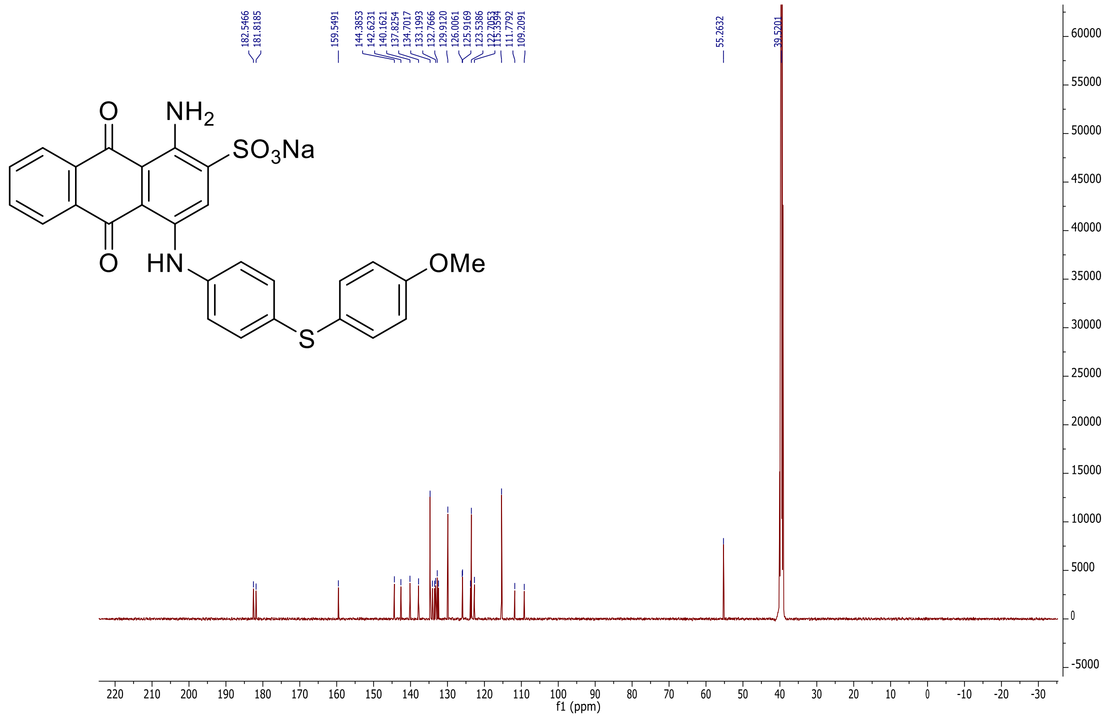

Figure S11. ^{1}H (500 MHz) and ^{13}C (126 MHz) spectra **sodium 1-amino-4-[4-(4-methoxyphenylthio)phenylamino]-9,10-dioxo-9,10-dihydroanthracene-2-sulfonate (47)** in DMSO-d_6

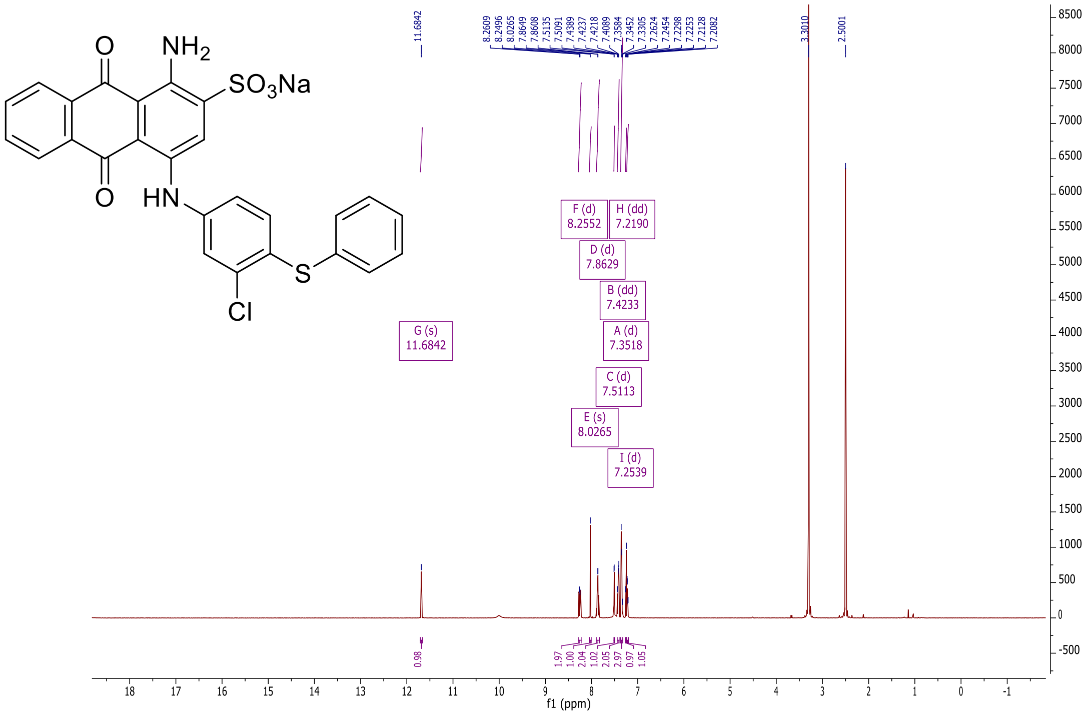

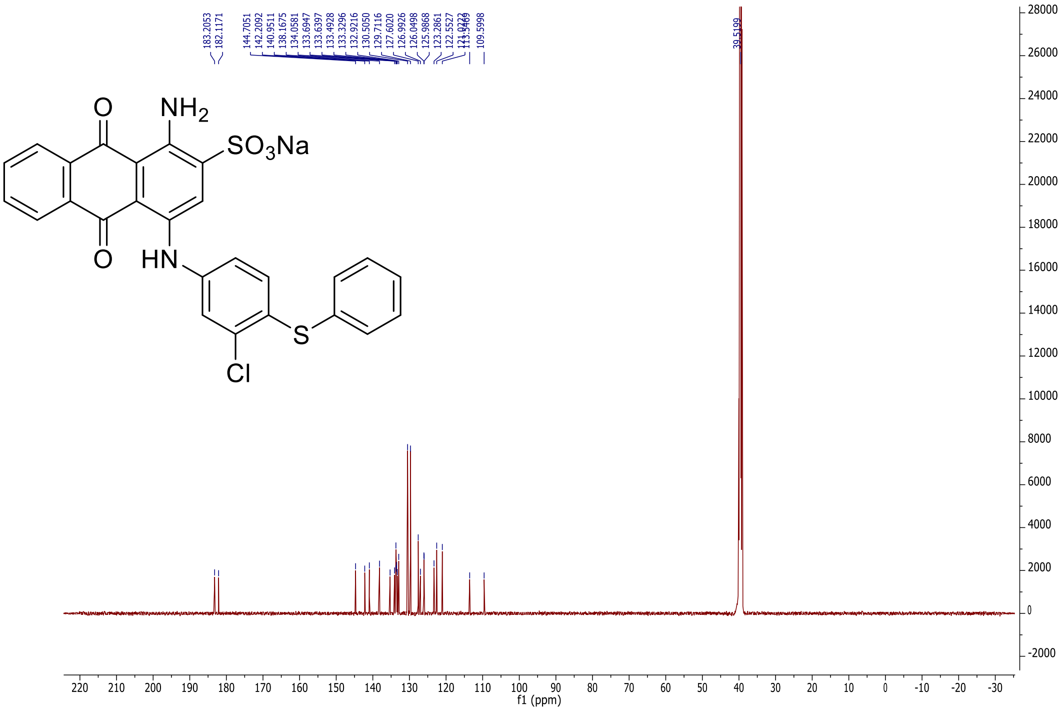

Figure S12. ^{1}H (500 MHz) and ^{13}C (126 MHz) spectra **sodium 1-amino-4-(3-chloro-4-phenylsulfanyl)phenylamino-9,10-dioxo-9,10-dihydro-anthracene-2-sulfonate** (**48**) in DMSO-d_6

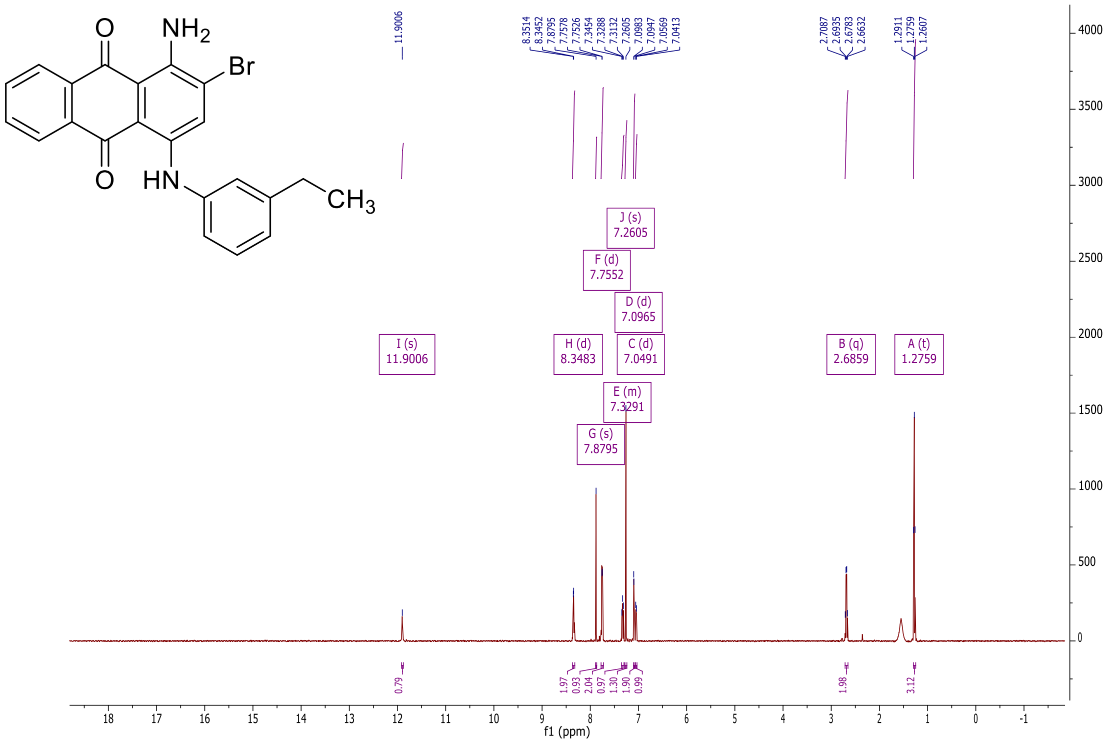

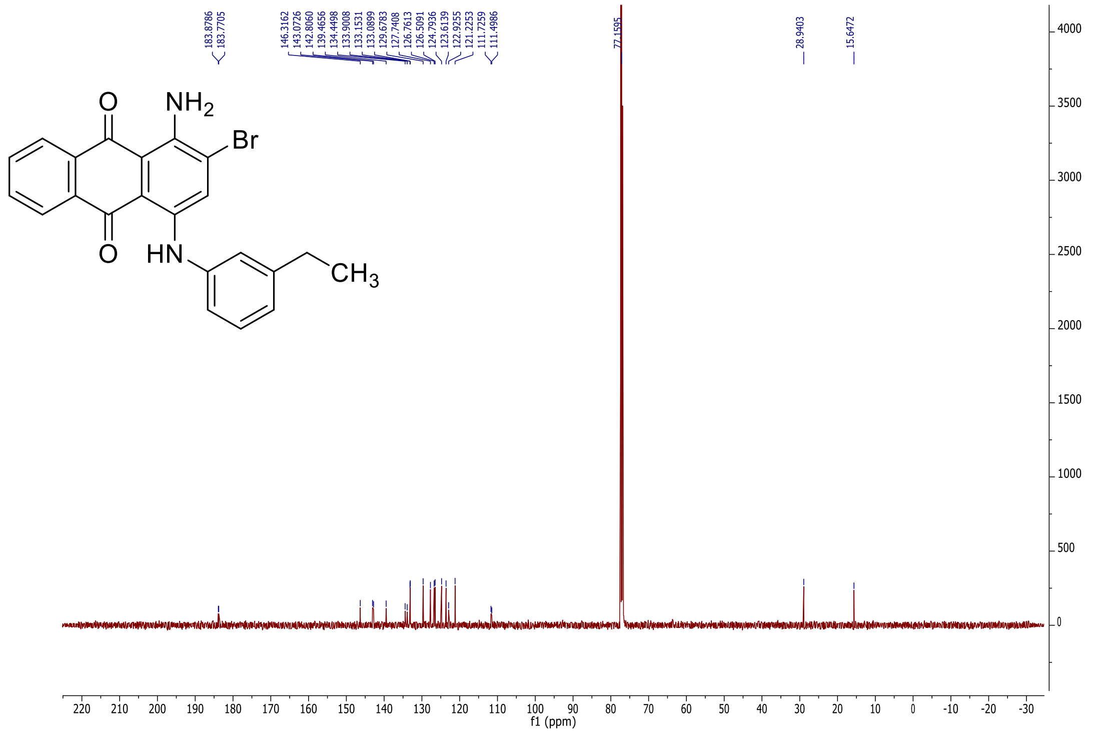

Figure S13. ^{1}H (500 MHz) and ^{13}C (126 MHz) spectra **1-amino-2-bromo-4-(3-ethylphenylamino)anthracene-9,10-dione (53)** in DMSO-d_6

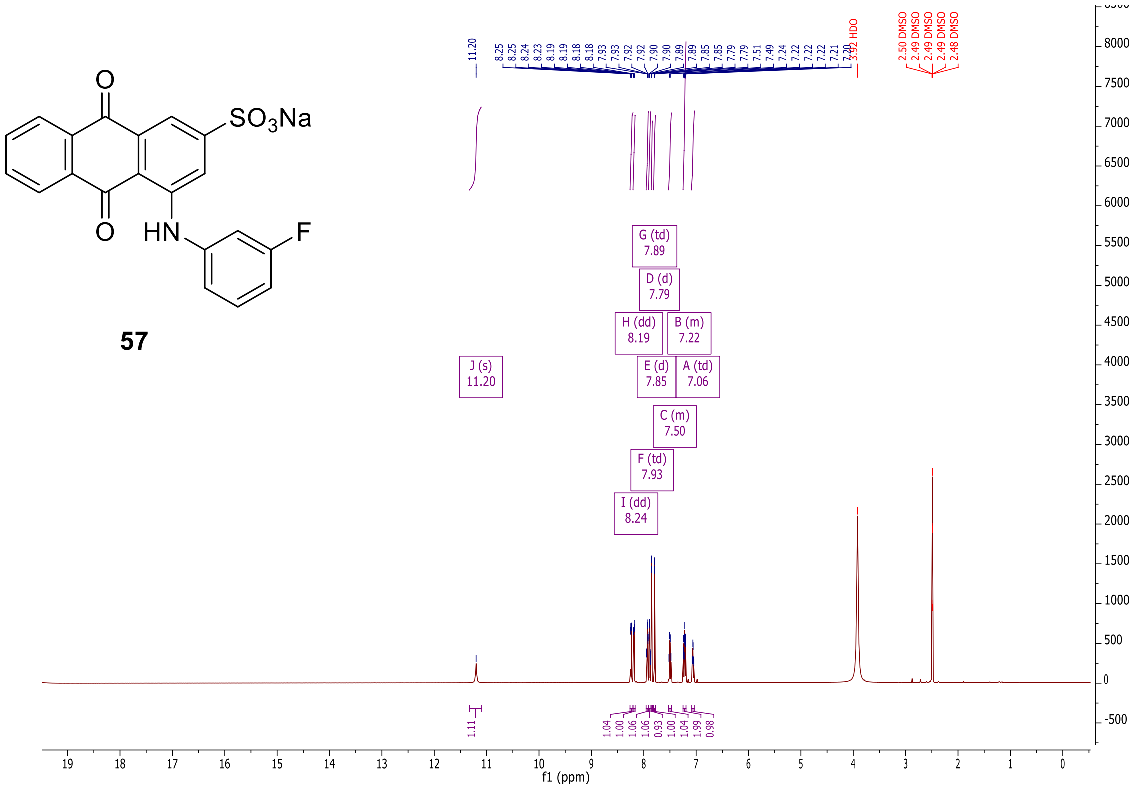

57

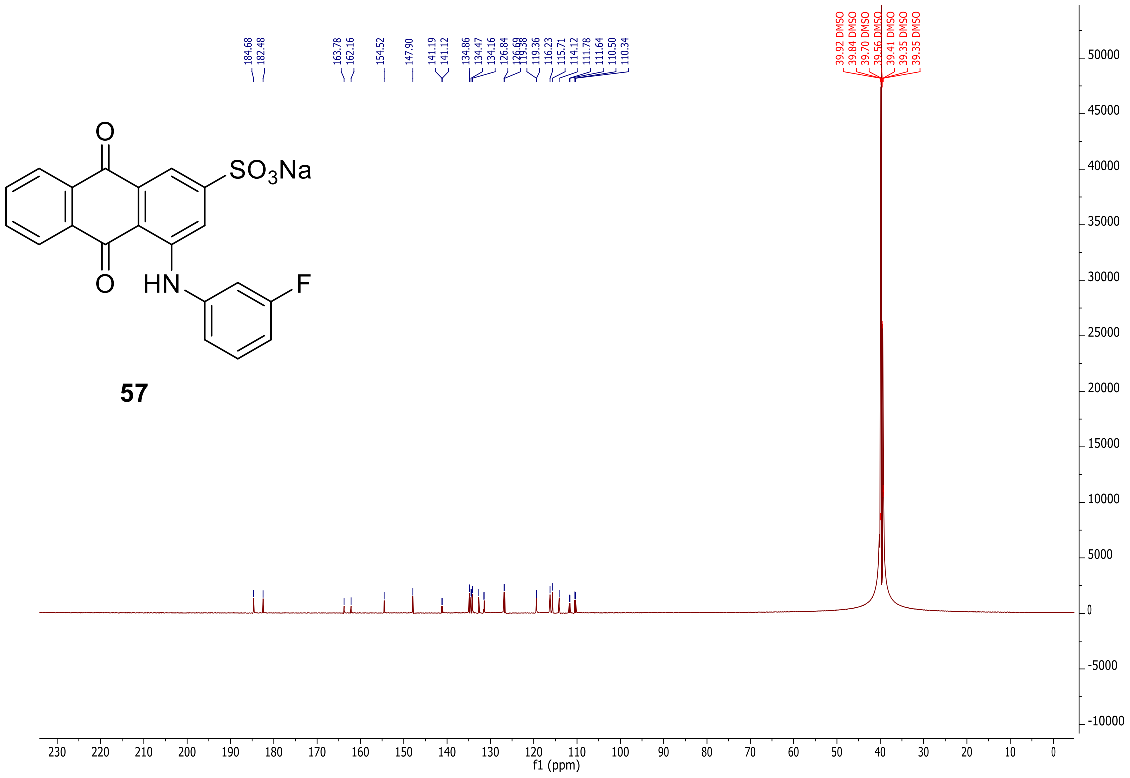

57

Figure S14. ^{1}H (500 MHz) and ^{13}C (126 MHz) spectra **4-(3-Fluorophenylamino)-9,10-dioxo-9,10-dihydroanthracene-2-sulfonic acid (57)** in DMSO-d_6

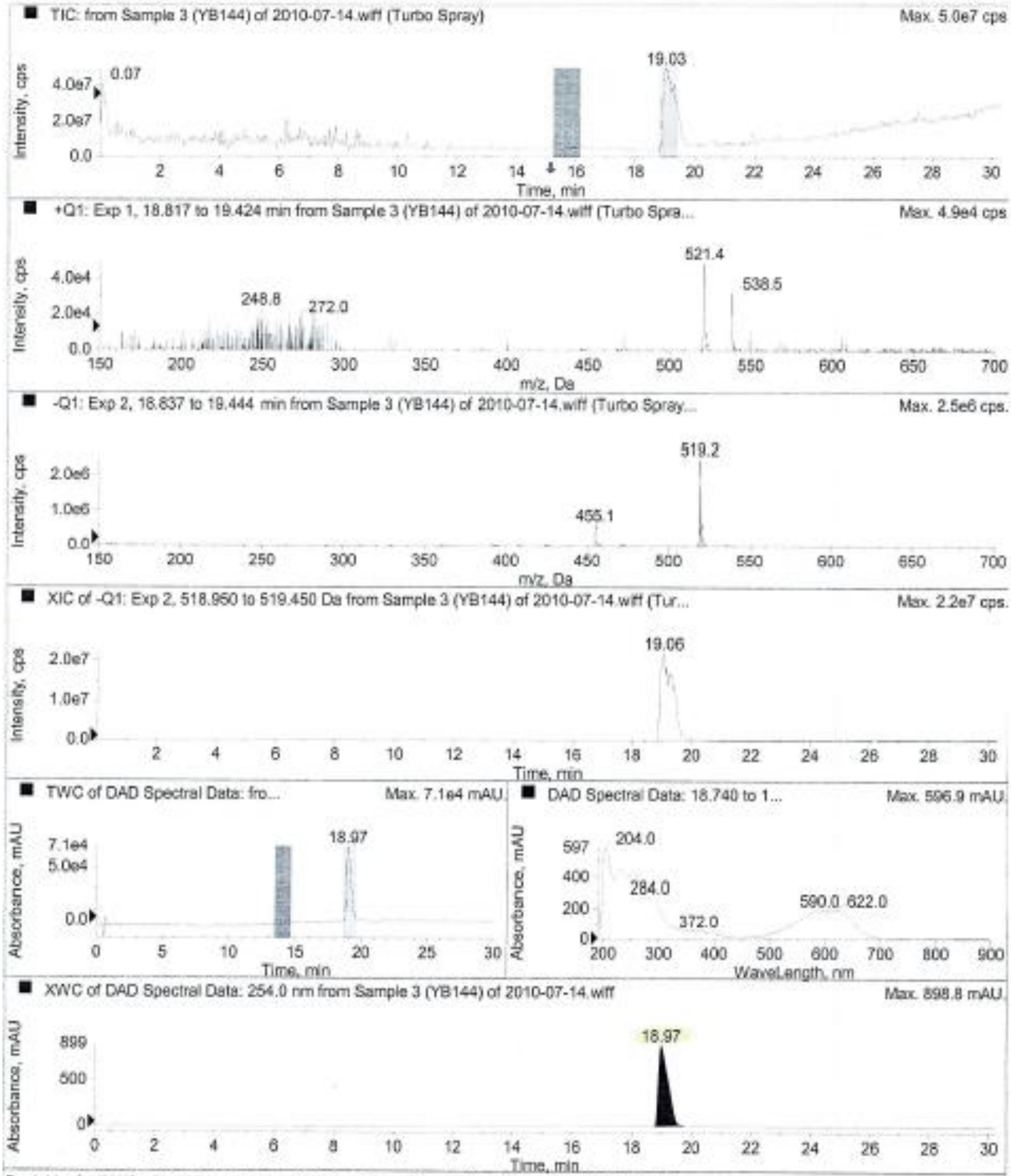

Peak List for "XWC of DAD Spectral Data: 254.0 nm from Sample 3 (YB144) of 2010-07-14.wiff"

	Time (min)	Area (mAU x min)	% Area	Height (mAU)	% Height	Width (min)	Baseline Type
1	18.9683	2.0104e4	100.0000	890.9180	100.0000	1.0600	Base to Base

Figure S15. LC-MS spectrum of compound **23***

* The purity of compound **23** is 100% (retention time: 18.97 belongs to the desired compound **23**).

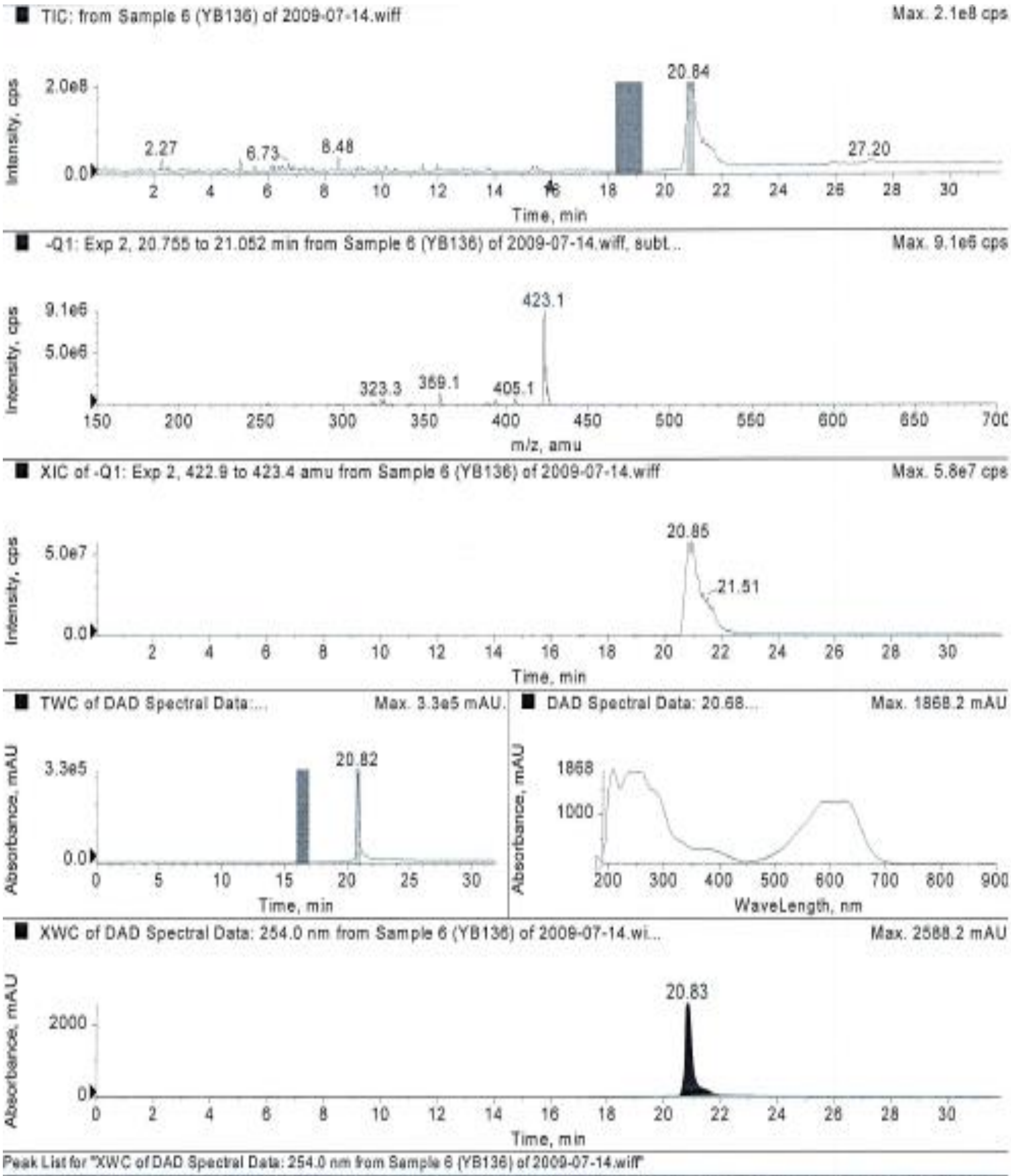

Peak List for "XWC of DAD Spectral Data: 254.0 nm from Sample 6 (YB136) of 2009-07-14.wiff"

	Time (min)	Area (mAU x min)	% Area	Height (mAU)	% Height	Width (min)	Baseline Type
1	20.8302	5.2665e4	100.0000	2563.2121	100.0000	1.5200	Base to Base

Figure S16. LC-MS spectrum of compound **27***

* The purity of compound **27** is 100% (retention time: 20.83 belongs to the desired compound **27**).

28).

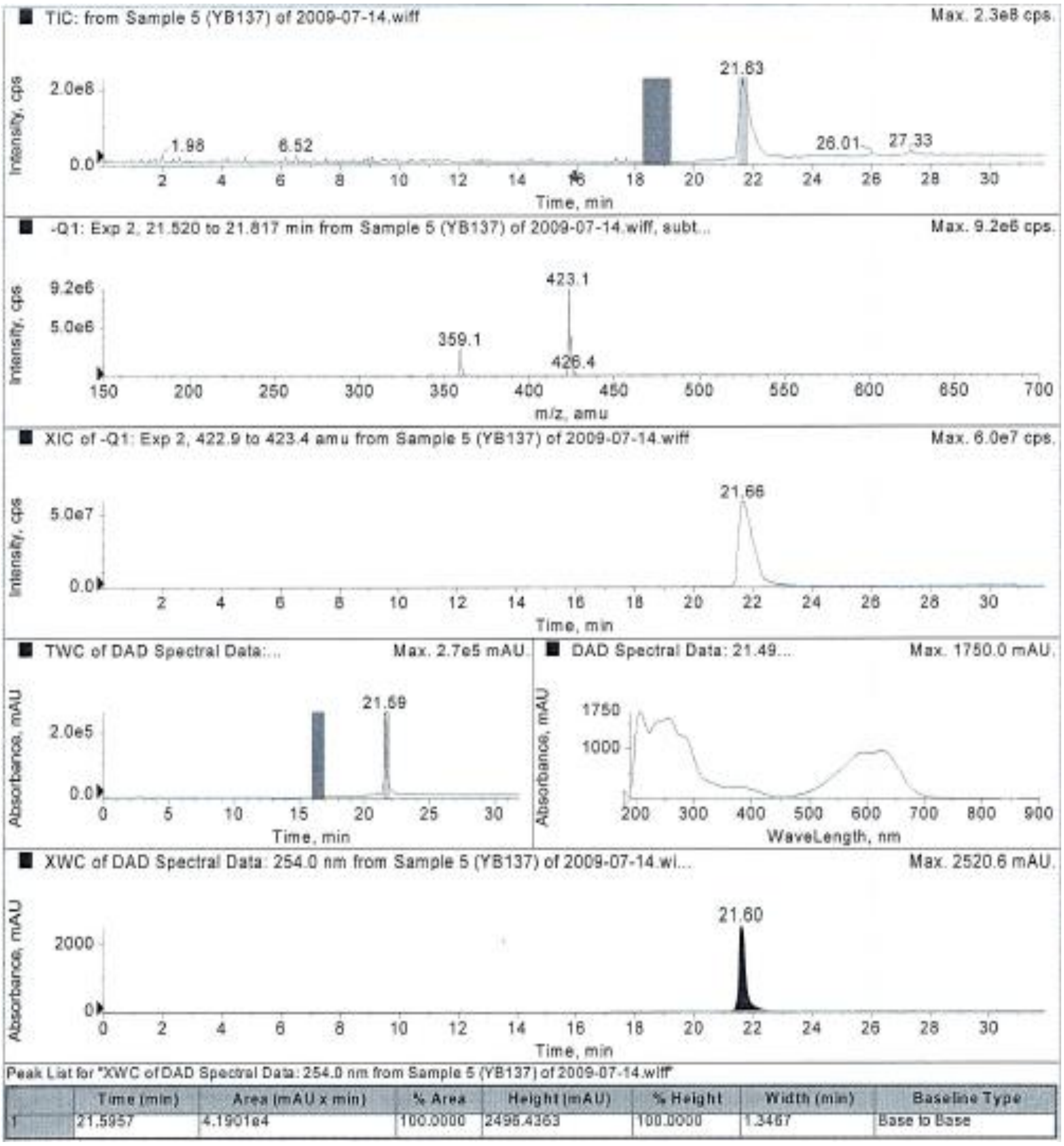

Peak List for "XWC of DAD Spectral Data: 254.0 nm from Sample 5 (YB137) of 2009-07-14.wiff"

	Time (min)	Area (mAU x min)	% Area	Height (mAU)	% Height	Width (min)	Baseline Type
1	21.5957	4.1901e4	100.0000	2496.4363	100.0000	1.3467	Base to Base

Figure S17. LC-MS spectrum of compound **28***

* The purity of compound **28** is 100% (retention time: 21.60 belongs to the desired compound **28**).

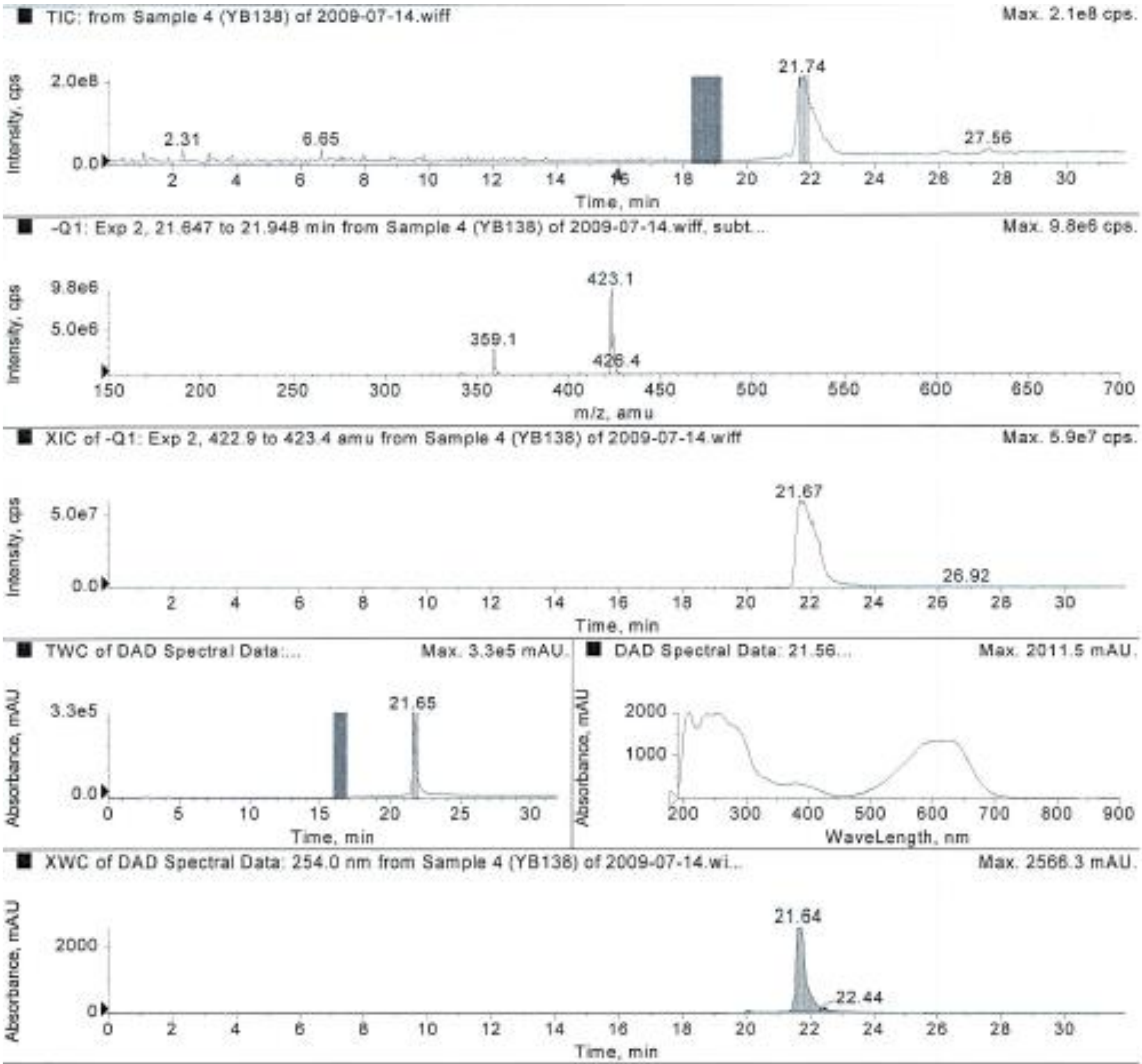

Peak List for "XWC of DAD Spectral Data: 254.0 nm from Sample 4 (YB138) of 2009-07-14.wiff"

	Time (min)	Area (mAU x min)	% Area	Height (mAU)	% Height	Width (min)	Baseline Type
1	20.0530	419.6614	0.7195	49.6579	1.8806	0.3867	Base to Base
2	21.6374	5.7397e4	98.4039	2533.6497	95.9518	1.0400	Expo. Skim
3	22.4407	511.3272	0.8766	57.2369	2.1676	0.5733	Expo. Daughter

Figure S18. LC-MS spectrum of compound **29***

* The purity of compound **29** is 98% (retention time: 21.60 belongs to the desired compound **29**).

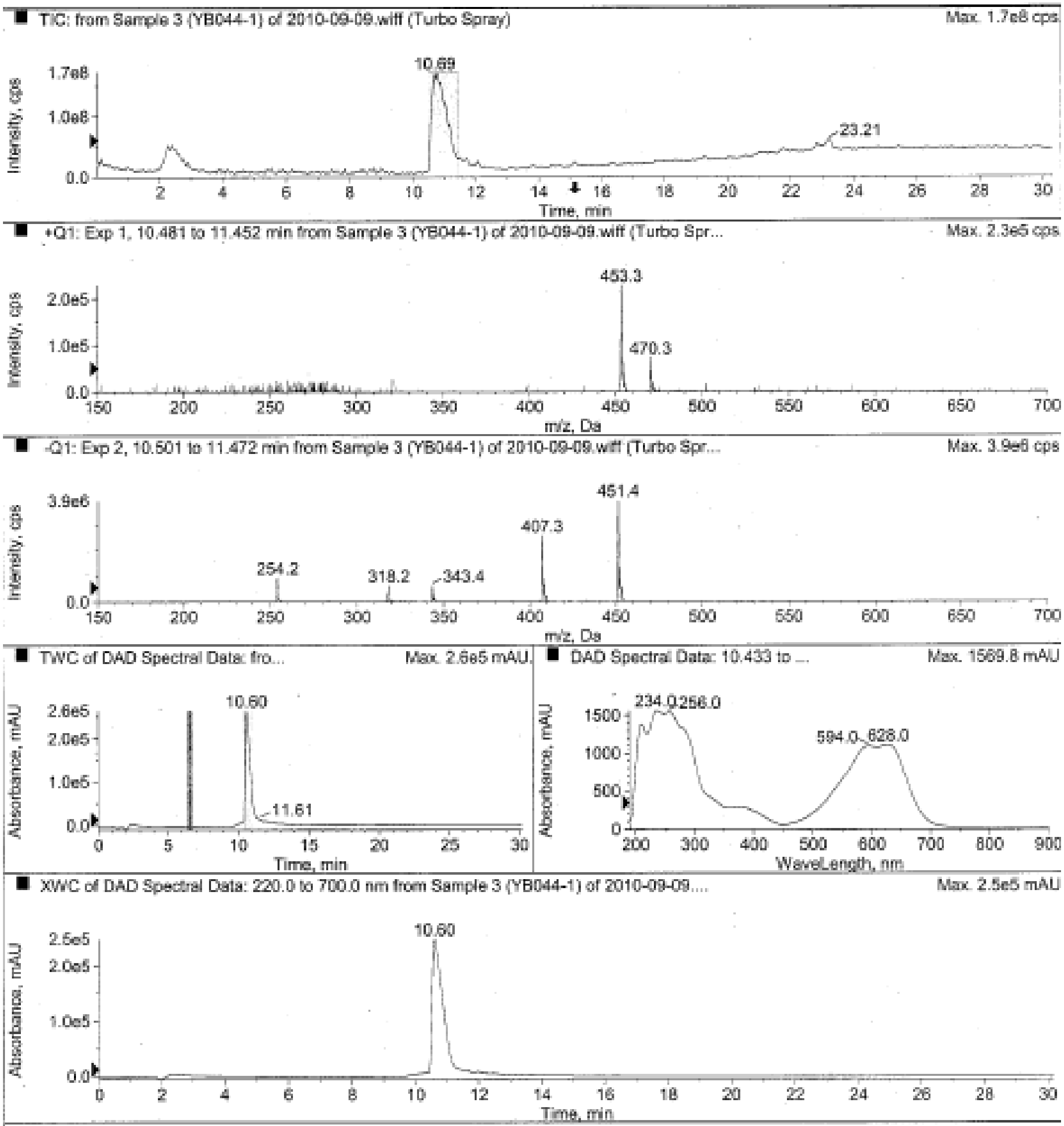

Peak List for "XWC of DAD Spectral Data: 220.0 to 700.0 nm from Sample 3 (YB044-1) of 2010-09-09.wiff"

	Time (min)	Area (mAU x min)	%Area	Height (mAU)	%Height	Width (min)	Baseline Type
1	1.0617	2430.2213	0.0422	175.0838	0.0697	0.2133	Base to Base
2	1.7849	2.0160e4	0.3497	1960.8161	0.7802	0.2600	Base to Base
3	2.3153	9240.2113	0.1603	310.4541	0.1235	0.3400	Base to Base
4	8.4048	1.5985e4	0.2773	81.3826	0.0324	0.8933	Base to Base
5	10.5973	5.6597e6	98.1781	2.4331e5	96.8077	2.0533	Base to Base
6	11.6151	1.0787e4	0.1871	1966.5168	0.7824	0.2133	Base to Base
7	11.9915	1.2677e4	0.2199	1815.6514	0.7224	0.3400	Base to Base
8	12.4967	8739.2810	0.1516	715.2935	0.2846	0.5067	Base to Base
9	13.1226	1.4818e4	0.2570	741.2078	0.2949	0.7267	Base to Base
10	13.8510	1.0190e4	0.1768	257.0132	0.1023	0.9333	Base to Base

Figure S19. LC-MS spectrum of compound **30***

* The purity of compound **30** is 100% (retention time: 10.60 belongs to the desired compound **30**).

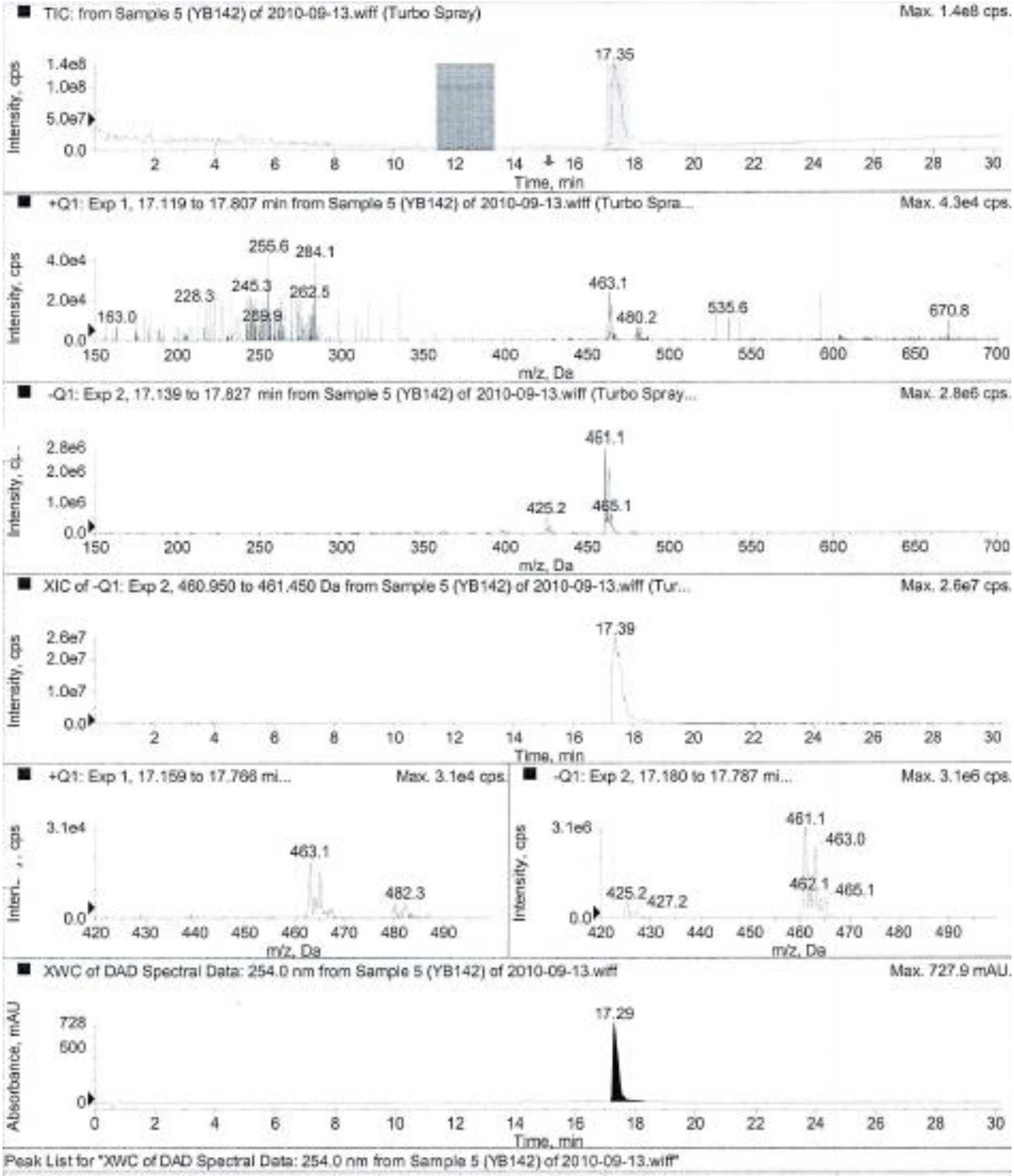

Peak List for "XWC of DAD Spectral Data: 254.0 nm from Sample 5 (YB142) of 2010-09-13.wiff"

	Time (min)	Area (mAU x min)	% Area	Height (mAU)	% Height	Width (min)	Baseline Type
1	17.2860	9301.6655	100.0000	716.5473	100.0000	1.1733	Base to Base

Figure S20. LC-MS spectrum of compound **34***

* The purity of compound **34** is 100% (retention time: 17.29 belongs to the desired compound **34**).

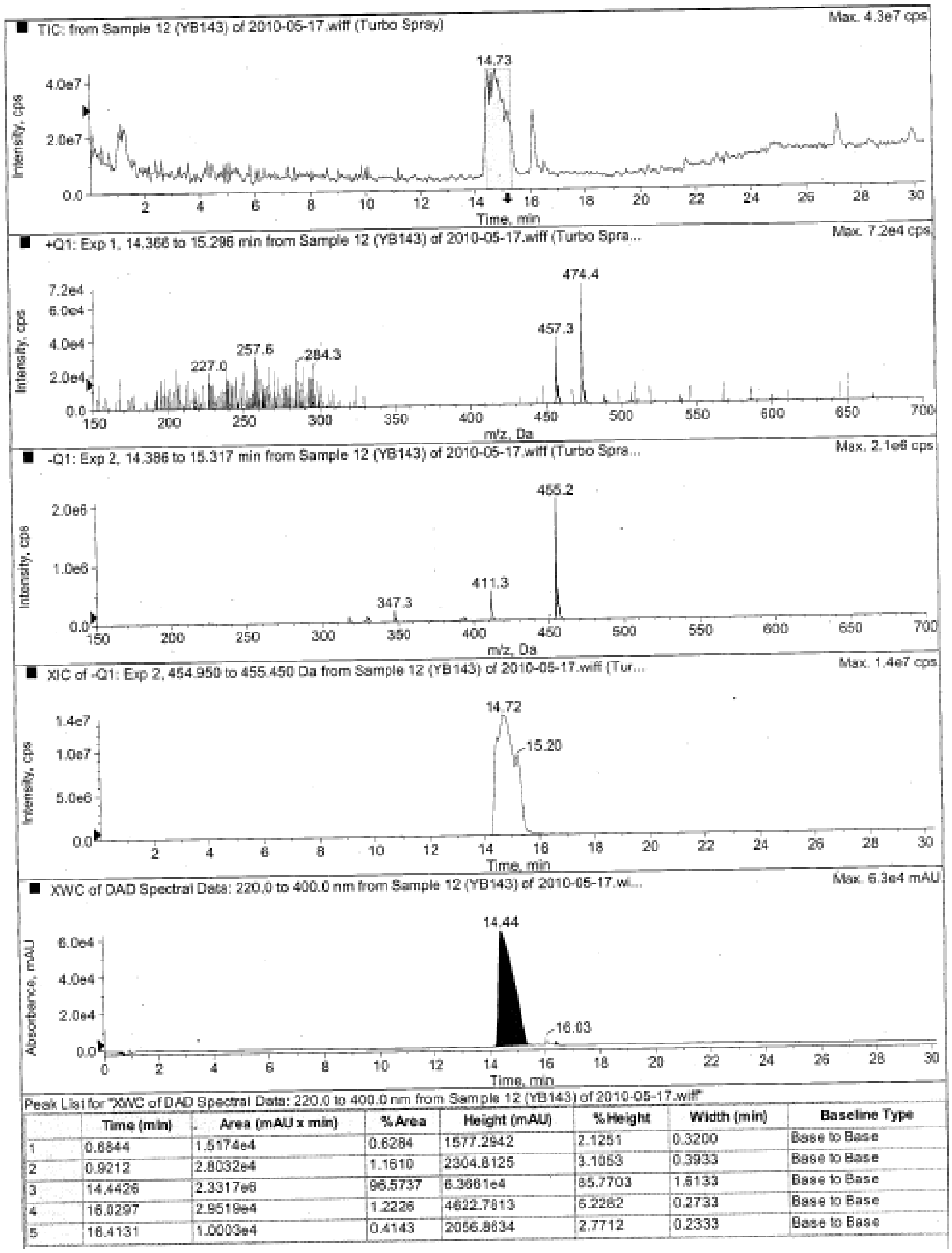

Peak List for "XWC of DAD Spectral Data: 220.0 to 400.0 nm from Sample 12 (YB143) of 2010-05-17.wiff"

	Time (min)	Area (mAU x min)	%Area	Height (mAU)	%Height	Width (min)	Baseline Type
1	0.6844	1.5174e4	0.6284	1577.2942	2.1251	0.3200	Base to Base
2	0.9212	2.8032e4	1.1610	2304.8125	3.1053	0.3933	Base to Base
3	14.4426	2.3317e6	96.5737	6.3681e4	85.7703	1.6133	Base to Base
4	16.0297	2.9519e4	1.2226	4622.7813	6.2262	0.2733	Base to Base
5	16.4131	1.0003e4	0.4143	2056.8634	2.7712	0.2333	Base to Base

Figure S21. LC-MS spectrum of compound **35***

* The purity of compound **35** is 98% (retention time: 14.44 belongs to the desired compound **35**).

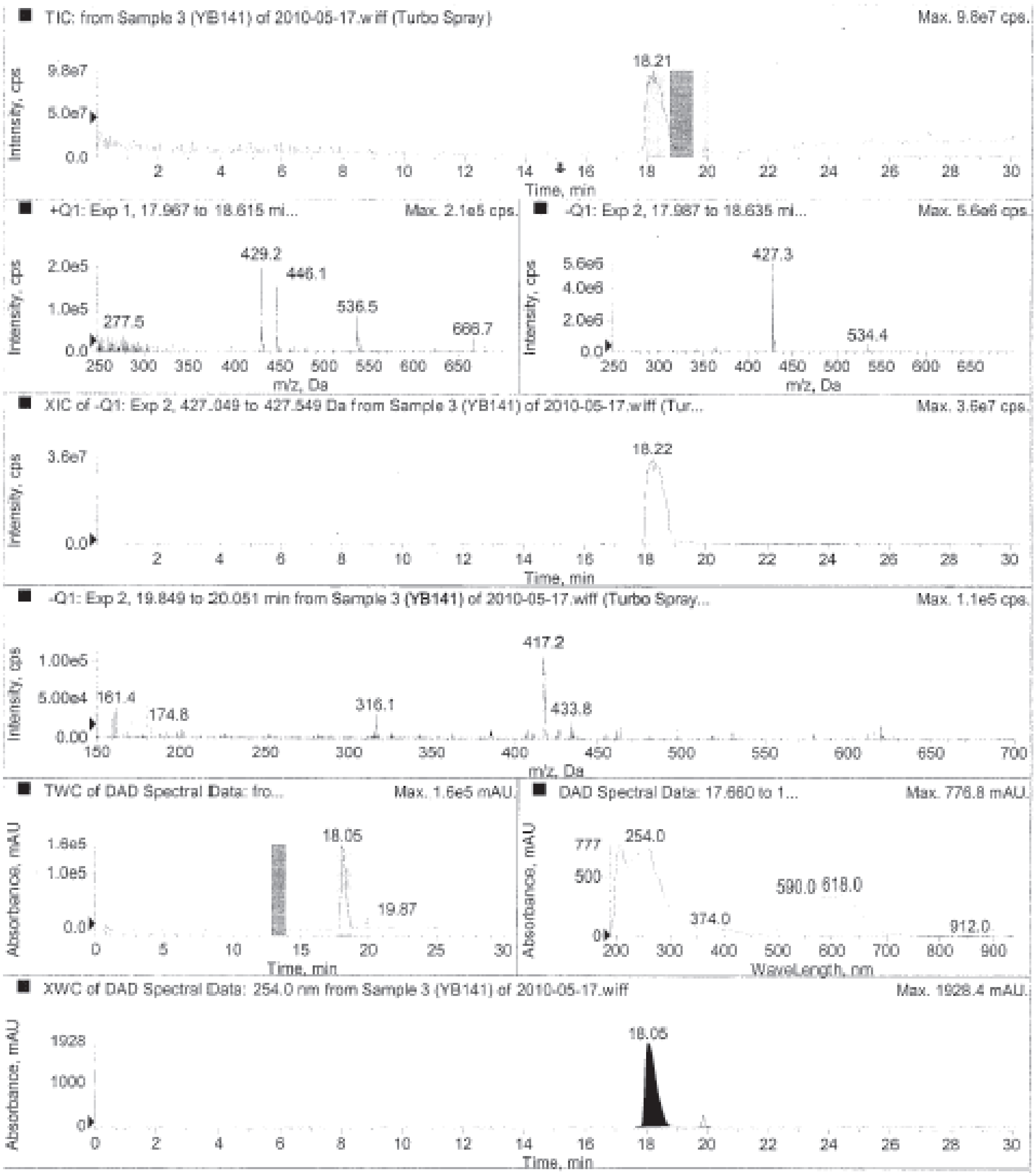

Peak List for "XWC of DAD Spectral Data: 254.0 nm from Sample 3 (YB141) of 2010-05-17.wiff"

	Time (min)	Area (mAU x min)	% Area	Height (mAU)	% Height	Width (min)	Baseline Type
1	18.0506	4.9762e4	96.8127	1915.7166	88.5100	1.1933	Valley
2	19.8642	1638.2708	3.1873	248.6901	11.4900	0.3933	Valley

Figure S22. LC-MS spectrum of compound **37***

* The purity of compound **37** is 97% (retention time: 18.06 belongs to the desired compound **37**).

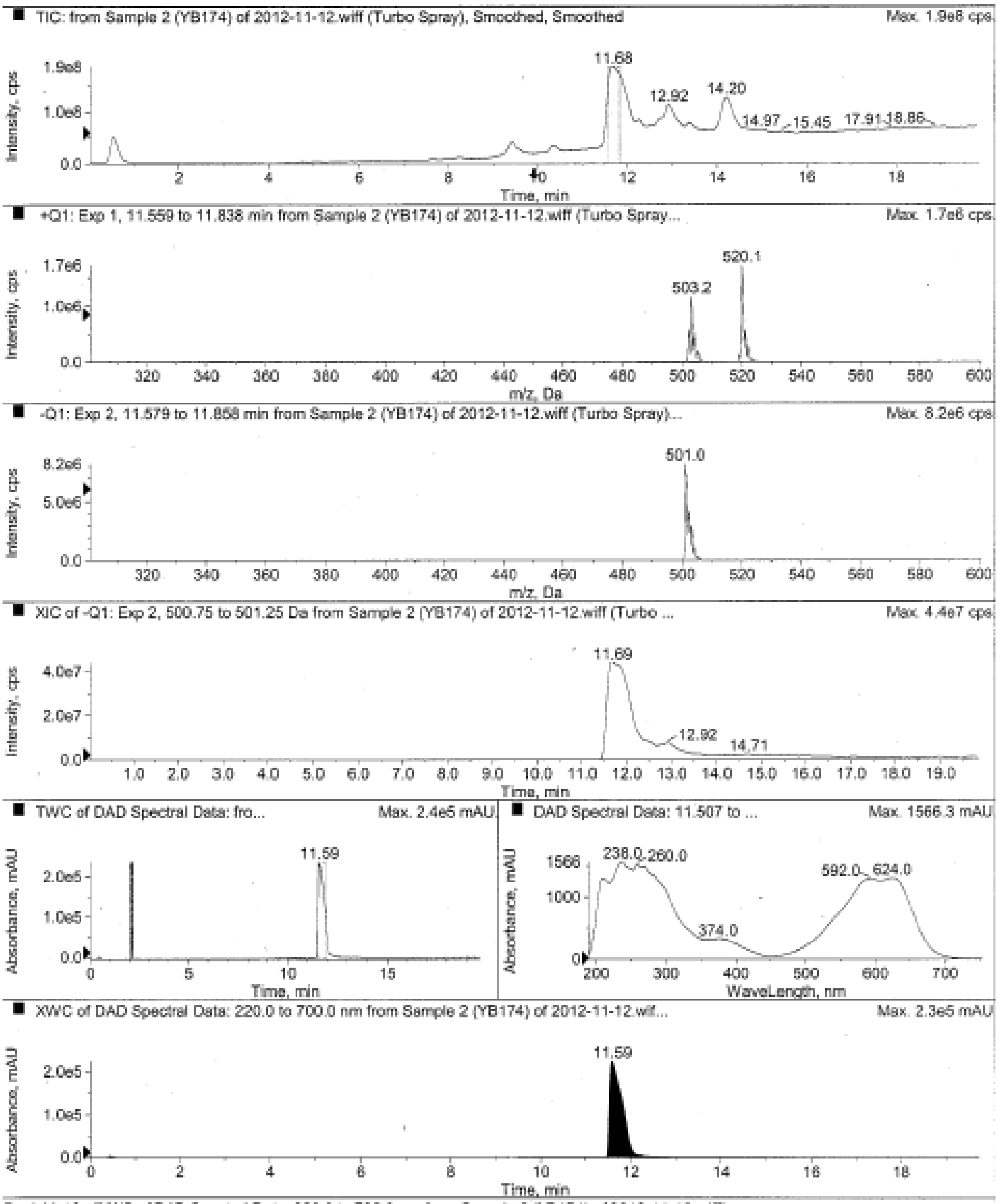

Peak List for "XWC of DAD Spectral Data: 220.0 to 700.0 nm from Sample 2 (YB174) of 2012-11-12.wiff"

	Time (min)	Area (mAU x min)	% Area	Height (mAU)	% Height	Width (min)	Baseline Type
1	0.4214	2.3886e4	0.5178	3804.3724	1.6545	0.2533	Base to Base
2	9.5466	2904.9649	0.0630	459.6964	0.1999	0.4333	Base to Base
3	11.5940	4.5861e6	99.4192	2.2567e5	98.1455	2.2000	Base to Base

Figure S23. LC-MS spectrum of compound **41***

* The purity of compound **41** is 99.4% (retention time: 11.59 belongs to the desired compound **41**).

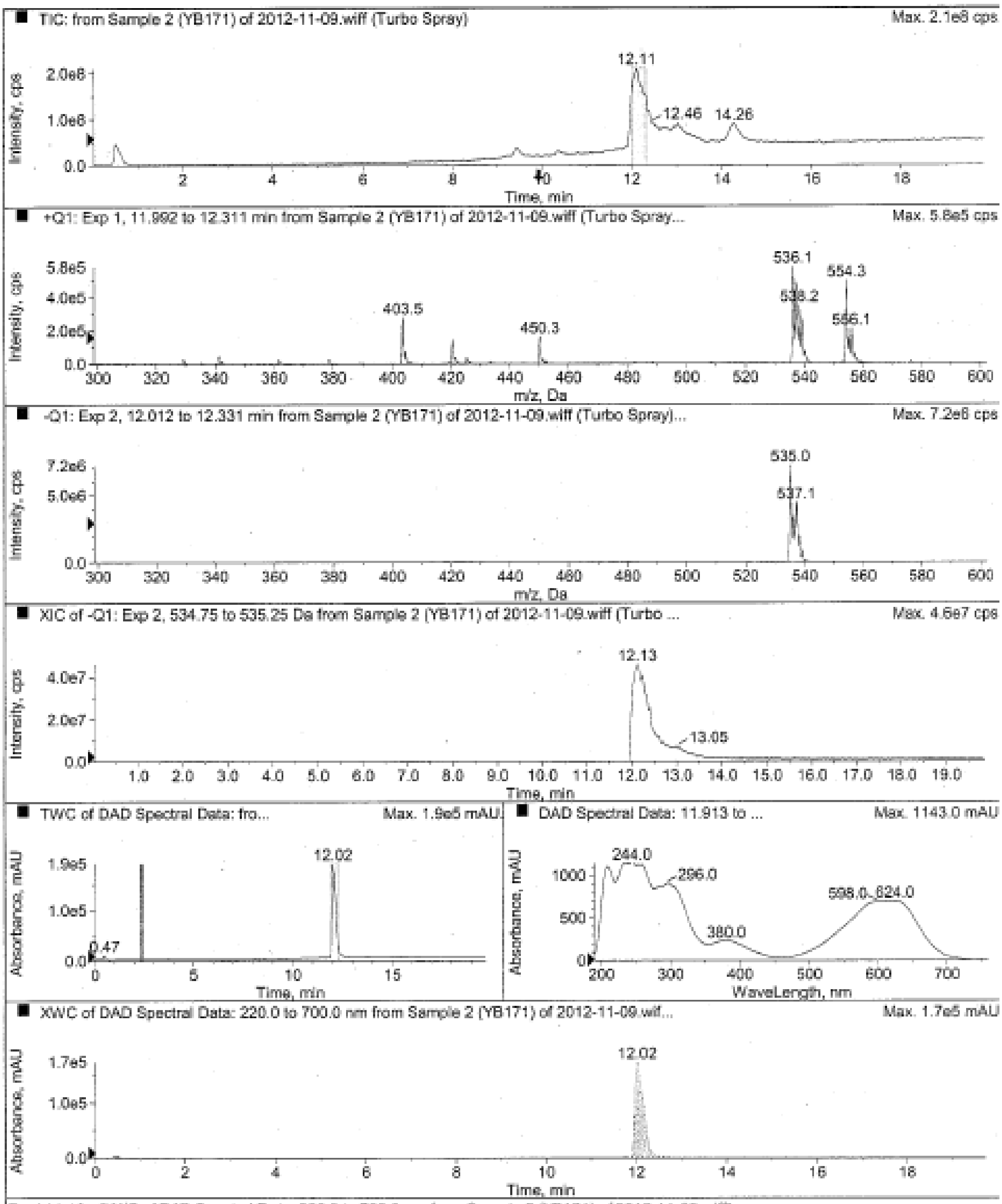

Peak List for "XWC of DAD Spectral Data: 220.0 to 700.0 nm from Sample 2 (YB171) of 2012-11-09.wiff"

	Time (min)	Area (mAU x min)	% Area	Height (mAU)	% Height	Width (min)	Baseline Type
1	0.4746	2.1634e4	0.8925	4714.4938	2.6452	0.2000	Base to Base
2	12.0201	2.4024e6	99.1075	1.7351e5	97.3548	2.0133	Base to Base

Figure S24. LC-MS spectrum of compound **45***

* The purity of compound **45** is 99% (retention time: 12.02 belongs to the desired compound **45**).

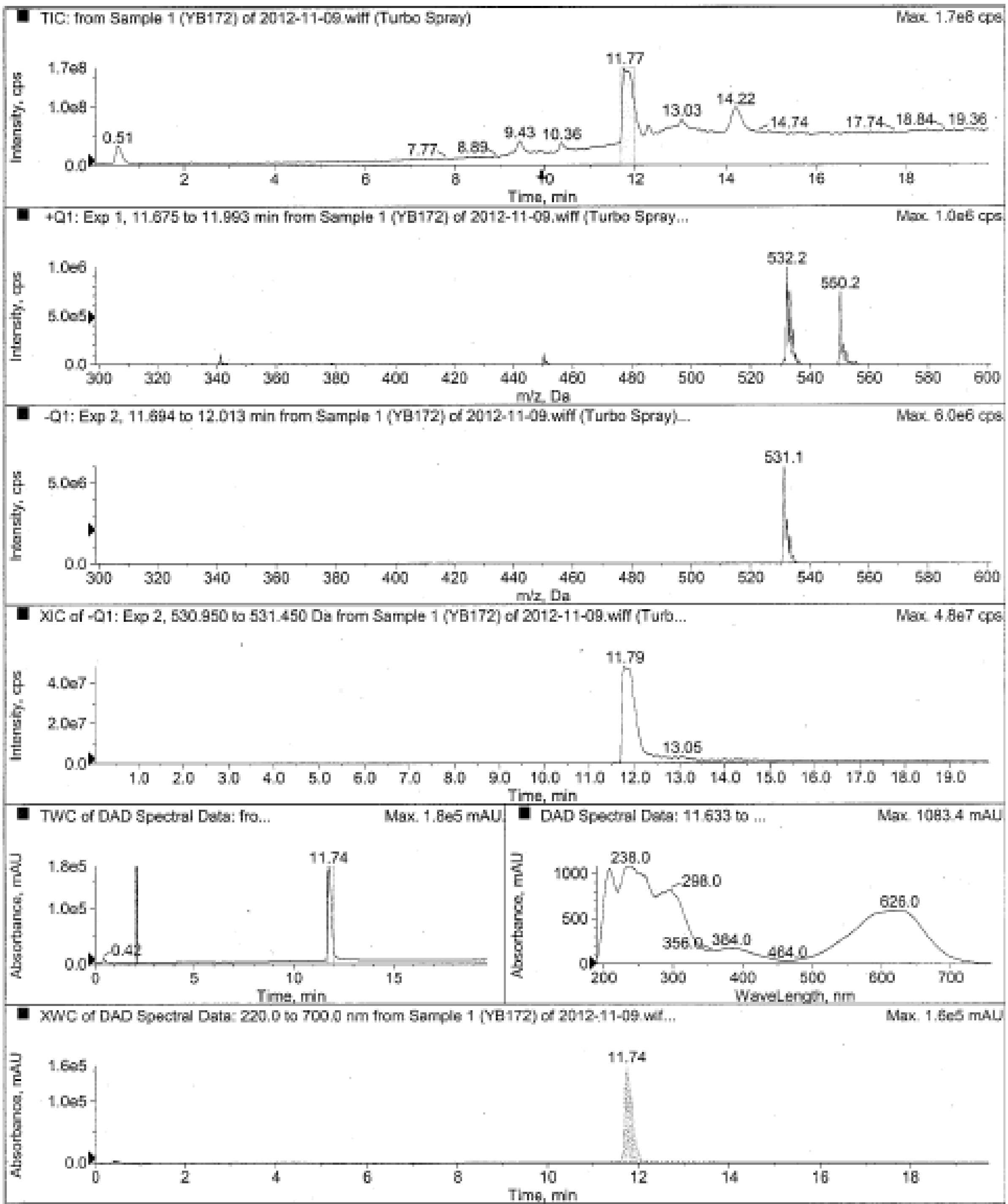

Peak List for "XWC of DAD Spectral Data: 220.0 to 700.0 nm from Sample 1 (YB172) of 2012-11-09.wiff"

	Time (min)	Area (mAU x min)	% Area	Height (mAU)	% Height	Width (min)	Baseline Type
1	0.4524	2.7575e4	1.3289	3667.4707	2.2781	0.2533	Base to Base
2	11.7402	2.0474e6	98.6711	1.5732e5	97.7219	1.9867	Base to Base

Figure S25. LC-MS spectrum of compound **47***

* The purity of compound **47** is 98.9% (retention time: 11.74 belongs to the desired compound **47**).

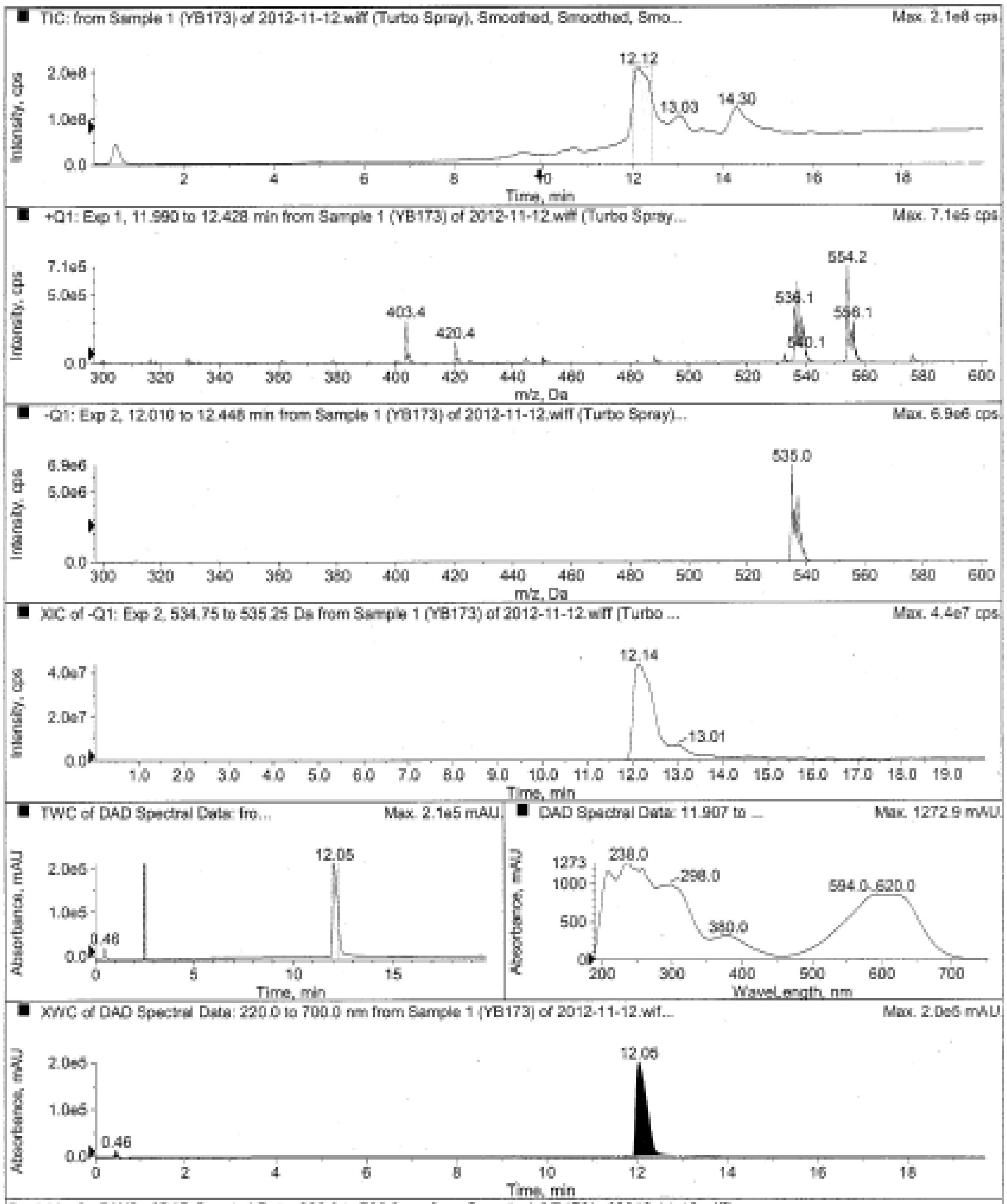

Peak List for "XWC of DAD Spectral Data: 220.0 to 700.0 nm from Sample 1 (YB173) of 2012-11-12.wiff"

	Time (min)	Area (mAU x min)	%Area	Height (mAU)	%Height	Width (min)	Baseline Type
1	0.4600	5.6183e4	1.6331	1.4174e4	6.5545	0.2267	Base to Base
2	0.8609	1.5293e4	0.4446	76.1261	0.0352	0.2333	Base to Base
3	9.5831	2345.3443	0.0682	227.4179	0.1052	0.4600	Base to Base
4	11.5778	4233.0203	0.1230	682.4129	0.3156	0.2467	Base to Base
5	12.0539	3.3592e6	97.6472	2.0061e5	92.7674	0.9800	Base to Base
6	12.8816	2884.6811	0.0839	480.4923	0.2222	0.2133	Base to Base

Figure S26. LC-MS spectrum of compound **48***

* The purity of compound **48** is 97.7% (retention time: 12.06 belongs to the desired compound **48**).

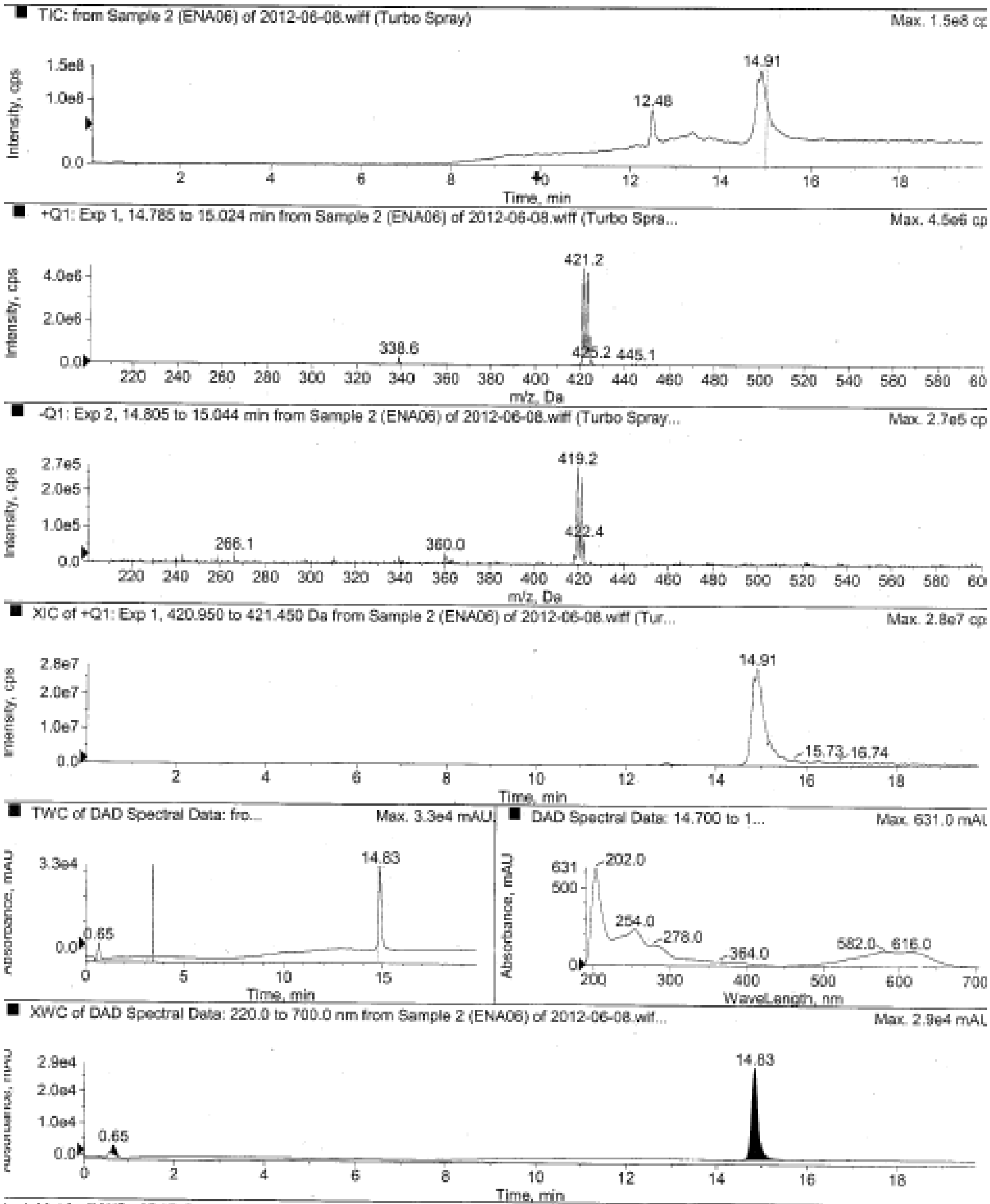

Peak List for "XWC of DAD Spectral Data: 220.0 to 700.0 nm from Sample 2 (ENA06) of 2012-06-08.wiff"

	Time (min)	Area (mAU x min)	%Area	Height (mAU)	%Height	Width (min)	Baseline Type
1	0.6477	1.7877e4	5.3561	3108.2313	9.6219	0.2133	Base to Base
2	13.3340	1784.0240	0.5345	265.5743	0.8221	0.3933	Base to Base
3	14.8255	3.1411e5	94.1094	2.8930e4	89.5560	1.2600	Base to Base

Figure S27. LC-MS spectrum of compound **53***

*The purity of compound **53** is 95% (retention time: 14.83 belongs to the desired compound **53**).

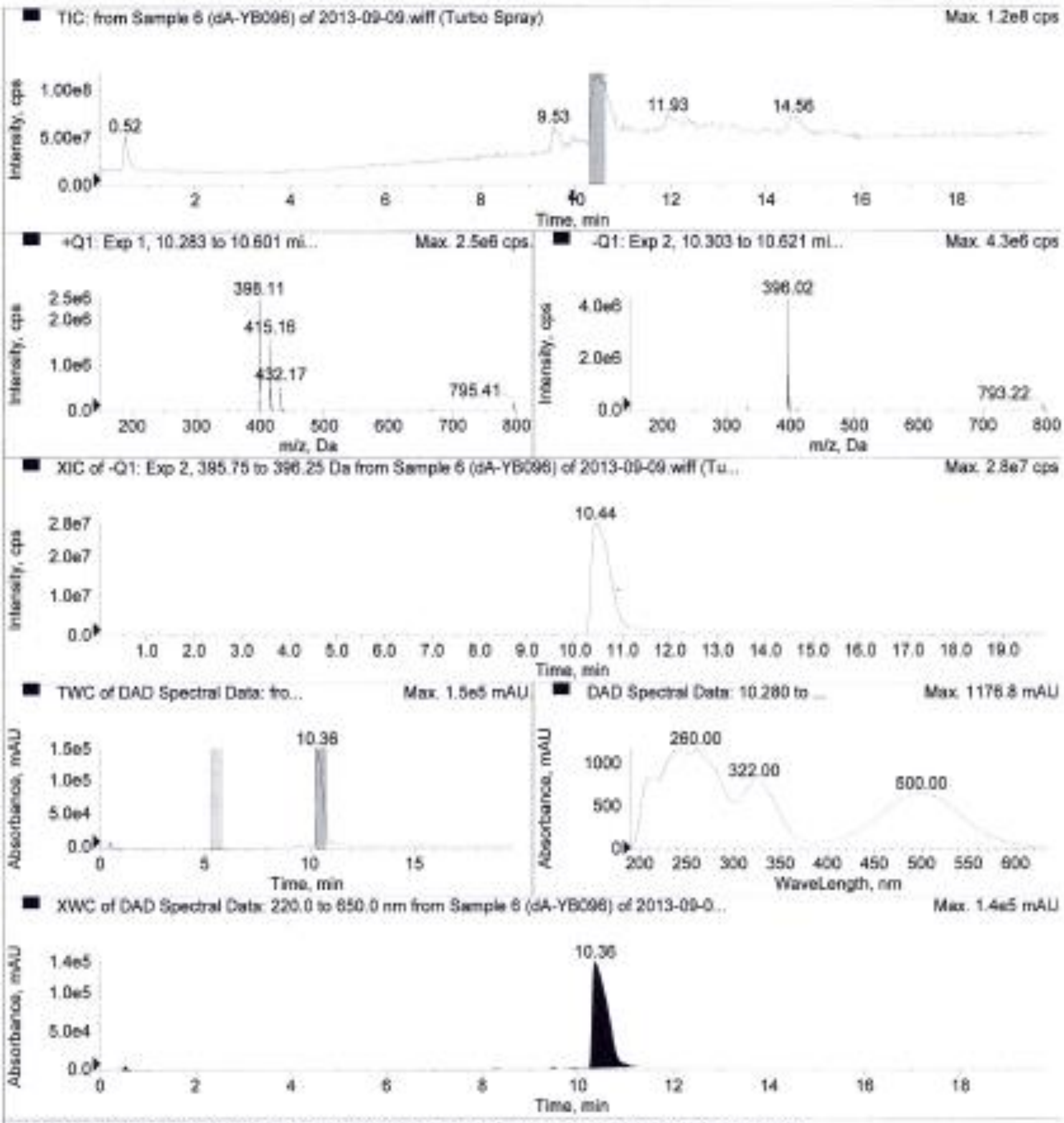

Peak List for "XWC of DAD Spectral Data: 220.0 to 650.0 nm from Sample 6 (dA-YB096) of 2013-09-09.wiff"

	Time (min)	Area (mAU x min)	% Area	Height (mAU)	% Height	Width (min)	Baseline Type
1	0.5189	2.4594e4	0.7999	5382.1916	3.8051	0.2000	Base to Base
2	8.2906	3456.6768	0.1124	559.2825	0.3746	0.2600	Base to Base
3	9.4819	7070.7348	0.2300	1449.2680	0.9707	0.2733	Base to Base
4	9.8573	2506.3652	0.0815	583.5370	0.3909	0.1200	Valley
5	9.9251	4660.2927	0.1516	861.1101	0.5768	0.1600	Valley
6	10.1388	2747.2974	0.0893	777.6059	0.5209	0.1400	Base to Base
7	10.3663	3.0298e6	98.5354	1.3968e5	93.5611	1.0600	Base to Base

Figure S28. LC-MS spectrum of compound **57***

* The purity of compound **57** is 99% (retention time: 10.36 belongs to the desired compound **57**).
